# Large-scale cross-cancer fine-mapping of the 5p15.33 region reveals multiple independent signals

**DOI:** 10.1016/j.xhgg.2021.100041

**Published:** 2021-06-12

**Authors:** Hongjie Chen, Arunabha Majumdar, Lu Wang, Siddhartha Kar, Kevin M. Brown, Helian Feng, Constance Turman, Joe Dennis, Douglas Easton, Kyriaki Michailidou, Jacques Simard, Breast Cancer Association Consortium (BCAC), Timothy Bishop, Iona C. Cheng, Jeroen R. Huyghe, Stephanie L. Schmit, Colorectal Transdisciplinary Study (CORECT), Colon Cancer Family Registry Study (CCFR), Genetics and Epidemiology of Colorectal Cancer Consortium (GECCO), Tracy A. O’Mara, Amanda B. Spurdle, Endometrial Cancer Association Consortium (ECAC), Puya Gharahkhani, Johannes Schumacher, Janusz Jankowski, Ines Gockel, Esophageal Cancer GWAS Consortium, Melissa L. Bondy, Richard S. Houlston, Robert B. Jenkins, Beatrice Melin, Glioma International Case Control Consortium (GICC), Corina Lesseur, Andy R. Ness, Brenda Diergaarde, Andrew F. Olshan, Head-Neck Cancer GWAS Consortium, Christopher I. Amos, David C. Christiani, Maria T. Landi, James D. McKay, International Lung Cancer Consortium (ILCCO), Myriam Brossard, Mark M. Iles, Matthew H. Law, Stuart MacGregor, Melanoma GWAS Consortium, Jonathan Beesley, Michelle R. Jones, Jonathan Tyrer, Stacey J. Winham, Ovarian Cancer Association Consortium (OCAC), Alison P. Klein, Gloria Petersen, Donghui Li, Brian M. Wolpin, Pancreatic Cancer Case-Control Consortium (PANC4), Pancreatic Cancer Cohort Consortium (PanScan), Rosalind A. Eeles, Christopher A. Haiman, Zsofia Kote-Jarai, Fredrick R. Schumacher, PRACTICAL consortium, CRUK, BPC3, CAPS, PEGASUS, Paul Brennan, Stephen J. Chanock, Valerie Gaborieau, Mark P. Purdue, Renal Cancer GWAS Consortium, Paul Pharoah, Rayjean J. Hung, Laufey T. Amundadottir, Peter Kraft, Bogdan Pasaniuc, Sara Lindström

**Affiliations:** 1 Department of Epidemiology, University of Washington, Seattle, WA, USA; 2 Department of Pathology and Laboratory Medicine, David Geffen School of Medicine, University of California, Los Angeles, Los Angeles, CA, USA; 3 Department of Mathematics, Indian Institute of Technology Hyderabad, Kandi, Telangana, India; 4 Department of Environmental and Occupational Health Sciences, University of Washington, Seattle, WA, USA; 5 Medical Research Council Integrative Epidemiology Unit, Population Health Sciences, Bristol Medical School, University of Bristol, Bristol, UK; 6 Division of Cancer Epidemiology and Genetics, National Cancer Institute, National Institutes of Health, Bethesda, MD, USA; 7 Department of Biostatistics, Harvard T.H. Chan School of Public Health, Boston, MA, USA; 8 Department of Epidemiology, Harvard T.H. Chan School of Public Health, Boston, MA, USA; 9 Centre for Cancer Genetic Epidemiology, Department of Public Health and Primary Care, University of Cambridge, Cambridge, UK; 10 Biostatistics Unit, The Cyprus Institute of Neurology and Genetics, Nicosia, Cyprus; 11 Cyprus School of Molecular Medicine, Nicosia, Cyprus; 12 Department of Molecular Medicine, Faculty of Medicine, Université Laval and Centre de recherche du CHU de Québec-Université Laval, Québec, QC, Canada; 13 Leeds Institute of Cancer and Pathology, University of Leeds, Leeds, UK; 14 Department of Epidemiology and Biostatistics, University of California at San Francisco, San Francisco, CA, USA; 15 Public Health Sciences Division, Fred Hutchinson Cancer Research Center, Seattle, WA, USA; 16 Genomic Medicine Institute, Lerner Research Institute, Cleveland Clinic, Cleveland, OH, USA; 17 Department of Genetics and Computational Biology, QIMR Berghofer Medical Research Institute, Brisbane, Australia; 18 Statistical Genetics, QIMR Berghofer Medical Research Institute, Brisbane, Australia; 19 Center for Human Genetics, University Hospital of Marburg, Marburg, Germany; 20 College of Medicine and Health Sciences, United Arab Emirates University, Al Ain, Abu Dhabi, UAE; 21 Comprehensive Clinical Trials Unit, University College London, London, UK; 22 Department of Visceral, Transplant, Thoracic, and Vascular Surgery, University Hospital of Leipzig, Leipzig, Germany; 23 Department of Epidemiology and Population Health, Stanford University, Palo Alto, CA, USA; 24 Division of Genetics and Epidemiology, The Institute of Cancer Research, London, UK; 25 Department of Laboratory Medicine and Pathology, Mayo Clinic Comprehensive Cancer Center, Mayo Clinic, Rochester, MN, USA; 26 Department of Radiation Sciences, Umeå University, Umeå, Sweden; 27 Department of Environmental Medicine and Public Health, Icahn School of Medicine at Mount Sinai, New York, NY, USA; 28 International Agency for Research on Cancer, World Health Organization, Lyon, France; 29 National Institute for Health Research (NIHR) Bristol Biomedical Research Centre, University Hospitals Bristol and Weston NHS Foundation Trust and the University of Bristol, Bristol, UK; 30 Bristol Dental School, University of Bristol, Bristol, UK; 31 Department of Human Genetics, Graduate School of Public Health, University of Pittsburgh, Pittsburgh, PA, USA; 32 UPMC Hillman Cancer Center, Pittsburgh, PA, USA; 33 Department of Epidemiology, Gillings School of Global Public Health, University of North Carolina at Chapel Hill, Chapel Hill, NC, USA; 34 UNC Lineberger Comprehensive Cancer Center, Chapel Hill, NC, USA; 35 Institute for Clinical and Translational Research, Baylor College of Medicine, Houston, TX, USA; 36 Department of Environmental Health, Harvard T.H. Chan School of Public Health, Boston, MA, USA; 37 Genetic Epidemiology and Functional Genomics of Multifactorial Diseases Team, Institut National de la Santé et de la Recherche Médicale (INSERM), UMRS-1124, Université Paris Descartes, Paris, France; 38 Prosserman Centre for Population Health Research, Lunenfeld-Tanenbuaum Research Institute, Sinai Health System, Toronto, ON, Canada; 39 Leeds Institute for Data Analytics, University of Leeds, Leeds, UK; 40 School of Biomedical Sciences, Faculty of Health, and Institute of Health and Biomedical Innovation, Queensland University of Technology, Kelvin Grove, QLD, Australia; 41 Center for Bioinformatics and Functional Genomics, Department of Biomedical Sciences, Cedars-Sinai Medical Center, Los Angeles, CA, USA; 42 Department of Oncology, University of Cambridge, Cambridge, UK; 43 Department of Health Science Research, Mayo Clinic, Rochester, MN, USA; 44 Department of Oncology, Sidney Kimmel Comprehensive Cancer Center, Johns Hopkins School of Medicine, Baltimore, MD, USA; 45 Department of Pathology, Sol Goldman Pancreatic Cancer Research Center, Johns Hopkins School of Medicine, Baltimore, MD, USA; 46 Department of Gastrointestinal Medical Oncology, University of Texas MD Anderson Cancer Center, Houston, TX, USA; 47 Department of Medical Oncology, Dana Farber Harvard Cancer Center, Boston, MA, USA; 48 Oncogenetics Team, Division of Genetics and Epidemiology, The Institute of Cancer Research, London, UK; 49 Cancer Genetics Unit, Royal Marsden NHS Foundation Trust, London, UK; 50 Department of Preventive Medicine, University of Southern California, Los Angeles, CA, USA; 51 Department of Epidemiology and Biostatistics, Case Western Reserve University, Cleveland, OH, USA; 52 Seidman Cancer Center, University Hospitals, Cleveland, OH, USA; 53 Department of Human Genetics, David Geffen School of Medicine, University of California, Los Angeles, Los Angeles, CA, USA; 54 Department of Computational Medicine, David Geffen School of Medicine, University of California, Los Angeles, Los Angeles, CA, USA

**Keywords:** cancer, pleiotropy, fine-mapping, 5p15.33 region, *TERT*, *CLPTM1L*

## Abstract

Genome-wide association studies (GWASs) have identified thousands of cancer risk loci revealing many risk regions shared across multiple cancers. Characterizing the cross-cancer shared genetic basis can increase our understanding of global mechanisms of cancer development. In this study, we collected GWAS summary statistics based on up to 375,468 cancer cases and 530,521 controls for fourteen types of cancer, including breast (overall, estrogen receptor [ER]-positive, and ER-negative), colorectal, endometrial, esophageal, glioma, head/neck, lung, melanoma, ovarian, pancreatic, prostate, and renal cancer, to characterize the shared genetic basis of cancer risk. We identified thirteen pairs of cancers with statistically significant local genetic correlations across eight distinct genomic regions. Specifically, the 5p15.33 region, harboring the *TERT* and *CLPTM1L* genes, showed statistically significant local genetic correlations for multiple cancer pairs. We conducted a cross-cancer fine-mapping of the 5p15.33 region based on eight cancers that showed genome-wide significant associations in this region (ER-negative breast, colorectal, glioma, lung, melanoma, ovarian, pancreatic, and prostate cancer). We used an iterative analysis pipeline implementing a subset-based meta-analysis approach based on cancer-specific conditional analyses and identified ten independent cross-cancer associations within this region. For each signal, we conducted cross-cancer fine-mapping to prioritize the most plausible causal variants. Our findings provide a more in-depth understanding of the shared inherited basis across human cancers and expand our knowledge of the 5p15.33 region in carcinogenesis.

## Teaser

Genome-wide association studies (GWASs) have identified multiple susceptibility regions shared across cancers. Leveraging GWAS summary statistics for fourteen cancers, we observed pairwise local genetic correlations in the 5p15.33 region, harboring the *TERT* and *CLPTM1L* genes. Further analyses of this region revealed ten independent cross-cancer associations, for which the most plausible causal cancer risk variants were identified.

## Introduction

Cancer is a major global public health problem. More than 19.3 million new cancer cases and 10 million cancer deaths were estimated to occur worldwide in 2020.^1^ In the United States, approximately 1.9 million individuals are projected to be newly diagnosed with cancer, and more than 600,000 affected individuals are projected to die of cancer in 2021.^2^ Inherited genetic variants, along with environmental exposures, contribute substantially to the pathogenesis of cancers. Cancers tend to cluster in families, and twin studies have reported cancer-specific heritability ranging from 9% (head/neck) to 58% (melanoma).^3^^,^^4^

Genome-wide association studies (GWASs) of specific types of cancer have identified genetic loci significantly associated with susceptibility to malignancies. In a recent study of 18 types of cancer in European ancestry populations,^5^ the authors identified 17 genome-wide significant variants that were associated with the risk of at least two cancers with the same direction of effect. The 8q24 region has been long recognized as a pleiotropic locus, where genetic variants have been associated with the risk of breast, colorectal, endometrial, glioma, ovarian, pancreatic, and prostate cancer, among others.^6^, ^7^, ^8^, ^9^, ^10^, ^11^, ^12^, ^13^, ^14^, ^15^, ^16^ The 5p15.33 region has been associated with more than ten types of cancer, with multiple independent risk alleles identified.^17^, ^18^, ^19^, ^20^, ^21^, ^22^, ^23^, ^24^ Various biological mechanisms, including inflammation, epigenetics, gene expression, and telomere structure, have been proposed to explain these identified pleiotropic associations. For example, the 5p15.33 region harbors the *TERT* gene, which encodes the catalytic subunit of telomerase,^25^ as well as the *CLPTM1L* gene, which encodes the cleft lip and palate-associated transmembrane-1 like protein.^26^

In addition, recent efforts have been devoted toward estimating the genetic correlation between pairs of cancers. Using the restricted maximum likelihood (REML) approach implemented in the GCTA tool,^27^ one study quantified the pairwise genetic correlation among 13 types of cancers in European ancestry populations.^28^ Four pairs of cancers, including bladder-lung, testis-renal, lymphoma-osteosarcoma, and lymphoma-leukemia, demonstrated statistically significant shared heritability. We have previously applied linkage disequilibrium (LD) score regression^29^^,^^30^ on cancer GWAS summary statistics and observed significant genetic correlations between multiple solid tumor pairs, including colorectal-lung, colorectal-pancreatic, breast-colorectal, breast-lung, breast-ovarian, and lung-head/neck cancer.^31^^,^^32^ However, these studies only quantified the pairwise genetic correlation on a genome-wide scale, ignoring variations in the local genetic correlation across the genome. As shared heritability between cancers may not be uniformly distributed across the genome, such limitation may lead to missed opportunities to discover specific regions with crucial contribution to the oncogenesis of multiple cancers.^33^

In the present study, we collected European ancestry-derived GWAS summary statistics from large-scale meta-analysis results for 14 types of cancer, based on a total number of 375,468 cancer cases and 530,521 controls. By partitioning the genome into 1,703 blocks based on the LD pattern in the 1000 Genomes (1000G) European ancestry populations,^34^ we systematically estimated pairwise local genetic correlations between cancers. After adjusting for multiple comparisons, we identified thirteen pairs of cancers with statistically significant local genetic correlations across eight distinct genomic regions. Among these, a 1.2 Mb region at 5p15.33, harboring the *TERT* and *CLPTM1L* genes, showed significant local genetic correlations across six pairs of cancers, including breast (overall and estrogen receptor [ER]-negative), colorectal, glioma, lung, melanoma, pancreatic, and prostate cancer. We then utilized an iterative analysis pipeline implementing a subset-based meta-analysis approach (Association Analysis for SubSETs [ASSET])^35^ and a conditional analysis tool (COndition and JOint analysis tool implemented in the Genome-wide Complex Trait Analysis software, COJO-GCTA)^36^ and identified ten independent cross-cancer signals within the 1.2 Mb region. For each independent signal, we conducted multi-cancer fine-mapping analysis using PAINTOR^37^ to prioritize the variants with the highest posterior probability of being causal. Our study provides novel evidence of shared genetic susceptibility across cancer types and contributes crucial information toward understanding the common genetic mechanisms of carcinogenesis.

## Material and methods

### Study sample and genotype quality control

We collected the meta-analysis results from a total of 14 cancer GWASs: breast (overall, ER-positive, and ER-negative),^38^ colorectal,^39^ endometrial,^16^ esophageal,^40^ glioma,^41^ head/neck,^42^ lung,^43^ melanoma,^44^ ovarian,^45^ pancreatic,^46^ prostate,^47^ and renal cancer.^48^ Sample size for each cancer is listed in Table 1. The GWAS summary statistics for each cancer were provided by the corresponding collaborative consortia. Details on study characteristics and subjects contributing to each cancer-specific GWAS summary dataset have been described in the original cancer-specific publications. All the GWAS results used in this study were based on European ancestry populations. Genomic positions were based on Genome Reference Consortium GRCh37 (hg19).

**Table 1:** Overview of the cancer GWAS datasets included in this study

Cancer types	No. of cases	No. of controls	No. of SNPs after QC^a^	Reference
Breast, overall	122,977	105,974	9,934,907	Michailidou et al., 2017^38^
Breast, ER-negative	21,468	100,564	9,942,394	Michailidou et al., 2017^38^
Breast, ER-positive	69,501	95,042	10,267,258	Michailidou et al., 2017^38^
Colorectal	55,168	65,160	7,910,462	Huyghe et al., 2019^39^
Endometrial	12,906	108,979	11,595,492	O’Mara et al., 2018^16^
Esophageal	4,112	13,663	9,038,176	Gharahkhani et al., 2016^40^
Glioma	12,488	18,169	6,931,587	Melin et al., 2017^41^
Head/neck	6,034	6,585	7,471,918	Lesseur et al., 2016^42^
Lung	29,266	56,450	7,673,197	McKay et al., 2017^43^
Melanoma	12,814	23,203	7,748,523	Law et al., 2015^44^
Ovarian	22,406	40,951	9,870,154	Phelan et al., 2017^45^
Pancreatic	8,638	12,217	9,568,913	Klein et al.,2018^46^
Prostate	79,166	61,106	10,002,813	Schumacher et al., 2018^47^
Renal	10,784	20,407	8,362,393	Scelo et al., 2017^48^

a Filtered out variants with imputation quality score < 0.3, minor allele frequency (MAF) < 1%, and |log odds ratio| > 3.

Individual cancer GWASs were primarily imputed to the 1000G reference panels.^49^ Breast, ovarian, pancreatic, and prostate cancer used the 1000G phase 3 v.5 reference panel; colorectal cancer used the Haplotype Reference Consortium (HRC); head/neck cancer used HRC; renal cancer used 1000G phase 1 v.3; metanalysis results for melanoma GWASs were based on studies majorly imputed with 1000G phase 1 v.3;^44^ lung cancer used a mix between 1000G phase 1 and hase 3; glioma used a mix between 1000G phase 3, UK10K, and HRC; esophageal cancer used 1000G phase 1; and endometrial cancer used a mix between 1000G phase 3 v.5 and UK10K. For each dataset, we conducted comprehensive quality control to clean and harmonize the GWAS summary statistics across cancers. This included: (1) removing duplicate, structural, multi-allelic, and ambiguous variants; (2) confirming that strand and alleles at each variant were consistent across cancers; (3) creating a common unique marker ID; and (4) removing analytic artifacts (e.g., common variants with reported |log odds ratio| > 3). We also removed any variants with imputation quality score < 0.3 or minor allele frequency (MAF) < 0.01. After manual inspection of the results, we conducted additional *ad hoc* cleaning for individual cancer results to remove any obvious technical artifacts.

### Genetic correlations due to sample overlap

We estimated the number of controls overlapping between pairs of cancers, as these would induce a correlation in the GWAS summary statistics between cancers. We identified participating studies and any publicly available datasets (e.g., Wellcome Trust Case Control Consortium) to calculate the maximum number of controls overlapping between any two cancers. We also employed the tetrachoric correlation between binary-transformed GWAS summary *Z* scores to determine putative sample overlap.^50^^,^^51^ To avoid induced correlations due to a shared polygenic architecture, we removed all cancer-specific variants with association p < 0.1. We observed six pairs of cancers that had correlations > 0.05, and these all reflected previously known documented relationships where controls were shared between groups (Table S1). Pairs with correlations > 0.05 included breast and endometrial (0.08), ER-positive breast and endometrial (0.06), breast and ovarian (0.05), esophageal and melanoma (0.08), lung and head/neck (0.07), and lung and renal cancer (0.07).

### Local genetic correlation estimation

To identify regions in the genome with local genetic correlations between pairs of cancers, we used ρHESS^52^ (Heritability Estimation using Summary Statistics), which first estimates the local SNP-heritability for each cancer within each region based on summary statistics^53^ and then quantifies the covariance and correlation between pairs of cancers. Based on the LD pattern in 1000G European ancestry populations,^34^ ρHESS partitions the genome into 1,703 approximatively independent LD blocks. We took sample overlap between pairs of cancers into account as described above. Pairwise local genetic correlations were considered statistically significant if the p value < 0.05/1,703 = 2.94 × 10^−5^.

### Searching for independent signals shared across cancers

**Figure 1:**
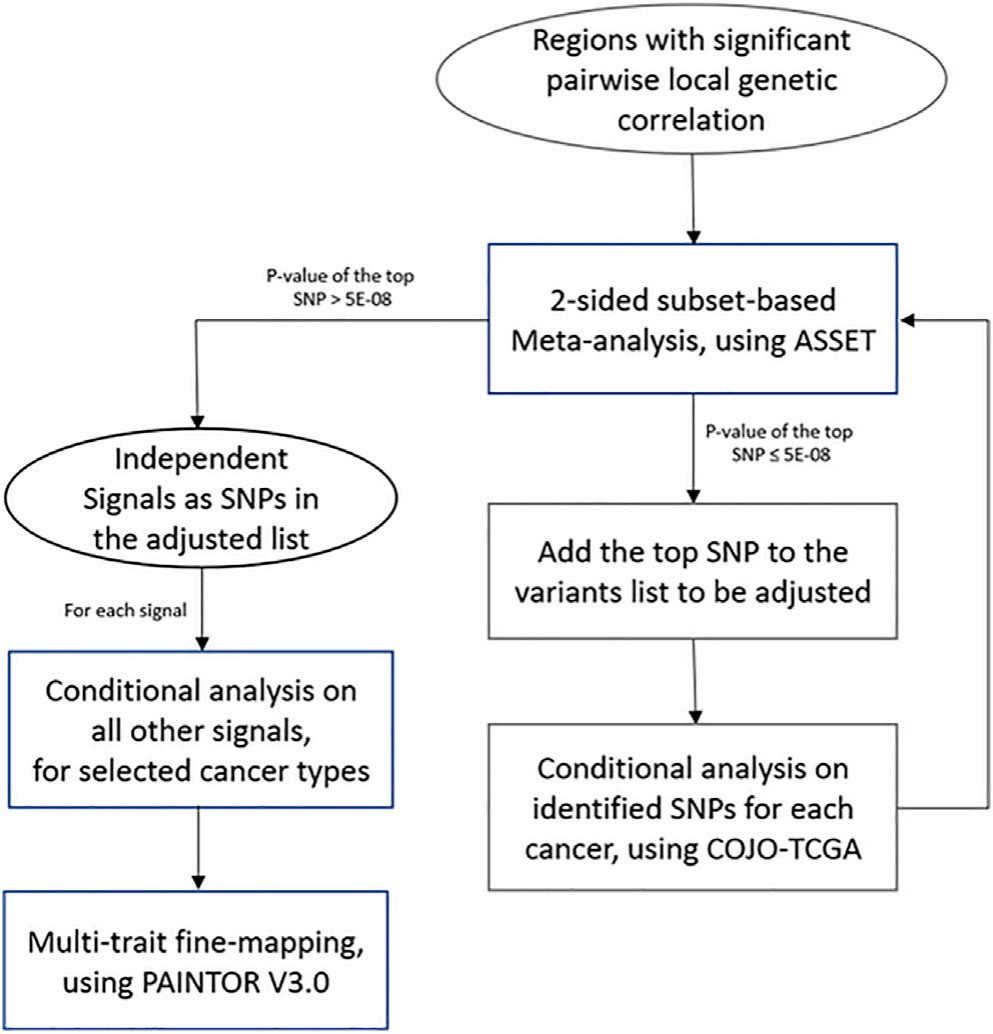
Analytical pipeline for the study Regions with significant pairwise local genetic correlation were first identified by ρHESS. For regions harboring disproportionally high shared heritability across cancers, joint test of ASSET two-sided meta-analysis and COJO conditional analysis was then repeatedly conducted to identify independent signals, until no variant reached genome-wide significance (p < 5 × 10^−8^) in two-sided ASSET meta-analysis. For each signal, GWAS summary statistics conditional on other signals of selected cancer were used in multi-trait fine-mapping to estimate the posterior probability of being causal.

Based on the local genetic correlation results, we identified a 1.2 Mb region at 5p15.33 (hg19 coordinates: 82,252–2,132,442 bp) harboring significant local heritability for multiple cancer pairs (see Results). We selected eight types of cancers (ER-negative breast, colorectal, glioma, lung, melanoma, ovarian, pancreatic, prostate) that had genome-wide significant associations in the region and showed evidence of pairwise genetic correlation (p < 0.05) with at least one other cancer having genome-wide significant associations in this region, which includes the *TERT* and *CLPTM1L* genes. We first performed a conditional analysis using COJO-GCTA^36^ on each individual cancer adjusting for the variant with the smallest p value, until no variant had a conditional p < 5 × 10^−8^. We then performed pairwise colocalization analyses using COLOC^54^ to assess if any cancers shared causal variants, after controlling for the independent signals identified in analysis of individual cancers. To comprehensively enumerate the independent cross-cancer signals within this locus, we then used an agnostic subset-based meta-analysis (ASSET)^35^ to identify variants with the strongest cross-cancer associations in this region (Figure 1). ASSET allows for opposite direction of effects across traits when assessing the association between variants and multiple traits, as implemented in the “two-sided” option in ASSET. Overlap in controls between GWASs was addressed by using the tetrachoric correlation, as described above. To determine the number of independent signals within a region, we reran all individual cancer GWASs conditioning on the top variant identified by ASSET using COJO-GCTA. The conditional analysis may be subject to the mismatch of LD between the reference panel and the population that generated the GWAS results. Consequently, we created a LD reference panel for all cancer-specific conditional analyses using European ancestry breast cancer controls (n = 40,401),^38^ which was the largest population with genotype data available. After generating updated cancer-specific GWAS summary statistics conditioned on the most significant variant (top variant), we reran the two-sided ASSET meta-analysis to identify any additional significant cross-cancer signals. We then added the new top variant from the ASSET analysis to the list of lead SNPs and reran all cancer GWASs conditioning on all lead variants using COJO-GCTA. We iteratively ran cancer-specific analyses conditioning on the identified top variants using COJO-GCTA and ran two-sided ASSET on the resulting cancer-specific association results. We repeated this procedure until no variant reached genome-wide significance in the two-sided ASSET meta-analysis. The lead variants that resulted from the two-sided ASSET meta-analyses based on the conditional cancer-specific results were regarded as candidate variants that independently affect the risk of multiple cancers. Using this approach, we identified a total of ten independent signals within the 5p15.33 region.

### Multi-trait fine-mapping

For each of the ten cross-cancer signals in the 5p15.33 region identified by our ASSET-COJO analysis, we created new cancer-specific GWAS summary statistics adjusting for the other nine top variants and estimated variant-specific posterior probabilities of causality using PAINTOR v.3.0.^37^ We varied the set of cancers included in the fine-mapping analyses of each of the ten independent cross-cancer signals as we hypothesized that not all cancers would share the same causal variant for each independent signal, but, rather, different combinations of cancers contributed to each of the ten independent signals. This was also supported by the ASSET analyses, where not all cancers contributed to the top signal for each of the ten conditional meta-analyses. In particular, ASSET provides the subset of traits that contribute to the smallest variant-specific meta-analysis p value. For each variant, two subsets are reported, with the first including traits with a positive association and the second including traits with a negative association. For each of the ten independent signals, we included a specific cancer in the PAINTOR fine-mapping analysis if: (1) it was one of the cancers selected by ASSET as a contributing phenotype in the corresponding two-sided ASSET analysis of the lead variant, or (2) the lead variant showed genome-wide significant association for that cancer in the unadjusted cancer-specific GWAS. For each independent signal, only SNPs with data for all relevant cancers were included. We ran PAINTOR under the assumption that there was only one causal variant underlying that signal. We used the same LD reference panel for the fine-mapping analysis as we did for the conditional analysis. In our primary analyses, we performed the fine-mapping with no functional annotation implemented. Since regulation of *TERT* and *CLPTM1L* expression has been linked to open chromatin conformation in previous analyses,^55^^,^^56^ we conducted a secondary analysis incorporating tissue-specific open chromatin annotations as functional prior. We obtained open chromatin narrow peaks identified from normal tissue or primary cell lines of the relevant organs of each signal, based on the ENCODE project.^57^ By overlapping variants with open chromatin peaks, we generated a binary matrix for the region, which was then implemented as the functional prior in the fine-mapping analysis.

## Results

### Local genetic correlation revealed specific regions in the genome with shared heritability across cancers

**Figure 2:**
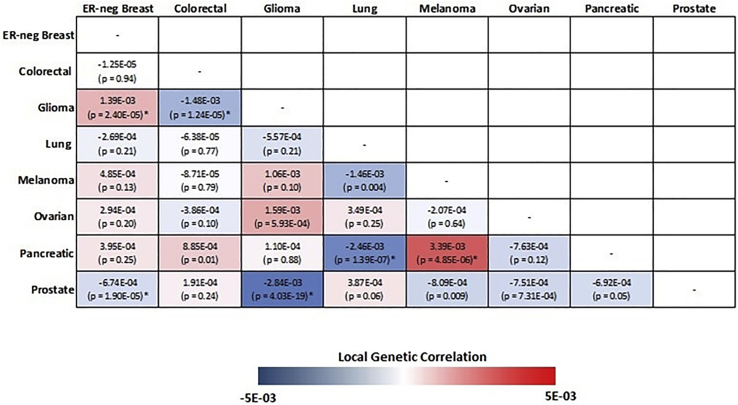
Pairwise local genetic correlation between selected cancer types at chromosome 5p15.33 (982,252–2,132,442 bp) Cancer pairs with statistically significant (p value < 0.05/1,703 = 2.94 × 10^−5^) local genetic correlation are annotated with an asterisk.

We first partitioned the genome into 1,703 regions and estimated the pairwise local genetic correlation between fourteen types of cancers. After adjusting for multiple comparisons (p value < 0.05/1,703 = 2.94 × 10^−5^), we identified thirteen pairs of cancers with statistically significant local genetic correlation across eight distinct genomic regions (Table 2). Among these, seven cancer pairs had positive genetic correlation (4q24: colorectal and prostate; 5p15.33: ER-negative breast and glioma, melanoma and pancreatic; 5q11.2: overall breast and colorectal; 8q24: colorectal and prostate; 17q12: endometrial and prostate; 19p13.11: ER-negative breast and ovarian), while six others showed negative genetic correlations (1q32: ER-negative breast and prostate; 5p15.33: glioma and prostate, colorectal and glioma, ER-negative breast and prostate, lung and pancreatic; 10q26.13: ER-positive breast and prostate). The local genetic correlation results mirrored previous observations, in that genome-wide significant variants for the identified regions have been previously reported for the individual cancers. For example, colorectal and prostate cancer showed significant local genetic correlation on chromosome 8 (126,410,917–128,659,111 bp), overlapping the 8q24.21 region, which harbors susceptibility variants for more than ten types of cancers. Similarly, a region on chromosome 19 (16,374,416–18,409,862 bp) showed significant local genetic correlation between ovarian and ER-negative breast cancer, both of which have genome-wide significant susceptibility variants in this region. One region on chromosome 5 (982,252–2,132,442 bp), harboring the *TERT* and *CLPTM1L* genes, showed significant local genetic correlation across six pairs of cancers, including ER-negative breast, colorectal, glioma, lung, melanoma, pancreatic, and prostate cancer (Figure 2). Interestingly, the direction of the genetic correlations varied between cancer pairs. For example, glioma showed significant but opposite local genetic correlations with ER-negative breast (r_g_ = 0.0014, p = 2.40 × 10^−5^) and colorectal cancer (r_g_ = −0.0015, p = 1.24 × 10^−5^). Similarly, pancreatic cancer had a positive local genetic correlation with melanoma (r_g_ = 0.0034, p = 4.85 × 10^−6^) but a negative genetic correlation with lung cancer (r_g_ = −0.0025, p = 1.39 × 10^−57^).

**Table 2:** Genomic regions with statistically significant local genetic correlations between cancers

Cancer site 1	Cancer site 2	Region	Region start	Region end	No. of SNPs	Direction	p value^a^
ER-negative breast	prostate	1q32	203334734	204681068	2,364	negative	3.45E−06
Colorectal	prostate	4q24	105305294	107501305	2,986	positive	1.05E−05
Glioma	prostate	5p15.33	982252	2132442	2,631	negative	4.03E−19
Colorectal	glioma	5p15.33	982252	2132442	2,465	negative	1.24E−05
ER-negative breast	prostate	5p15.33	982252	2132442	3,111	negative	1.90E−05
ER-negative breast	glioma	5p15.33	982252	2132442	2,631	positive	2.40E−05
Melanoma	pancreatic	5p15.33	982252	2132442	2,849	positive	4.85E−06
Lung	pancreatic	5p15.33	982252	2132442	2,935	negative	1.39E−07
Overall breast	colorectal	5q11.2	55417349	56621102	2,131	positive	1.97E−05
Colorectal	prostate	8q24	126410917	128659111	4,275	positive	1.97E−16
ER-positive breast	prostate	10q26.13	123231465	123900545	1,481	negative	1.22E−06
Endometrial	prostate	17q12	34469036	36809344	2,748	positive	5.01E−09
ER-negative breast	ovarian	19p13.11	16374416	18409862	4,103	positive	1.11E−07

Local genetic correlation between cancers across the genome (N = 1,703 regions) was estimated using *HESS*.

a Cutoff of the statistical significance was defined as p < 0.05/1,703 = 2.94E−05, after adjusting for multiple comparison.

### Distinct patterns of regional GWAS association p values for the variants at 5p15.33

**Figure 3:**
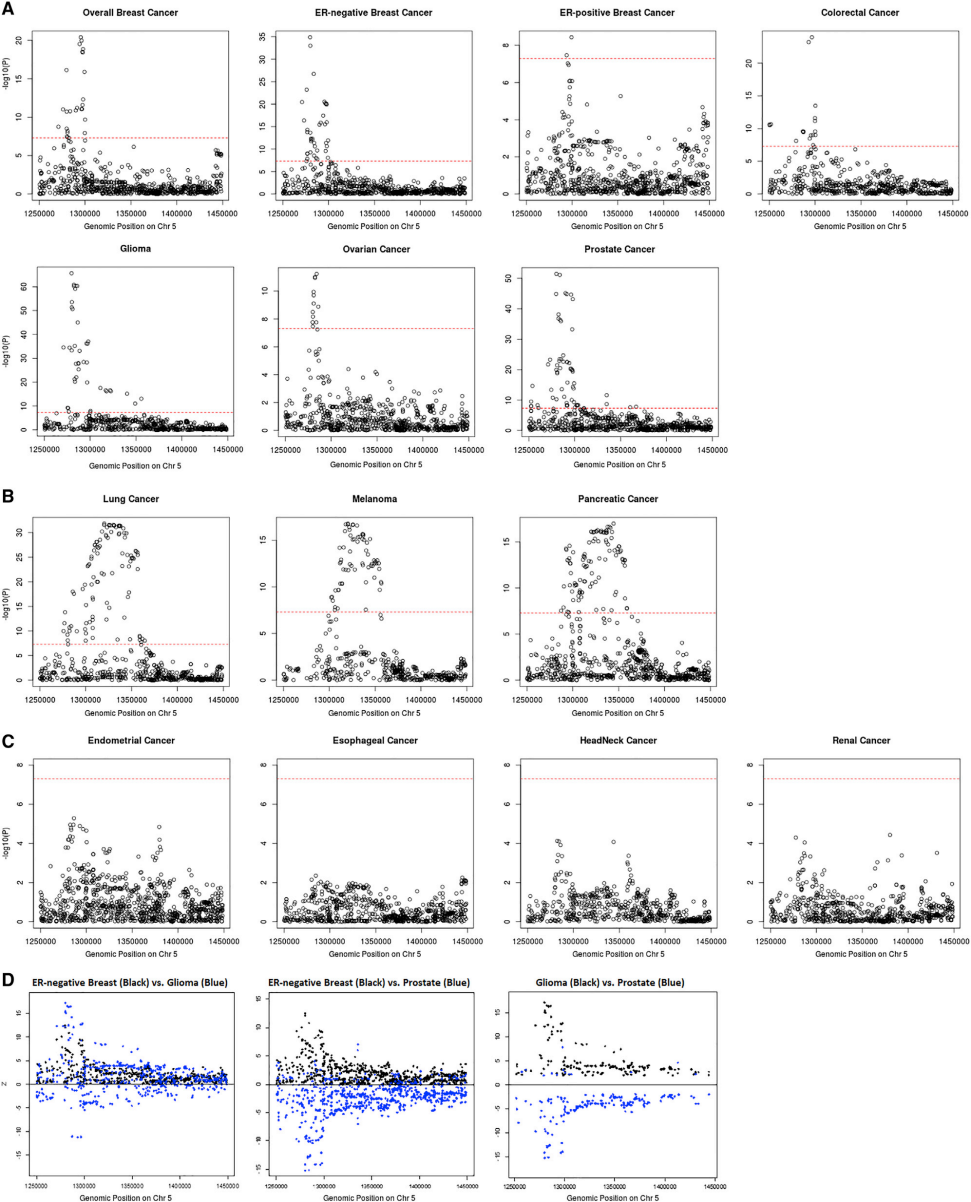
Categorizing 14 cancer types into three tiers based on their p value distribution at 5p15.33 Pattern A cancers (A) have one single peak by the *TERT* gene; pattern B cancers (B) have a broader signal at the *CLPTM1L* gene as well as a signal by the *TERT* gene; pattern C cancers (C) have no genome-wide significant association in this region. Genome-wide significant levels at p value = 5 × 10^−8^ are marked with red dashed line in (A)–(C). Distribution of *Z* scores at the 5p15.33 region from the GWAS results of ER-negative breast, glioma, and prostate cancer (D). Only variants with p < 0.05 for both cancers are included. While the associations for ER-negative breast cancer and glioma overlap, the SNP associations with ER-negative breast cancer and prostate, as well as glioma and prostate, are in opposite directions.

Based on the local genetic correlation results, the 5p15.33 region may harbor key genetic variants related to multiple cancer types. Indeed, multiple susceptibility variants in this region have been reported for at least ten cancer types, including ER-negative breast, colorectal, glioma, lung, melanoma, ovarian, pancreatic, and prostate cancer. To obtain a more complete understanding of the association patterns in this region, we created cancer-specific regional association plots for 5p15.33 (Figure 3A). We observed three different patterns of association. Pattern A, which includes breast (overall, ER-positive, and ER-negative), colorectal, glioma, ovarian, and prostate cancer, displayed one sharp genome-wide significant signal in a narrow region (~30 kb) overlapping the *TERT* gene (chr5: 1,253,282–1,295,178 bp). Pattern B, which includes lung, melanoma, and pancreatic cancer, has a broader genome-wide significant signal overlapping both the *TERT* (chr5: 1,253,282–1,295,178 bp) and *CLPTM1L* genes (chr5: 1,317,869–1,345,180 bp) (Figure 3B). Pattern C, which includes endometrial, esophageal, head/neck, and renal cancer, did not have a genome-wide significant signal in this region (Figure 3C). Interestingly, the distribution of variant-specific associations for some cancers was highly similar but in the opposite direction (Figure 3D), suggesting that GWAS associations discovered in this region may underly tissue-specific regulations across cancers. The association-based classification of cancers was highly consistent with our local genetic correlation results. All cancer types showing shared significant local genetic correlation in this region were in either pattern A or B, and thus we excluded the cancers belonging to pattern C for further analyses. For breast cancer, we limited our analysis to ER-negative breast cancer, as it had the strongest association at 5p15.33. Along with colorectal, glioma, lung, melanoma, ovarian, pancreatic, and prostate cancer, a total number of eight cancer types were used in the fine-mapping cross-cancer analyses.

### Ten independent signals were identified based on multi-cancer meta-analysis results

Given the important biological function of the *TERT* and *CLPTM1L* genes, previous cancer fine-mapping efforts in this region, and the appearance of multiple association peaks for some of the cancers, it is plausible to assume that multiple variants in this region affect cancer risk independently. To test this assumption, we performed a conditional analysis using COJO-GCTA for each cancer to enumerate the independent signals at the 5p15.33 region. Six of the eight cancers of interest, including ER-negative breast, colorectal, glioma, lung, pancreatic, and prostate, were identified with two or more independent variants (Table S2). A total number of thirteen variants were identified, of which four were shared by two cancer types. By using conditional analysis results of each cancer, we then assessed the probability of two cancers sharing a single causal variant using a Bayesian-based colocalization approach.^54^ Glioma and melanoma were estimated to be likely sharing a causal variant (posterior probability [PP] = 0.519; Table S3), even after controlling for the effect of identified signals of individual cancers. These results suggest that multiple independent cross-cancer signals may exist in this region.

However, current state-of-the-art statistical fine-mapping tools often struggle to make inference of causality under the assumption of multiple causal variants. Further, it is likely that not all cancers share all causal variants. To get an estimate of the number of independent association signals across cancers in this region, we conducted iterative meta-analyses using individual cancer-specific association results from conditional analysis as generated by COJO-GCTA (see Material and methods). We adopted the two-sided analysis scheme in ASSET to allow for the detection of effects in opposite directions.

**Figure 4:**
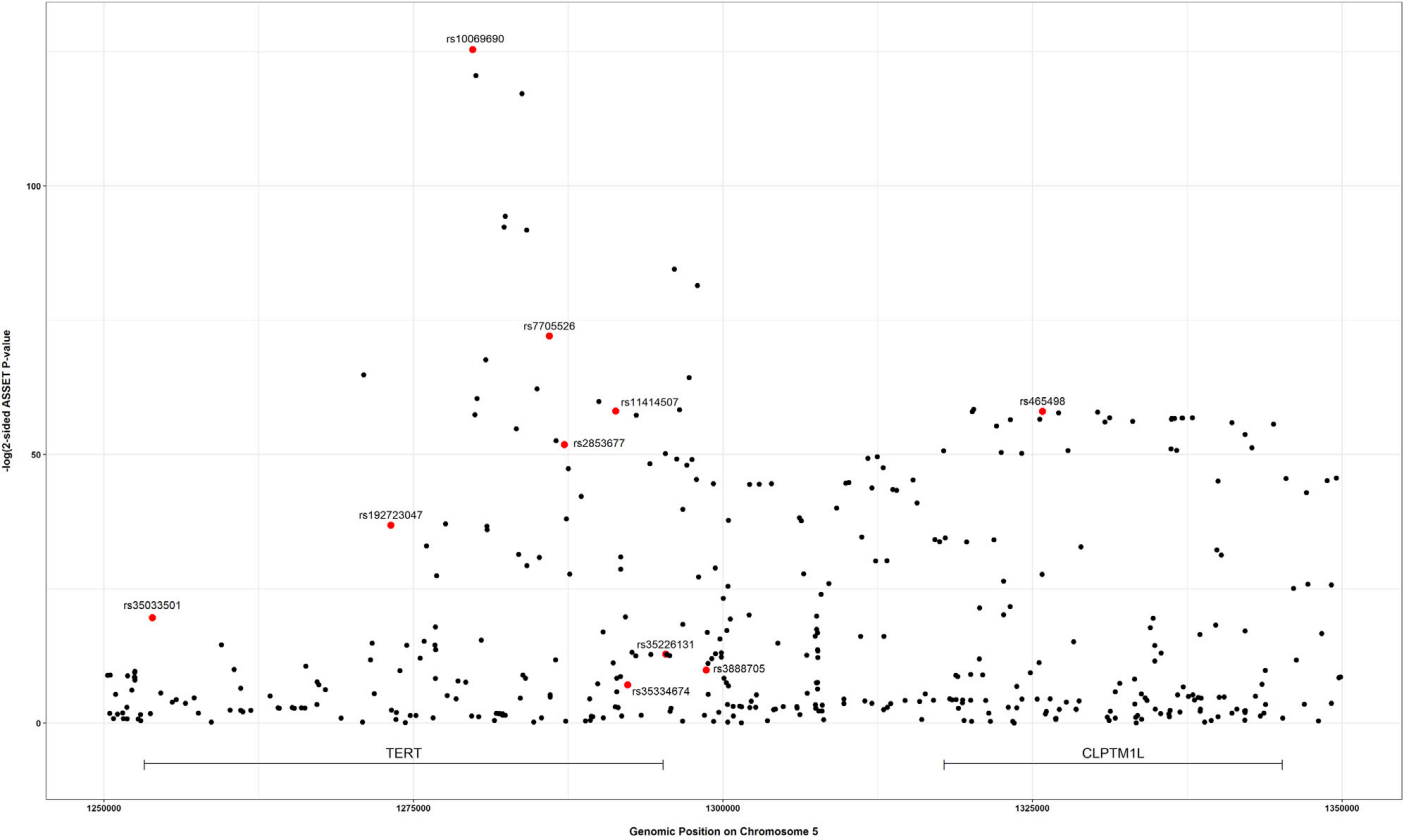
Distribution of two-sided subset-based meta-analysis p values across eight cancer types at the 5p15.33 region Index variants of ten independent candidate signals, identified by the iterative COJO-ASSET analysis, are annotated and marked in red.

**Figure 5:**
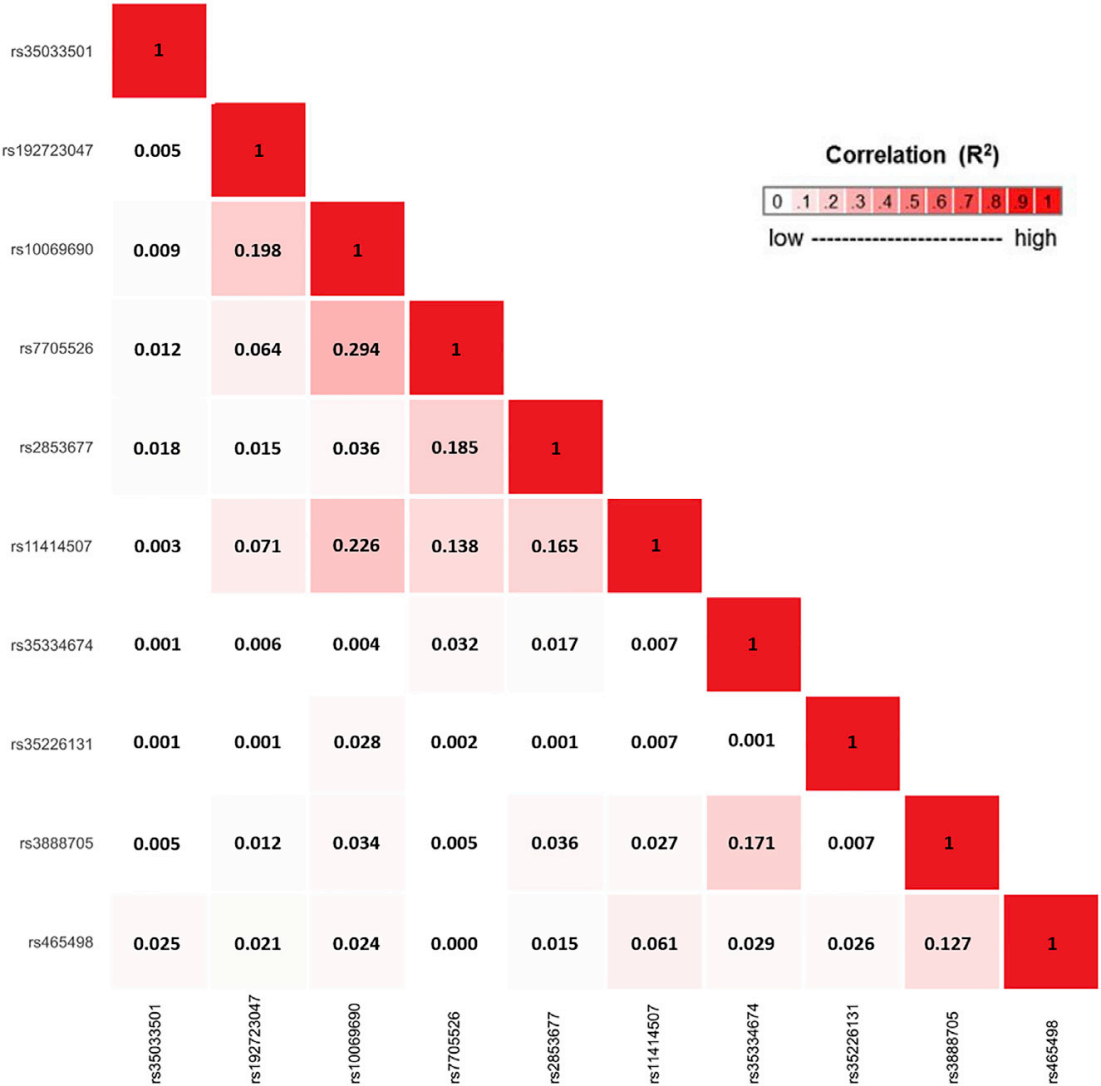
Correlation matrix showing the pairwise linkage disequilibrium (LD) between 10 candidate signals, identified using an iterative COJO-ASSET analysis LD was calculated based on the European ancestry populations in 1000 Genomes (1000G) Project.

The strongest associated variant in the two-sided ASSET meta-analysis was rs10069690 (chr5: 1,279,790, p = 4.05 × 10^−126^; Figure 4), which was positively associated with ER-negative breast cancer and glioma while negatively associated with pancreatic and prostate cancer. We adjusted the cancer-specific GWAS results for rs10069690 using COJO-GCTA, and then reran the two-sided ASSET meta-analysis with the rs10069690-adjusted cancer-specific results. We observed the strongest association for rs465498 (chr5: 1,325,803, p = 1.75 × 10^−59^), which was positively associated with melanoma and pancreatic cancer and negatively associated with lung cancer. We added rs465498 to the set of variants to be conditioned on in the cancer-specific GWASs and iterated this process until no variant reached genome-wide significance (p < 5 × 10^−8^) in two-sided ASSET meta-analysis. In the end, we obtained ten conditionally independent significant SNPs (Table 3; Figure S1). The pairwise r^2^ between the ten SNPs ranged between 0.001 and 0.294 as based on 1000G European ancestry data,^58^ which indicated that the pairwise correlations between the identified signals were weak (Figure 5).

**Table 3:** Ten independent cross-cancer signals in 5p15.33 region identified in the joint analysis of COJO-ASSET

No. of iteration	SNP with top ASSET p value	ASSET p value^a^	Significant cancer subset, identified by ASSET^b^	GWAS p values^c^							
				ER-neg BrCa	Colorectal	Glioma	Lung	Melanoma	Ovarian	Pancreatic	Prostate
Initiation	rs10069690 (5:1279790:C:T)	4.05E−126	set 1: ER-neg BrCa, glioma; set 2: pancreatic, prostate	1.34E−35∗	1.13E−01	2.32E−66∗	9.39E−01	9.08E−01	1.74E−08∗	3.29E−03	1.44E−45∗
1	rs465498 (5:1325803:A:G)	1.75E−59	set 1: melanoma, pancreatic; set 2: lung	1.61E−02	3.89E−02	6.85E−05	2.68E−32∗	2.08E−17∗	1.49E−01	7.45E−17∗	9.15E−05
2	rs2853677 (5:1287194:G:A)	3.24E−39	set 1: ER-neg BrCa, colorectal, lung; set 2: glioma, melanoma, ovarian, pancreatic, prostate	4.94E−05	3.65E−10∗	1.08E−28∗	2.66E−18∗	1.12E−02	1.49E−06	2.87E−08∗	1.53E−02
3	rs11414507 (5:1291331:A:AC)	1.29E−19	set 1: ER-neg BrCa; set 2: prostate	1.34E−16∗	NA	NA	NA	NA	1.63E−04	8.18E−01	1.61E−45∗
4	rs35033501 (5:1253918:C:T)	9.18E−15	set 1: lung, melanoma, prostate; set 2: ER-neg BrCa, pancreatic	9.90E−05	8.55E−03	1.24E−02	3.84E−01	3.66E−02	5.55E−01	4.48E−05	2.37E−15∗
5	rs7705526 (5:1285974:C:A)	1.42E−11	set 1: glioma, lung, melanoma; set 2: ER-neg BrCa, colorectal, pancreatic, prostate	1.37E−04	3.17E−04	5.01E−61∗	1.01E−18∗	3.24E−03	1.34E−09∗	2.15E−03	2.78E−14∗
6	rs192723047 (5:1273183:A:G)	1.63E−11	set 1: prostate; set 2: ER-neg BrCa	4.37E−17∗	NA	NA	NA	NA	1.21E−02	8.96E−02	5.40E−24∗
7	rs35226131 (5:1295373:C:T)	2.32E−09	set 1: pancreatic; set2: colorectal, glioma, prostate	8.83E−02	5.17E−07	8.66E−01	NA	NA	3.63E−01	4.30E−08∗	3.20E−06
8	rs35334674 (5:1292299:G:A)	1.24E−08	set 1: ER-neg BrCa, pancreatic; set 2: colorectal, glioma, lung, prostate	3.36E−02	4.40E−07	4.07E−02	2.15E−02	NA	5.93E−01	1.43E−02	3.23E−02
9	rs3888705 (5:1298645:G:A)	2.65E−08	set 1: ER-neg BrCa, colorectal, ovarian; set 2: pancreatic, prostate	1.78E−02	1.04E−03	9.32E−07	3.62E−03	NA	2.75E−03	7.13E−01	1.94E−03
10	rs148487301 (5:1318797:T:C)	8.70E−06	not reached genome-wide significance, iteration stopped								

∗Genome-wide significance with p value < 5 × 10^−8^. BrCa, breast cancer; NA, the SNP was not included in the GWAS results of corresponding cancer..

a p values from the ASSET meta-analysis allowing opposite direction of the effect (two-sided analysis).

b Cancer subsets included in the two-sided ASSET meta-analysis with best p value. Set 1/2 represents the selected cancer types with positive/negative association with the SNP.

c p values from the original GWAS results of eight cancers.

For each of the ten independent signals, the number of cancer types contributing to the association as identified by ASSET ranged from two to eight. Although SNPs rs10069690 and rs7705526 were both genome-wide significant variants for ovarian cancer (p = 1.74 × 10^−8^ and 1.34 × 10^−9^, respectively), ASSET did not include ovarian cancer as a contributing cancer to the meta-analysis results for either of the SNPs. To ensure that we included all relevant cancers in the fine-mapping analysis of each independent signal, we extracted the original cancer GWAS results for the ten independent SNPs and manually added any cancers to the list of contributing cancers if that cancer showed a genome-wide significant association with a specific SNP but was not included on the list of traits optimizing the ASSET meta-analysis. For each of the ten SNPs, we then applied COJO-GCTA on each included cancer GWAS dataset to obtain cancer-specific results conditioned on the other nine lead SNPs and used these adjusted summary statistics in the fine-mapping analyses.

### Cross-cancer fine-mapping proposes candidate causal variants shared by cancers

To identify candidate causal SNPs within the ten independent signals identified in the conditional analyses, we conducted a multi-cancer fine-mapping analysis using PAINTOR for each signal. We first performed fine-mapping analyses with no functional annotation data implemented. For the ten candidate signals, the size of credible sets comprising a cumulative 95% PP of causality ranged from one to fifteen variants (Table 4; Figure S2). All SNPs identified as lead SNPs in the conditional analysis were included in the 95% PP credible set of the corresponding fine-mapping analysis, with six of them having the highest PP in its set (rs35033501: PP = 0.875; rs35334674: PP = 0.987; rs192723047, rs10069690, rs7705526, rs2853677: PP > 0.999). The fine-mapping analysis based on the signal identified by SNP rs35226131 included data on colorectal, glioma, pancreatic, and prostate cancer. Although rs35226131 was identified as the SNP with the highest PP of being causal, the PP was relatively modest (PP = 0.273) and comparable to nearby SNPs (rs35161420, PP = 0.239; rs61748181, PP = 0.228). Fine-mapping analysis of the signals indexed by rs11414507 (ER-negative breast and prostate cancer) and rs465498 (lung, melanoma, and pancreatic cancer) both identified a SNP located ~5 kb away from the original lead SNP, with the highest PPs for rs7712562 (PP = 0.367) and rs380286 (PP = 0.462), respectively. Fine-mapping analysis of rs3888705 (ER-negative breast, colorectal, ovarian, pancreatic, and prostate cancer) identified a credible set consisting of 15 variants with PPs ranging between 0.01 and 0.10, with the lead SNP rs3888705 having a PP of 0.092.

**Table 4:** Statistical fine-mapping prioritized the potential causal SNP within 10 independent cross-cancer signals in 5p15.33 region, using PAINTOR v.3.0

Index SNP	Fine-mapped cancer types	Fine-mapping without functional prior				Fine-mapping with functional prior^a^			
		95% PP credible set^b^	PP, index SNP	SNP with highest PP	Highest PP	95% PP credible set	PP, index SNP	SNP with highest PP	Highest PP
rs35033501 (5:1253918:C:T)	ER-neg BrCa, lung, melanoma, pancreatic, prostate	rs35033501, rs71595003	0.875	rs35033501 (5:1253918:C:T)	0.875	rs71595003	<0.001	rs71595003 (5:1292118:G:A)	0.999
rs192723047 (5:1273183:A:G)	ER-neg BrCa, prostate	rs192723047	1.000	rs192723047 (5:1273183:A:G)	1.000	rs192723047	1.000	rs192723047 (5:1273183:A:G)	1.000
rs10069690 (5:1279790:C:T)	ER-neg BrCa, glioma, ovarian, pancreatic, prostate	rs10069690	1.000	rs10069690 (5:1279790:C:T)	1.000	rs10069690	1.000	rs10069690 (5:1279790:C:T)	1.000
rs7705526 (5:1285974:C:A)	ER-neg BrCa, colorectal, glioma, lung, melanoma, ovarian, pancreatic, prostate	rs7705526	1.000	rs7705526 (5:1285974:C:A)	1.000	rs7705526	1.000	rs7705526 (5:1285974:C:A)	1.000
rs2853677 (5:1287194:G:A)	ER-neg BrCa, colorectal, glioma, lung, melanoma, ovarian, pancreatic, prostate	rs2853677	1.000	rs2853677 (5:1287194:G:A)	1.000	rs2853677	1.000	rs2853677 (5:1287194:G:A)	1.000
rs11414507 (5:1291331:A:AC)	ER-neg BrCa, prostate	rs7712562, rs74682426, rs11414507, rs7449190	0.265	rs7712562 (5:1296072:A:G)	0.367	rs7712562, rs11414507	0.419	rs7712562 (5:1296072:A:G)	0.581
rs35334674 (5:1292299:G:A)	ER-neg BrCa, colorectal, glioma, lung, pancreatic, prostate	rs35334674	0.987	rs35334674 (5:1292299:G:A)	0.987	rs35334674	0.988	rs35334674 (5:1292299:G:A)	0.988
rs35226131 (5:1295373:C:T)	colorectal, glioma, pancreatic, prostate	rs35226131, rs35161420, rs61748181, rs33958877, rs114616103	0.273	rs35226131 (5:1295373:C:T)	0.273	rs35226131, rs35161420, rs61748181, rs33958877, rs114616103	0.273	rs35226131 (5:1295373:C:T)	0.273
rs3888705 (5:1298645:G:A)	ER-neg BrCa, colorectal, ovarian, pancreatic, prostate	rs34156553, rs4075202, rs3888705, rs77776598, rs4975539, rs6875445, rs4583925, rs78844046, rs79323805, rs4507531, rs78368589, rs4487533, rs6554678, rs4498293, rs4532396	0.092	rs34156553 (5:1243245:C:T)	0.103	rs34156553, rs4075202, rs3888705, rs77776598, rs4975539, rs6875445, rs4583925, rs78844046, rs79323805, rs4507531, rs78368589, rs4487533, rs6554678, rs4498293, rs4532396	0.092	rs34156553 (5:1243245:C:T)	0.103
rs465498 (5:1325803:A:G)	lung, melanoma, pancreatic	rs380286, rs421629, rs465498, rs452932, rs459961, rs455433, rs13178866, rs460073	0.146	rs380286 (5:1320247:G:A)	0.462	rs421629, rs465498	0.437	rs421629 (5:1320136:G:A)	0.563

a Used open chromatin narrow peaks identified from the normal tissue or primary cells of the disease-related organs as the functional prior. Open chromatin narrow peaks were obtained from the ENCODE project.

b SNPs within the credible set were ranked by the posterior probability (PP).

**Figure 6:**
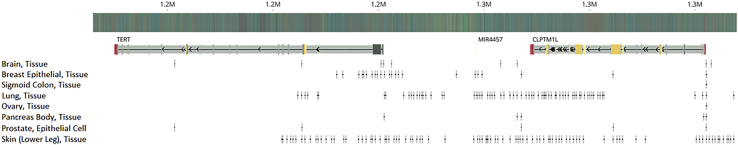
Open chromatin in different cancer types Genomic location of tissue-specific open chromatin narrow peaks, which were used as functional prior in the fine-mapping analysis.

To assess the impact of adding *a priori* information on functional importance, we downloaded tissue-specific open chromatin narrow peaks of normal tissues or primary cell lines for the relevant organs for each signal from the ENCODE project (Figure 6; Table S4). By overlapping the functional annotations with the variants of interest, we repeated the fine-mapping analysis for all the candidate signals (Table 4; Figure S2). Seven of the ten candidate signals showed consistent 95% PP credible sets as the previous fine-mapping analyses without functional annotations. However, fine-mapping analysis of rs35033501 (ER-negative breast, lung, melanoma, pancreatic, and prostate cancer) prioritized rs71595003, residing in an open chromatin peak for breast epithelial tissue, with a PP of 0.999. In contrast, rs35033501, which had a PP of 0.875 in the analysis without annotations, had a PP < 0.001 when information about open chromatin was added. For the fine-mapping analyses of rs11414507 (ER-negative breast, prostate), the size of the 95% PP credible set shrank from four to two, which included the index SNP rs11414507 (PP = 0.42) as well as rs7712562 (PP = 0.58). Both rs11414507 and rs7712562 were located in open chromatin peaks in breast epithelial tissue. Similarly, after we implemented the functional annotation data, only two SNPs were included in the 95% PP credible set of the signal indexed by rs465498 (lung, melanoma, pancreatic), compared to eight SNPs in the analysis without functional information. The index SNP rs465498 had a comparable PP (0.437) as rs421629 (PP = 0.563), and both SNPs were located within open chromatin peaks in lung tissue.

## Discussion

In this study, we leveraged GWAS summary statistics from 14 cancer types to estimate local genetic correlations and conduct follow-up fine-mapping of shared cancer regions in the genome. By partitioning the genome into independent blocks as defined by LD, we comprehensively estimated pairwise local genetic correlations between the included cancers. We identified 13 cancer pairs with significant local genetic correlation across eight distinct genomic regions. Among these, one region on chromosome 5p15.33 harboring the *TERT* and *CLPTM1L* genes had statistically significant shared heritability for seven cancer types, including ER-negative breast, colorectal, glioma, lung, melanoma, pancreatic, and prostate cancer. By utilizing an iterative analysis, we identified ten independent cross-cancer SNP signals within this locus. We then conducted fine-mapping analyses for each independent signal and generated 95% posterior probability credible sets both without and with *a priori* functional information.

Our pairwise local genetic correlation results were highly consistent with the conclusions of previous GWASs and cross-cancer analyses. The pleiotropic effect of variants in the 8q24 region between multiple types of cancer, including colorectal and prostate cancer, has been previously demonstrated and replicated in studies across populations of different ancestries.^12^^,^^59^^,^^60^ The 5p15.33 region, containing the *TERT* and *CLPTM1L* genes, has also been associated with multiple cancers.^17^, ^18^, ^19^, ^20^, ^21^, ^22^, ^23^ Other significant genomic regions identified in our study, including 1q32.1 (ER-negative breast and prostate), 4q24 (colorectal and prostate), 5q11.2 (overall breast and colorectal), 10q26.13 (ER-positive breast and prostate), 17q12 (endometrial and prostate), and 19p13.11 (ER-negative breast and ovarian), have also been identified as pleiotropic loci in previous analyses.^61^^,^^62^ Previous efforts have been devoted to identifying pleiotropic variants, by using either a subset-based meta-analysis approach^61^ or categorizing genome-wide significant loci of multiple cancers by LD patterns.^62^ Our analysis complements these, as we aggregated the per-SNP effect within the loci, estimated the local heritability of each cancer, and quantified the local genetic correlation between the cancer pairs. These “shared heritability hotspots” identified in our analysis may contain genes with strong effect on multiple cancers or harbor multiple risk variants and biological mechanisms that can independently affect the risk of different cancers. Our results can thus be utilized to prioritize candidate regions for future discoveries of causal variants and functional follow-up.

As the 5p15.33 region harboring the *TERT* and *CLPTM1L* genes was the only region that displayed more than one statistically significant pairwise genetic correlation, we focused our continued efforts on this region. The *TERT* gene encodes the catalytic subunit of telomerase reverse transcriptase,^25^ which is a crucial enzyme for maintaining telomere length. Mendelian randomization studies have shown that genetically determined telomere length is associated with the risk of multiple cancer types, including glioma, ovarian, lung, and melanoma, but is not associated with the risk of other cancers included here, such as breast and prostate.^63^, ^64^, ^65^ In our study, we observed local negative genetic correlations and opposite direction of SNP effects between specific cancer types, which indicate that genetic variation in this region is likely to affect cancer risk through multiple distinct biological pathways, of which telomere length is only one implicated mechanism. Meanwhile, the *CLPTM1L* gene encodes the cleft lip and palate-associated transmembrane-1 like protein, which has been reported to play a role in cell apoptosis and cytokinesis and is overexpressed in lung and pancreatic cancer.^66^, ^67^, ^68^ Given its important biological function and significant association with a broad set of cancers, we assumed that multiple variants in this region may independently influence the risk of various types of cancers. By iteratively conducting conditional meta-analyses, we identified ten independent signals (seven in the *TERT* gene, one in the *CLPTM1L* gene, and two between *TERT* and *CLPTM1L*). Our study results are comparable to a previous study published by Wang et al.,^56^ which conducted a subset-based meta-analysis across six types of cancers (bladder, glioma, lung, pancreatic, prostate, and testicular). Several signals identified in our study have either been proposed (rs10069690, rs2853677) or are correlated with the independent signals reported in that study (rs7705526 versus rs7726159, r^2^ = 0.87; rs465498 versus rs451360, r^2^ = 0.34). We only included cancers with genome-wide significant signals in this region into the subset-based meta-analysis and conditional analysis. Compared to the study presented by Wang et al.,^56^ our study further included several common cancers (ER-negative breast, colorectal, melanoma, and ovarian), while we did not have data on bladder and testicular cancer. With an increased number of cancers and larger sample sizes, we were able to refine the cross-cancer signals in this important region. In addition, independent signal rs465498 identified in our study was in strong correlation with two previously identified susceptible loci at the *CLPTM1L* gene, including pancreatic cancer SNP rs31490 (r^2^ = 0.96)^69^ and lung, melanoma, and prostate cancer SNP rs401681 (r^2^ = 0.96).^21^^,^^70^^,^^71^ Our findings imply that the association between the *CLPTM1L* gene and various types of cancer can be potentially attributed to one distinct signal.

When estimating the local genetic correlation across cancers, we considered subtypes for breast (ER-negative and ER-positive) and lung cancer (adenocarcinoma, small cell, and squamous cell). Despite the relatively smaller GWAS sample size (21,468 for ER-negative breast cancer compared to 122,977 for overall breast cancer), ER-negative breast cancer showed stronger associations and higher genetic correlation with other cancers in the 5p15.33 region, as compared to ER-positive and overall breast cancer. In contrast, the three subtypes of lung cancer had either no genome-wide significant hits at the 5p15.33 region (small cell) or had weaker local genetic correlation estimates (adenocarcinoma and squamous cell, data not shown) than overall lung cancer. We thus included ER-negative breast cancer and overall lung cancer in the subsequent analyses.

Multiple lead SNPs with high posterior probability have been reported to affect telomere length. SNP rs7705526 is significantly associated with telomere length in multiple populations.^72^, ^73^, ^74^, ^75^ SNP rs2853677 has been associated with relative telomere length in a breast cancer case-only cohort in Han Chinese,^76^ as well as leukocyte telomere length in a European ancestry population.^75^ SNP rs35226131 is perfectly correlated with a nonsynonymous variant (rs61748181) in *TERT*, which results in a protein-level change from alanine to threonine and negatively influences telomere length.^15^^,^^71^ SNP rs10069690 has been found to significantly interact with recent use of non-steroidal anti-inflammatory drugs (NSAIDs) to alter telomere length in a colorectal cancer case-control study.^77^ SNP rs465498, located in the *CLPTM1L* gene, has been reported to be significantly associated with telomere length among Han Chinese.^78^ We could not find previous data on the role of other five lead SNPs identified by our study, and it is thus possible that other unknown mechanisms are involved.

Since previously identified cancer risk SNPs at 5p15.33 have been linked to open chromatin conformation,^55^^,^^56^ we further included regions of open chromatin for related tissues from the ENCODE project as functional prior in our fine-mapping analysis.^57^ The results for five signals (lead SNPs rs192723047, rs10069690, rs7705526, rs2853677, and rs35334674) remained unchanged, with each having a credible set containing one single SNP with a posterior probability of 1.00. After incorporating open chromatin peaks as a prior, the 95% posterior probability credible sets became smaller for three signals (lead SNPs rs35033501, rs11414507, and rs465498), as SNPs located in open chromatin peaks obtained a higher posterior probability of being causal. For the other two signals (lead SNPs rs35226131 and rs3888705), the size of each 95% credible set was relatively large in analyses with and without functional annotations. No SNPs in these regions had a predominantly high posterior probability, nor did any of them overlap with the open chromatin peaks of any related tissue. The fine-mapping results for these two signals should thus be interpreted with extra caution.

Our study has several strengths and limitations. We used cancer GWAS summary statistics published by each collaborating consortium, which maximized our sample sizes and provided large statistical power. This is also the first study to comprehensively quantify the local genetic correlation across multiple common cancers. We innovatively adopted the joint analysis pipeline of two-sided ASSET meta-analysis and COJO-GCTA. This approach enabled us to both validate the proposed pleiotropic loci and explore novel independent signals, under the complex genetic architecture in the 5p15.33 region. It is also important to recognize some limitations. Although we chose an internal population (breast cancer controls) to generate the LD reference panel for the conditional analyses and fine-mapping, bias may still inevitably exist as the mismatch of LD between the reference and the population of other cancers. The study population was limited to European ancestry individuals only, and therefore any conclusions of our research may not be applicable to other ancestries. Including multiple ancestries would also allow for refinement of the fine-mapping signals, since LD structure varies between populations. Moreover, some of the GWASs included in the present study (e.g., breast and ovarian) shared controls. Although we accounted for this overlap in the local genetic correlation analysis and the subset-based meta-analysis, we were not able to take these into account in the fine-mapping analysis, as PAINTOR currently does not adjust for sample overlap. However, we do not believe this will have a qualitative impact on our results. Meanwhile, although our analysis included a large number of cancer types, other cancers, including bladder and testicular, which have shown genome-wide significant signals in the 5p15.33 region,^21^^,^^79^ were not included. Further, we could have missed any potentially causal variants that were not included in our analyses for various reasons (e.g., poorly imputed or rare variants). Finally, the tissue-specific open chromatin peaks used as the functional prior in our fine-mapping analysis were from adult tissue. Some of these tissues may not express much of *TERT*, and thus these annotations may not necessarily reflect a cellular context where *TERT* and the enhancers that promote *TERT* expression are active. Our fine-mapping analysis should thus be interpreted with some caution. Since the fine-mapping analysis was solely based on bioinformatic analysis, further functional validation using molecular biology experiments is required to fully understand the mechanisms at play in this region.

In summary, our study identified genomic regions with significant local genetic correlations across 14 types of common cancers. We further enumerated the independent pleiotropic signals in the 5p15.33 region and performed a cross-cancer fine-mapping for each signal, using up-do-date bioinformatics tools. Results from our study provide novel evidence of the shared inherited basis of human cancers and expand our understanding of the role played by the *TERT-CLPTM1L* region in cancer development.

## Declaration of interests

B.M.W. has received research grants from Celgene and Eli Lilly and has consulting relationship with BioLineRx, Celgene, and Grail. R.A.E. has received speaker honoraria from the GU-ASCO meeting (January 2016), RMH FR meeting (November 2017, supported by Janssen), University of Chicago invited talk (May 2018), and ESMO (September 2019, supported by Bayer & Ipsen) and served as member of external expert committee at the Prostate Dx Advisory Panel (June 2020). All other authors declare no competing interests.

## Data and code availability

All code is available from the corresponding author on request. Data availability varies by cancer site. Publicly available GWAS summary statistics are available at http://bcac.ccge.medschl.cam.ac.uk/bcacdata/%20oncoarray/oncoarray-and-combined-summary-result/ (breast), http://practical.icr.ac.uk/blog/?page_id=8164 (prostate), http://ocac.ccge.medschl.cam.ac.uk/data-projects/results-lookup-by-region (ovarian), and https://www.ebi.ac.uk/gwas/downloads/summary-statistics (endometrial). For additional data, please contact the corresponding author.

## Web resources

Breast Cancer Association Consortium, http://bcac.ccge.medschl.cam.ac.uk/bcacdata/%20oncoarray/oncoarray-and-combined-summary-result/
The Institute of Cancer Research PRACTICAL, http://practical.icr.ac.uk/blog/page_id=8088
Ovarian Cancer Association Consortium, http://ocac.ccge.medschl.cam.ac.uk/data-projects/results-lookup-by-region
GWAS Catalog, https://www.ebi.ac.uk/gwas/downloads/summary-statistics

## Supplemental information

Document S1. Tables S1–S4, Figures S1 and S2, Supplemental acknowledgments, Consortium authors

Document S2. Article plus supplemental information
