# Supplementary material for: Large-scale cross-cancer fine-mapping of the 5p15.33 region reveals multiple independent signals
Source: HGG Adv. 2021 Jun 12;2(3):100041. doi: 10.1016/j.xhgg.2021.100041 (PMC8336922; doi:10.1016/j.xhgg.2021.100041)
Supplement: Document S1. Tables S1–S4, Figures S1 and S2, Supplemental acknowledgments, Consortium authors [file mmc1.pdf]

HGGA, Volume *2*

Supplemental information

Large-scale cross-cancer fine-mapping of the 5p15.33 region reveals multiple independent signals

Hongjie Chen, Arunabha Majumdar, Lu Wang, Siddhartha Kar, Kevin M. Brown, Helian Feng, Constance Turman, Joe Dennis, Douglas Easton, Kyriaki Michailidou, Jacques Simard, Breast Cancer Association Consortium (BCAC), Timothy Bishop, Iona C. Cheng, Jeroen R. Huyghe, Stephanie L. Schmit, Colorectal Transdisciplinary Study (CORECT), Colon Cancer Family Registry Study (CCFR), Genetics and Epidemiology of Colorectal Cancer Consortium (GECCO), Tracy A. O'Mara, Amanda B. Spurdle, Endometrial Cancer Association Consortium (ECAC), Puya Gharahkhani, Johannes Schumacher, Janusz Jankowski, Ines Gockel, Esophageal Cancer GWAS Consortium, Melissa L. Bondy, Richard S. Houlston, Robert B. Jenkins, Beatrice Melin, Glioma International Case Control Consortium (GICC), Corina Lesseur, Andy R. Ness, Brenda Diergaarde, Andrew F. Olshan, Head-Neck Cancer GWAS Consortium, Christopher I. Amos, David C. Christiani, Maria T. Landi, James D. McKay, International Lung Cancer Consortium (ILCCO), Myriam Brossard, Mark M. Iles, Matthew H. Law, Stuart MacGregor, Melanoma GWAS Consortium, Jonathan Beesley, Michelle R. Jones, Jonathan Tyrer, Stacey J. Winham, Ovarian Cancer Association Consortium (OCAC), Alison P. Klein, Gloria Petersen, Donghui Li, Brian M. Wolpin, Pancreatic Cancer Case-Control Consortium (PANC4), Pancreatic Cancer Cohort Consortium (PanScan), Rosalind A. Eeles, Christopher A. Haiman, Zsofia Kote-Jarai, Fredrick R. Schumacher, PRACTICAL consortium, CRUK, BPC3, CAPS, PEGASUS, Paul Brennan, Stephen J. Chanock, Valerie Gaborieau, Mark P. Purdue, Renal Cancer GWAS Consortium, Paul Pharoah, Rayjean J. Hung, Laufey T. Amundadottir, Peter Kraft, Bogdan Pasaniuc, and Sara Lindström

	Breast, Overall	Breast, ER-neg	Breast, ER-pos	Colorectal	Endometrial	Esophageal	Glioma	Head/Neck	Lung	Melanoma	Ovarian	Pancreatic	Prostate	Renal
Breast, Overall	1													
Breast, ER-negative	**NA**	1												
Breast, ER-positive	**NA**	**NA**	1											
Colorectal	0.009	0.013	0.001	1										
Endometrial	**0.084**	0.044	**0.064**	0.014	1									
Esophageal	0.020	0.007	0.004	0.009	0.016	1								
Glioma	0.017	0.015	0.010	0.012	0.004	0.010	1							
Head/Neck	0.013	0.005	0.012	0.007	0.002	0.002	0.007	1						
Lung	0.031	0.019	0.021	0.025	0.021	0.023	0.024	**0.072**	1					
Melanoma	0.027	0.012	0.010	0.003	0.025	**0.081**	0.039	-0.004	0.032	1				
Ovarian	**0.051**	0.030	0.048	0.007	0.040	0.001	0.008	-0.001	0.013	0.012	1			
Pancreatic	0.001	-0.001	-0.001	0.011	0.009	0.003	0.016	0.015	0.023	-0.002	0.003	1		
Prostate	0.004	0.002	0.004	0.001	0.002	0.003	0.009	0.013	-0.005	0.009	-0.005	0.009	1	
Renal	0.027	0.015	0.010	0.013	0.033	0.023	0.021	0.004	**0.071**	0.047	0.011	0.044	0.033	1

Table S1. Correlation between 14 cancer-specific GWAS summary statistics due to sample overlaps, using the tetrachoric correlation between binary-transformed GWAS summary z-scores. Correlations higher than 0.05 are annotated in bold.

Cancer Type	Signal Index	SNP	Position	Ref/Eff	Unconditioned P-value	Conditioned P-value
ER-negative Breast	1	rs10069690	1279790	C/T	1.34E-35	1.34E-35
	2	rs2736107	1297854	C/T	1.23E-20	2.22E-16
Colorectal	1	rs2735940	1296486	A/G	1.17E-24	1.17E-24
	2	rs34156553	1243245	C/T	1.60E-11	2.90E-09
Glioma	1	rs10069690	1279790	C/T	2.32E-66	2.32E-66
	2	rs2853677	1287194	G/A	1.08E-28	2.87E-14
Lung	1	rs380286	1320247	G/A	1.51E-32	1.51E-32
	2	rs7705526	1285974	C/A	1.01E-18	3.38E-16
Melanoma	1	rs380286	1320247	G/A	1.66E-17	1.66E-17
Ovarian	1	rs4449583	1284135	C/T	5.93E-12	5.93E-12
Pancreatic	1	rs31490	1344458	G/A	1.02E-17	1.02E-17
	2	rs2735940	1296486	A/G	2.67E-15	4.59E-09
Prostate	1	rs2242652	1280028	G/A	3.46E-52	3.46E-52
	2	rs34785213	1284149	AC/A	3.31E-21	5.74E-15
	3	rs71595003	1292118	G/A	1.78E-16	3.75E-13
	4	rs74682426	1289975	A/C	7.59E-46	2.41E-09
	5	rs2853677	1287194	G/A	1.53E-02	1.97E-09

Table S2. Conditional analysis identified independent signals of individual cancer at the 5p15.33 region.

Cancer 1	Cancer 2	# of SNP	PP.H0.abf	PP.H1.abf	PP.H2.abf	PP.H3.abf	PP.H4.abf
ERnegBreast	Colorectal	4,427	0.353	0.220	0.249	0.155	0.023
ERnegBreast	Glioma	4,500	0.046	0.029	0.540	0.341	0.043
ERnegBreast	Lung	4,588	0.160	0.101	0.367	0.233	0.140
ERnegBreast	Melanoma	4,112	0.285	0.134	0.371	0.174	0.036
ERnegBreast	Ovarian	5,916	0.247	0.208	0.266	0.224	0.056
ERnegBreast	Pancreatic	5,806	0.385	0.320	0.149	0.123	0.022
ERnegBreast	Prostate	5,914	0.069	0.056	0.252	0.206	0.417
Colorectal	Glioma	4,171	0.046	0.031	0.532	0.363	0.028
Colorectal	Lung	4,166	0.149	0.095	0.310	0.197	0.249
Colorectal	Melanoma	3,831	0.259	0.163	0.332	0.208	0.038
Colorectal	Ovarian	4,415	0.393	0.276	0.182	0.128	0.022
Colorectal	Pancreatic	4,312	0.431	0.301	0.127	0.088	0.052
Colorectal	Prostate	4,417	0.129	0.091	0.324	0.228	0.228
Glioma	Lung	4,200	0.032	0.272	0.072	0.604	0.020
Glioma	Melanoma	3,879	0.024	0.188	0.030	0.238	**0.519**
Glioma	Ovarian	4,491	0.051	0.598	0.026	0.300	0.025
Glioma	Pancreatic	4,414	0.063	0.691	0.018	0.202	0.025
Glioma	Prostate	4,491	0.020	0.236	0.050	0.587	0.106
Lung	Melanoma	3,947	0.181	0.378	0.124	0.260	0.057
Lung	Ovarian	4,578	0.153	0.351	0.140	0.322	0.033
Lung	Pancreatic	4,502	0.233	0.534	0.064	0.146	0.022
Lung	Prostate	4,578	0.074	0.169	0.159	0.365	0.233
Melanoma	Ovarian	4,113	0.296	0.385	0.131	0.171	0.017
Melanoma	Pancreatic	4,031	0.471	0.332	0.108	0.076	0.014
Melanoma	Prostate	4,106	0.239	0.305	0.173	0.221	0.063
Ovarian	Pancreatic	5,797	0.344	0.368	0.131	0.140	0.017
Ovarian	Prostate	5,907	0.102	0.110	0.368	0.396	0.024
Pancreatic	Prostate	5,793	0.112	0.043	0.410	0.156	0.279
H0 through H4 reflects the assumptions as: H0: neither cancer has a genetic association in the region; H1/H2: only cancer 1/cancer 2 has a genetic association in the region; H3: both cancers are associated, but with different causal variants; H4: both cancers are associated and share a single causal variant.							

Table S3. Colocalization analysis assessing the posterior probability (PP) of the existence of shared causal variant between cancer pairs at the 5p15.33 region, after controlling cancer-specific signals (listed in Table S2). PP.H4.abf, representing the PP of both cancers sharing a single causal variant, is annotated in **bold** if the value is larger than 0.5.

Cancer Type	Origin of sample	Sample Type	Assay	ENCODE Index	Detail
Glioma	Brain	Tissue	DNase-seq	ENCSR595CSH	*Homo sapiens* brain tissue, embryo (56 days) and male embryo (58 days)
Breast	Breast	Tissue (Epithelial)	DNase-seq	ENCSR600KUR	*Homo sapiens* breast epithelium tissue, female adult (51 years)
Colorectal	Sigmoid Colon	Tissue	DNase-seq	ENCSR276ITP	*Homo sapiens* sigmoid colon tissue, female adult (53 years)
Lung	Lung	Tissue	DNase-seq	ENCFF794QIS	*Homo sapiens* lung tissue, female embryo (76 days)
Ovarian	Ovary	Tissue	DNase-seq	ENCFF173CIO	*Homo sapiens* ovary tissue, female adult (53 years)
Pancreatic	Body of Pancreas	Tissue	DNase-seq	ENCFF706BCL	*Homo sapiens* body of pancreas tissue, female adult (51 years)
Prostate	Prostate	Epithelial Cells	DNase-seq	ENCSR000EPU	*Homo sapiens* epithelial cell of prostate
Melanoma	Lower Leg of Skin	Tissue	DNase-seq	ENCFF129WMG	*Homo sapiens* lower leg skin tissue, female adult (51 years)

Table S4. Tissue-specific open narrow peaks of normal tissues or primary cell lines for the relevant organs, used in the cross-cancer fine-mapping analysis, from the ENCODE project.

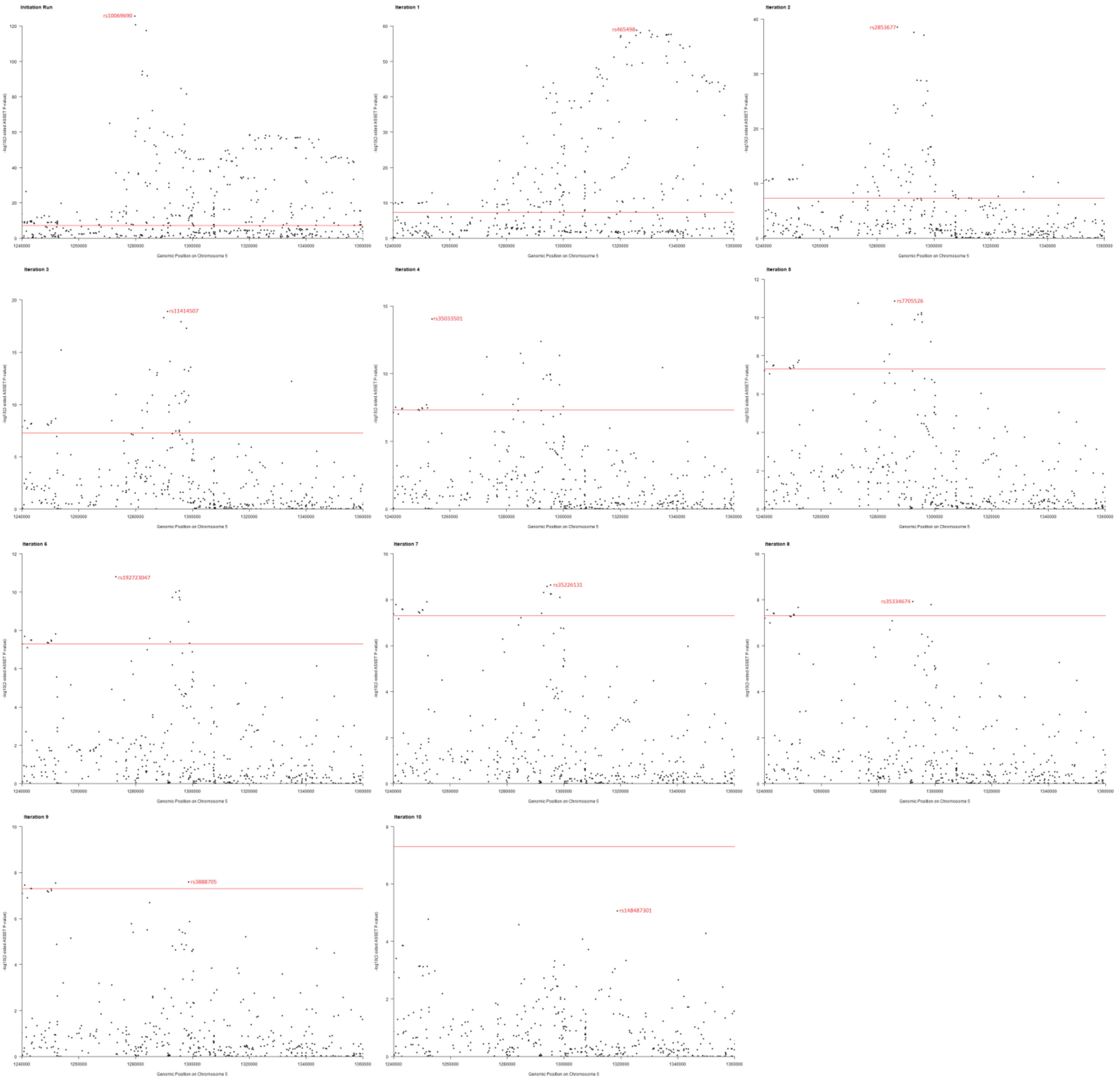

Figure S1. Distribution of two-sided subset-based meta-analysis p-values across eight cancer types at the 5p15.33 region, in each ASSET analysis run. The variant with the smallest p-value in each run (annotated in red) was identified as the index variant of the candidate independent signal, which was further adjusted in the following conditional analysis. The iteration ended when no variant reached genome-wide significant level at p-value = $5x10^{-8}$ (marked with red line in each plot).

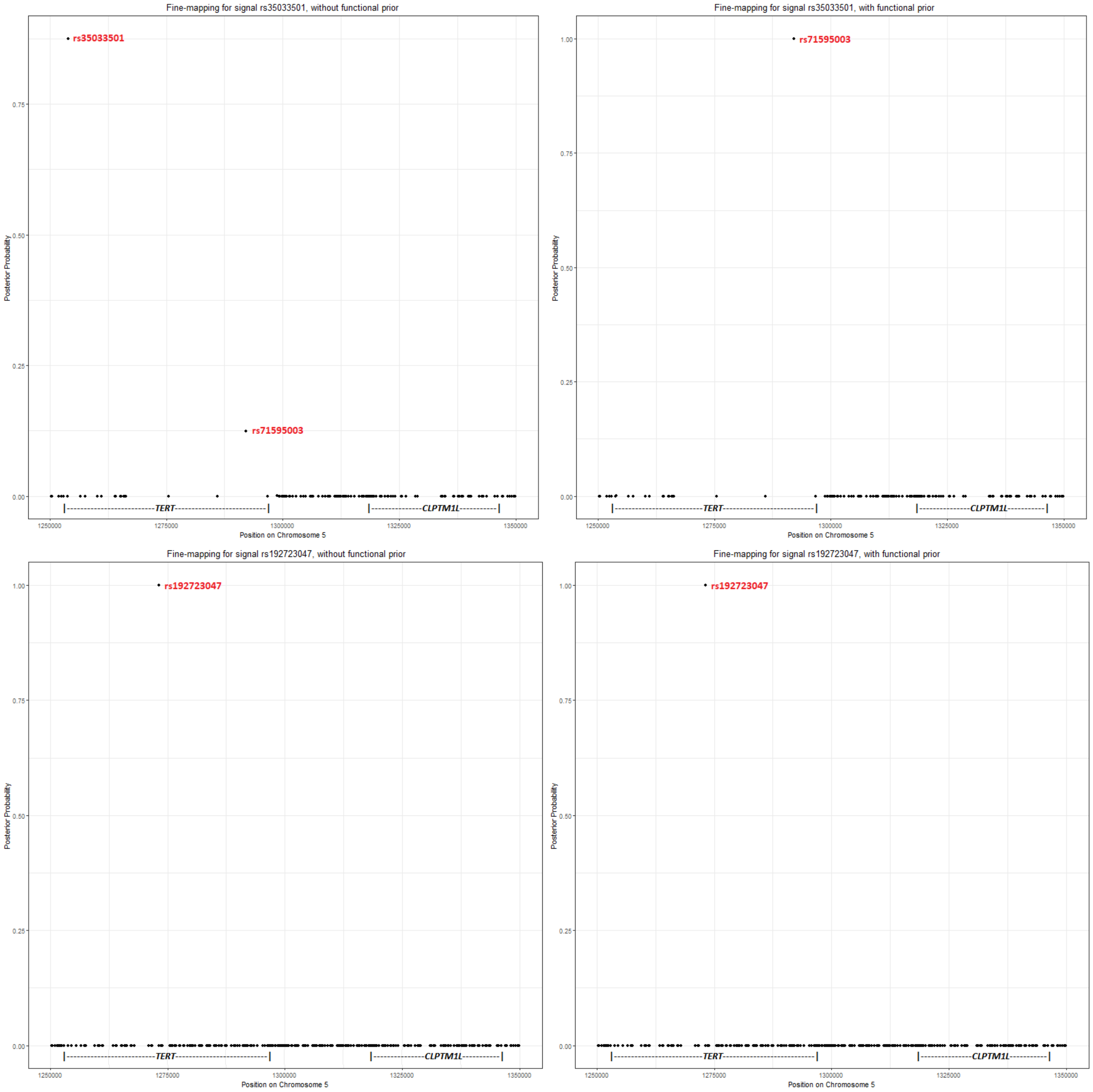
Fine-mapping for signal rs35033501, without functional prior
rs35033501
rs71595003
TERT
CLPTM1L
Posterior Probability
Position on Chromosome 5
Fine-mapping for signal rs35033501, with functional prior
rs71595003
TERT
CLPTM1L
Posterior Probability
Position on Chromosome 5
Fine-mapping for signal rs192723047, without functional prior
rs192723047
TERT
CLPTM1L
Posterior Probability
Position on Chromosome 5
Fine-mapping for signal rs192723047, with functional prior
rs192723047
TERT
CLPTM1L
Posterior Probability
Position on Chromosome 5

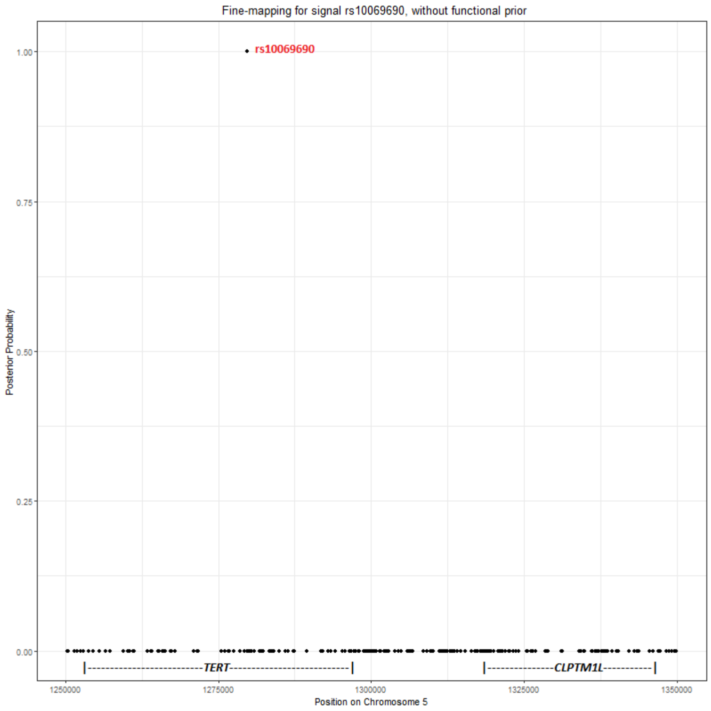
Fine-mapping for signal rs10069690, without functional prior
rs10069690
Posterior Probability
1.00
0.75
0.50
0.25
0.00
TERT
CLPTM1L
1250000
1275000
1300000
1325000
1350000
Position on Chromosome 5

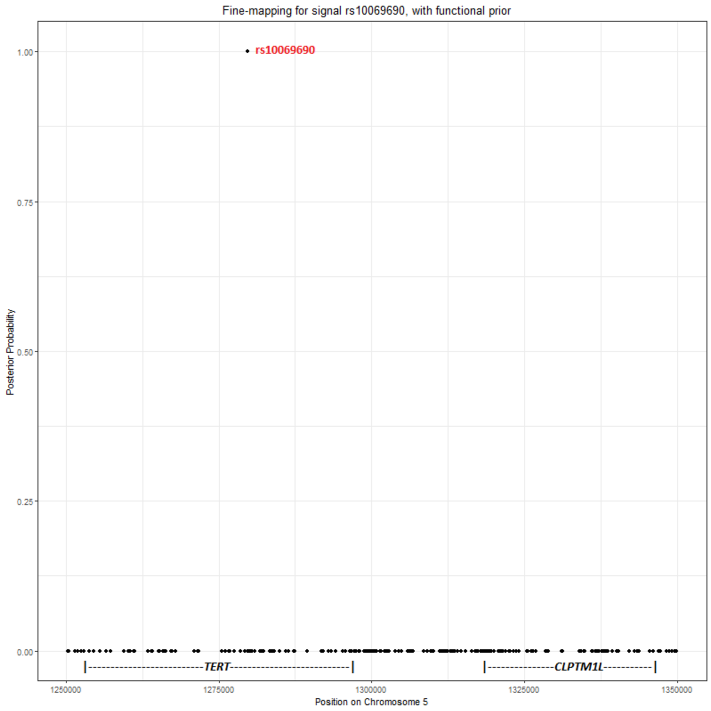
Fine-mapping for signal rs10069690, with functional prior
rs10069690
Posterior Probability
1.00
0.75
0.50
0.25
0.00
TERT
CLPTM1L
1250000
1275000
1300000
1325000
1350000
Position on Chromosome 5

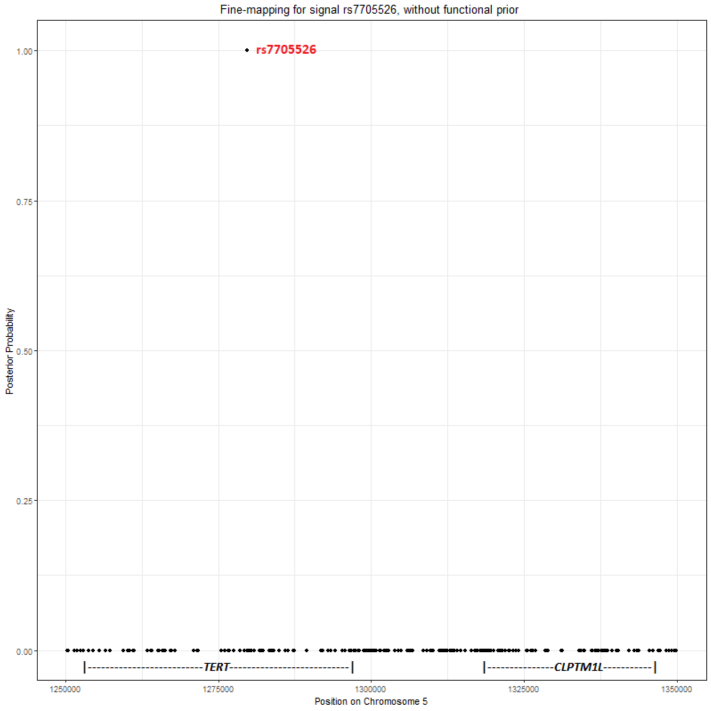
Fine-mapping for signal rs7705526, without functional prior
rs7705526
Posterior Probability
1.00
0.75
0.50
0.25
0.00
TERT
CLPTM1L
1250000
1275000
1300000
1325000
1350000
Position on Chromosome 5

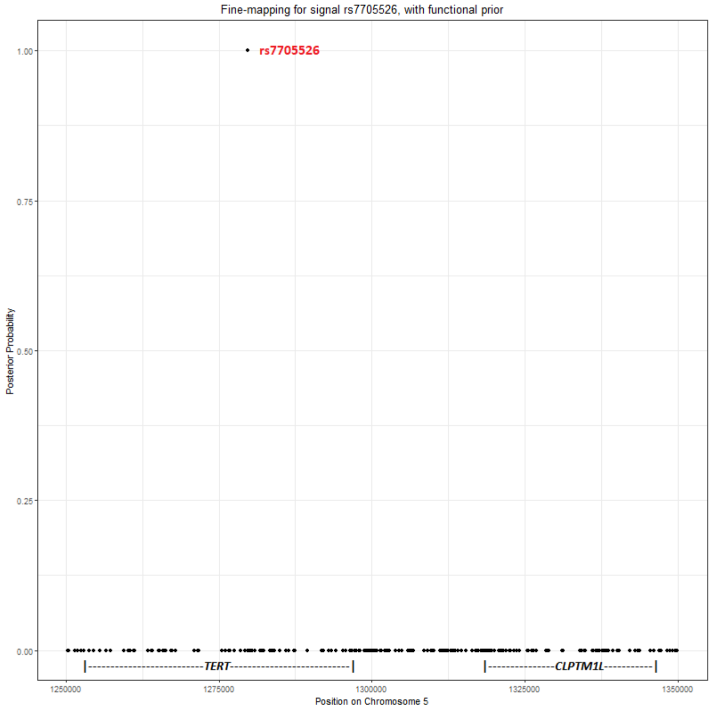
Fine-mapping for signal rs7705526, with functional prior
rs7705526
Posterior Probability
1.00
0.75
0.50
0.25
0.00
TERT
CLPTM1L
1250000
1275000
1300000
1325000
1350000
Position on Chromosome 5

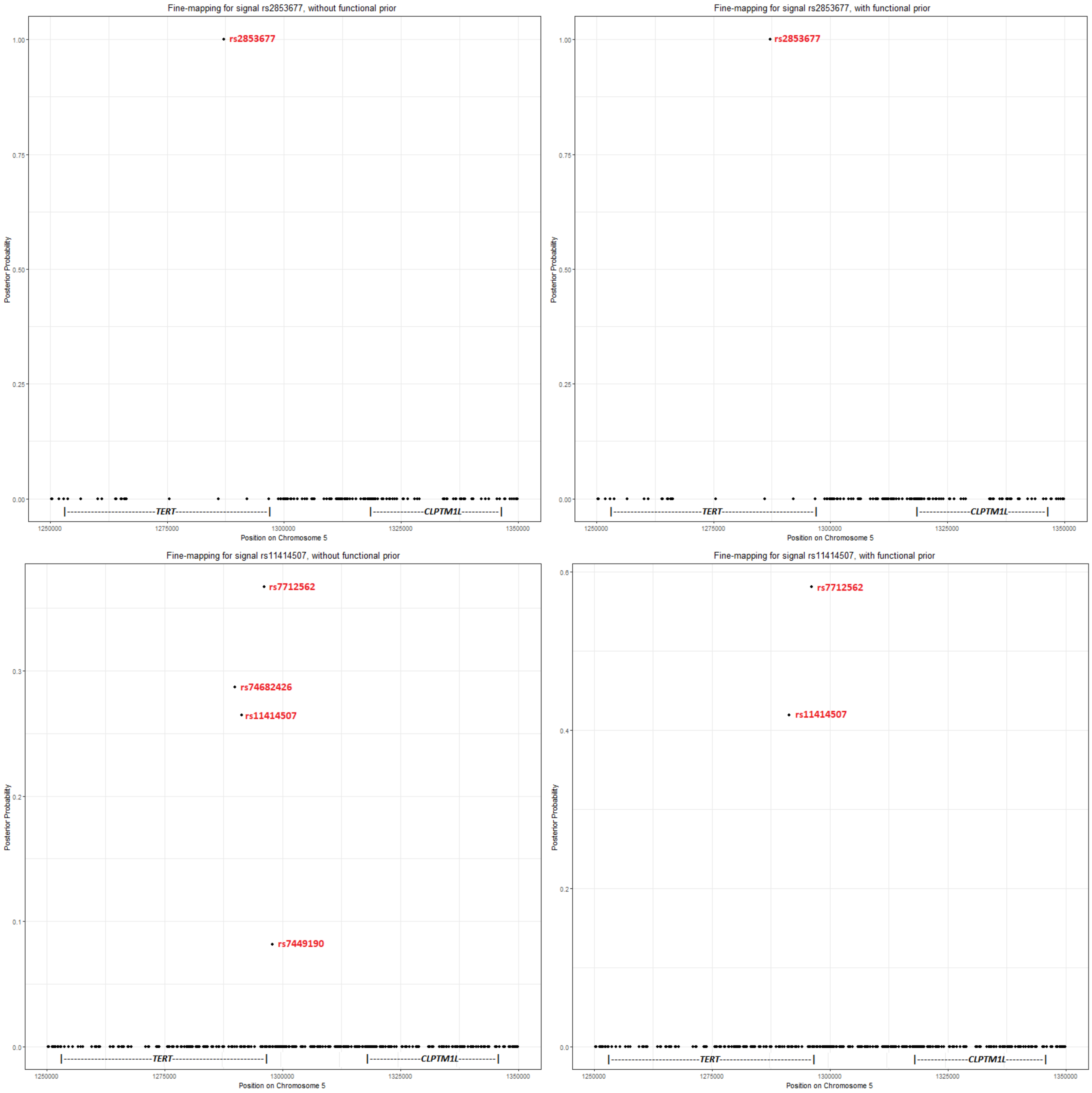

Fine-mapping for signal rs2853677, without functional prior
Fine-mapping for signal rs2853677, with functional prior
Fine-mapping for signal rs11414507, without functional prior
Fine-mapping for signal rs11414507, with functional prior
Posterior Probability
Position on Chromosome 5
1250000
1275000
1300000
1325000
1350000
rs2853677
rs7712562
rs74682426
rs11414507
rs7449190
TERT
CLPTM1L

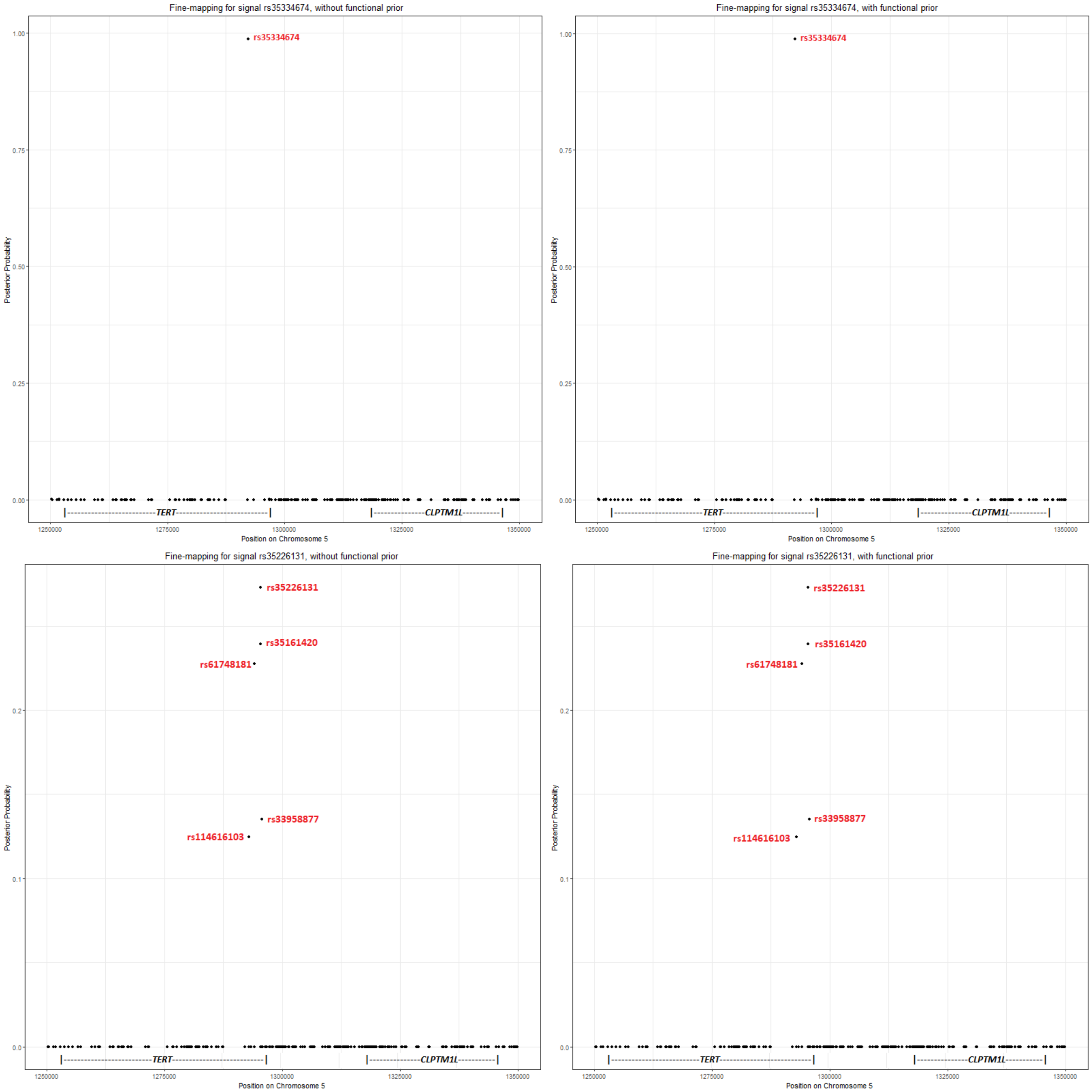
Fine-mapping for signal rs35334674, without functional prior
Posterior Probability
1.00
0.75
0.50
0.25
0.00
rs35334674
TERT
CLPTM1L
1250000
1275000
1300000
1325000
1350000
Position on Chromosome 5
Fine-mapping for signal rs35334674, with functional prior
Posterior Probability
rs35334674
TERT
CLPTM1L
Position on Chromosome 5
Fine-mapping for signal rs35226131, without functional prior
Posterior Probability
0.2
0.1
0.0
rs35226131
rs35161420
rs61748181
rs33958877
rs114616103
TERT
CLPTM1L
Position on Chromosome 5
Fine-mapping for signal rs35226131, with functional prior
Posterior Probability
rs35226131
rs35161420
rs61748181
rs33958877
rs114616103
TERT
CLPTM1L
Position on Chromosome 5

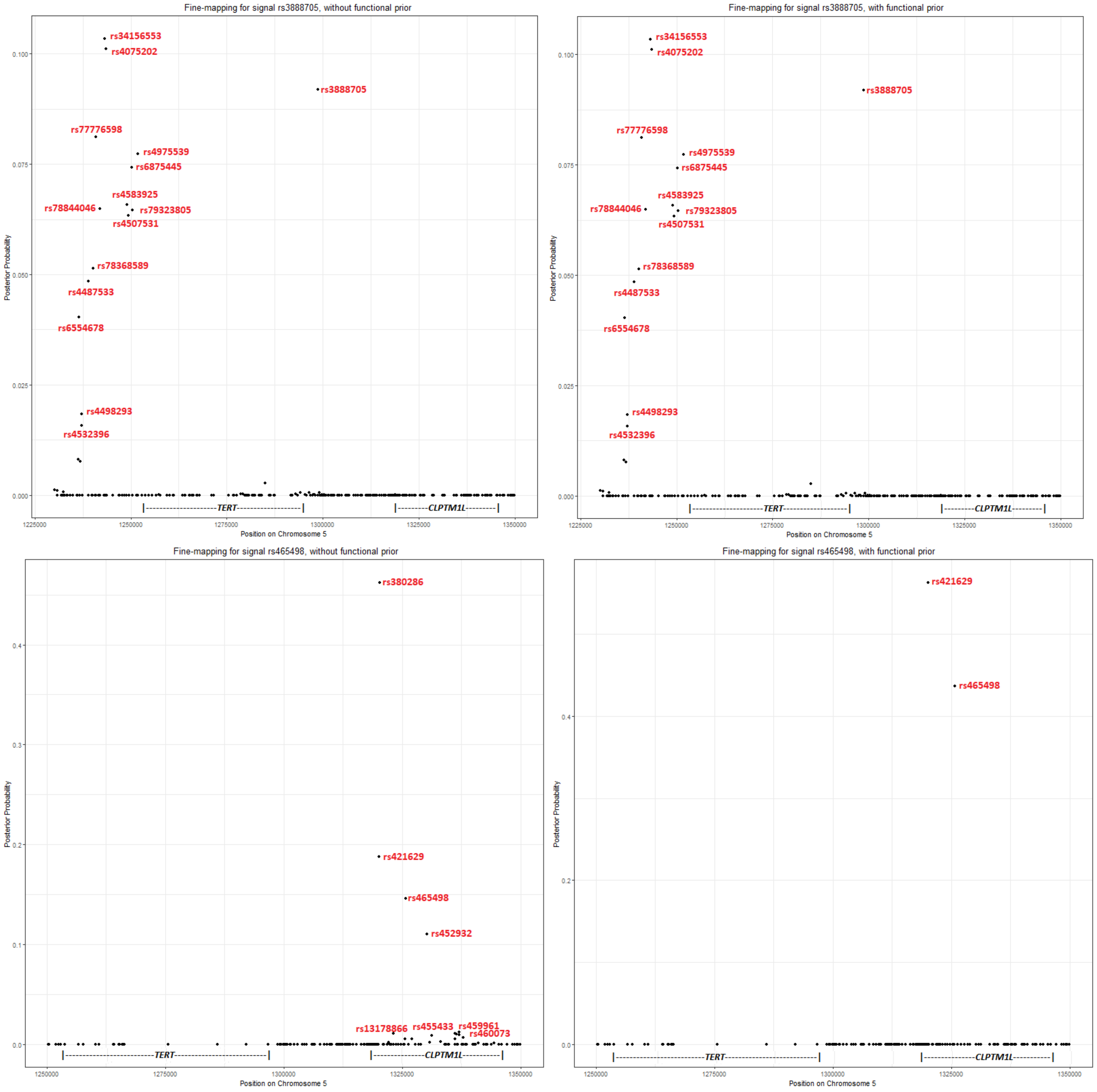

Figure S2. Posterior probability (PP) generated from the fine-mapping analysis for ten independent cross-cancer signals in 5p15.33 region, using PAINTOR V3.0. Each figure shows the fine-mapping results without functional prior (left) or using functional prior (right). Variants included in corresponding 95% PP credible set of each signal were annotated in red

Fundings

This work was supported by CA194393 and CA182821.

BCAC: BCAC is funded by Cancer Research UK [C1287/A16563], the European Union's Horizon 2020 Research and Innovation Programme (grant numbers 634935 and 633784 for BRIDGES and B-CAST respectively), and by the European Community´s Seventh Framework Programme under grant agreement number 223175 (grant number HEALTH-F2-2009-223175) (COGS). The EU Horizon 2020 Research and Innovation Programme funding source had no role in study design, data collection, data analysis, data interpretation or writing of the report. Genotyping of the OncoArray was funded by the NIH Grant U19 CA148065, and Cancer UK Grant C1287/A16563 and the PERSPECTIVE project supported by the Government of Canada through Genome Canada and the Canadian Institutes of Health Research (grant GPH-129344) and, the Ministère de l'Économie, Science et Innovation du Québec through Genome Québec and the PSRSIIRI-701 grant, and the Quebec Breast Cancer Foundation. Funding for the iCOGS infrastructure came from: the European Community's Seventh Framework Programme under grant agreement n° 223175 (HEALTH-F2-2009-223175) (COGS), Cancer Research UK (C1287/A10118, C1287/A10710, C12292/A11174, C1281/A12014, C5047/A8384, C5047/A15007, C5047/A10692, C8197/A16565), the National Institutes of Health (CA128978) and Post-Cancer GWAS initiative (1U19 CA148537, 1U19 CA148065 and 1U19 CA148112 - the GAME-ON initiative), the Department of Defence (W81XWH-10-1-0341), the Canadian Institutes of Health Research (CIHR) for the CIHR Team in Familial Risks of Breast Cancer, and Komen Foundation for the Cure, the Breast Cancer Research Foundation, and the Ovarian Cancer Research Fund. The DRIVE Consortium was funded by U19 CA148065.

BCAC individual studies: The Australian Breast Cancer Family Study (ABCFS) was supported by grant UM1 CA164920 from the National Cancer Institute (USA). The content of this manuscript does not necessarily reflect the views or policies of the National Cancer Institute or any of the collaborating centers in the Breast Cancer Family Registry (BCFR), nor does mention of trade names, commercial products, or organizations imply endorsement by the USA Government or the BCFR. The ABCFS was also supported by the National Health and Medical Research Council of Australia, the New South Wales Cancer Council, the Victorian Health Promotion Foundation (Australia) and the Victorian Breast Cancer Research Consortium. J.L.H. is a National Health and Medical Research Council (NHMRC) Senior Principal Research Fellow. M.C.S. is a NHMRC Senior Research Fellow. The ABCS study was supported by the Dutch Cancer Society [grants NKI 2007-3839; 2009 4363]. The Australian Breast Cancer Tissue Bank (ABCTB) was supported by the National Health and Medical Research Council of Australia, The Cancer Institute NSW and the National Breast Cancer Foundation. The ACP study is funded by the Breast Cancer Research Trust, UK. KM is supported by the NIHR Manchester Biomedical Research Centre, the Allan Turing Institute, and, by the ICEP (Cancer Research UK (C18281/A19169). The work of the BBCC was partly funded by ELAN-Fond of the University Hospital of Erlangen. The BBCS is funded by Cancer Research UK and Breast Cancer Now and acknowledges NHS funding to the NIHR Biomedical Research Centre, and the National Cancer Research Network (NCRN). The BCEES was funded by the National Health and Medical Research Council, Australia and the Cancer Council Western Australia and acknowledges funding from the National Breast Cancer Foundation (JS). For the BCFR-NY, BCFR-PA, BCFR-UT this work was supported by grant UM1 CA164920 from the National Cancer Institute. The content of this manuscript does not necessarily reflect the views or policies of the National Cancer Institute or any of the collaborating centers in the Breast Cancer Family Registry (BCFR), nor does mention of trade names, commercial products, or organizations imply endorsement by the US Government or the BCFR. The BCINIS study is supported in part by the Breast Cancer Research Foundation (BCRF). For BIGGS, ES is supported by NIHR Comprehensive Biomedical Research Centre, Guy's & St. Thomas' NHS

Foundation Trust in partnership with King's College London, United Kingdom. The BREast Oncology GAlician Network (BREOGAN) is funded by Acción Estratégica de Salud del Instituto de Salud Carlos III FIS PI12/02125/Cofinanciado FEDER; Acción Estratégica de Salud del Instituto de Salud Carlos III FIS Intrasalud (PI13/01136); Programa Grupos Emergentes, Cancer Genetics Unit, Instituto de Investigacion Biomedica Galicia Sur. Xerencia de Xestion Integrada de Vigo-SERGAS, Instituto de Salud Carlos III, Spain; Grant 10CSA012E, Consellería de Industria Programa Sectorial de Investigación Aplicada, PEME I + D e I + D Suma del Plan Gallego de Investigación, Desarrollo e Innovación Tecnológica de la Consellería de Industria de la Xunta de Galicia, Spain; Grant EC11-192. Fomento de la Investigación Clínica Independiente, Ministerio de Sanidad, Servicios Sociales e Igualdad, Spain; and Grant FEDER-Innterconecta. Ministerio de Economia y Competitividad, Xunta de Galicia, Spain. The BSUCH study was supported by the Dietmar-Hopp Foundation, the Helmholtz Society and the German Cancer Research Center (DKFZ). CBCS is funded by the Canadian Cancer Society (grant # 313404) and the Canadian Institutes of Health Research. CCGP is supported by funding from the University of Crete. The CECILE study was supported by Fondation de France, Institut National du Cancer (INCa), Ligue Nationale contre le Cancer, Agence Nationale de Sécurité Sanitaire, de l'Alimentation, de l'Environnement et du Travail (ANSES), Agence Nationale de la Recherche (ANR). The CGPS was supported by the Chief Physician Johan Boserup and Lise Boserup Fund, the Danish Medical Research Council, and Herlev and Gentofte Hospital. The CNIO-BCS was supported by the Instituto de Salud Carlos III, the Red Temática de Investigación Cooperativa en Cáncer and grants from the Asociación Española Contra el Cáncer and the Fondo de Investigación Sanitario (PI11/00923 and PI12/00070). The American Cancer Society funds the creation, maintenance, and updating of the CPS-II cohort. The California Teachers Study and the research reported in this publication were supported by the National Cancer Institute of the National Institutes of Health under award number U01-CA199277; P30-CA033572; P30-CA023100; UM1-CA164917; and R01-CA077398. The content is solely the responsibility of the authors and does not necessarily represent the official views of the National Cancer Institute or the National Institutes of Health. The collection of cancer incidence data used in the California Teachers Study was supported by the California Department of Public Health pursuant to California Health and Safety Code Section 103885; Centers for Disease Control and Prevention's National Program of Cancer Registries, under cooperative agreement 5NU58DP006344; the National Cancer Institute's Surveillance, Epidemiology and End Results Program under contract HHSN261201800032I awarded to the University of California, San Francisco, contract HHSN261201800015I awarded to the University of Southern California, and contract HHSN261201800009I awarded to the Public Health Institute. The opinions, findings, and conclusions expressed herein are those of the author(s) and do not necessarily reflect the official views of the State of California, Department of Public Health, the National Cancer Institute, the National Institutes of Health, the Centers for Disease Control and Prevention or their Contractors and Subcontractors, or the Regents of the University of California, or any of its programs. The University of Westminster curates the DietCompLyf database funded by Against Breast Cancer Registered Charity No. 1121258 and the NCRN. The coordination of EPIC is financially supported by the European Commission (DG-SANCO) and the International Agency for Research on Cancer. The national cohorts are supported by: Ligue Contre le Cancer, Institut Gustave Roussy, Mutuelle Générale de l'Education Nationale, Institut National de la Santé et de la Recherche Médicale (INSERM) (France); German Cancer Aid, German Cancer Research Center (DKFZ), Federal Ministry of Education and Research (BMBF) (Germany); the Hellenic Health Foundation, the Stavros Niarchos Foundation (Greece); Associazione Italiana per la Ricerca sul Cancro-AIRC-Italy and National Research Council (Italy); Dutch Ministry of Public Health, Welfare and Sports (VWS), Netherlands Cancer Registry (NKR), LK Research Funds, Dutch Prevention Funds, Dutch ZON (Zorg Onderzoek Nederland), World Cancer Research Fund (WCRF), Statistics Netherlands (The Netherlands); Health Research Fund (FIS), PI13/00061 to Granada, PI13/01162 to EPIC-Murcia, Regional Governments

of Andalucía, Asturias, Basque Country, Murcia and Navarra, ISCIII RETIC (RD06/0020) (Spain); Cancer Research UK (14136 to EPIC-Norfolk; C570/A16491 and C8221/A19170 to EPIC-Oxford), Medical Research Council (1000143 to EPIC-Norfolk, MR/M012190/1 to EPIC-Oxford) (United Kingdom). The ESTHER study was supported by a grant from the Baden Württemberg Ministry of Science, Research and Arts. Additional cases were recruited in the context of the VERDI study, which was supported by a grant from the German Cancer Aid (Deutsche Krebshilfe). The GC-HBOC (German Consortium of Hereditary Breast and Ovarian Cancer) is supported by the German Cancer Aid (grant no 110837, coordinator: Rita K. Schmutzler, Cologne). This work was also funded by the European Regional Development Fund and Free State of Saxony, Germany (LIFE - Leipzig Research Centre for Civilization Diseases, project numbers 713-241202, 713-241202, 14505/2470, 14575/2470). The GENICA was funded by the Federal Ministry of Education and Research (BMBF) Germany grants 01KW9975/5, 01KW9976/8, 01KW9977/0 and 01KW0114, the Robert Bosch Foundation, Stuttgart, Deutsches Krebsforschungszentrum (DKFZ), Heidelberg, the Institute for Prevention and Occupational Medicine of the German Social Accident Insurance, Institute of the Ruhr University Bochum (IPA), Bochum, as well as the Department of Internal Medicine, Evangelische Kliniken Bonn gGmbH, Johanniter Krankenhaus, Bonn, Germany; German Cancer Consortium (DKTK) and German Cancer Research Center (DKFZ), gefördert durch die Deutsche Forschungsgemeinschaft (DFG) im Rahmen der Exzellenzstrategie des Bundes und der Länder - EXC 2180 – 390900677. The GEPARSIXTO study was conducted by the German Breast Group GmbH. The GESBC was supported by the Deutsche Krebshilfe e. V. [70492] and the German Cancer Research Center (DKFZ). The HABCS study was supported by the Claudia von Schilling Foundation for Breast Cancer Research, by the Lower Saxonian Cancer Society, and by the Rudolf Bartling Foundation. The HEBCS was financially supported by the Helsinki University Hospital Research Fund, the Finnish Cancer Society, and the Sigrid Juselius Foundation. The HERPACC was supported by MEXT Kakenhi (No. 170150181 and 26253041) from the Ministry of Education, Science, Sports, Culture and Technology of Japan, by a Grant-in-Aid for the Third Term Comprehensive 10-Year Strategy for Cancer Control from Ministry Health, Labour and Welfare of Japan, by Health and Labour Sciences Research Grants for Research on Applying Health Technology from Ministry Health, Labour and Welfare of Japan, by National Cancer Center Research and Development Fund, and "Practical Research for Innovative Cancer Control (15ck0106177h0001)" from Japan Agency for Medical Research and development, AMED, and Cancer Bio Bank Aichi. The HMBCS was supported by a grant from the Friends of Hannover Medical School and by the Rudolf Bartling Foundation. The HUBCS was supported by a grant from the German Federal Ministry of Research and Education (RUS08/017), and the study was performed as part of the assignment of the Ministry of Science and Higher Education of the Russian Federation (№AAAA-A16-116020350032-1). Financial support for KARBAC was provided through the regional agreement on medical training and clinical research (ALF) between Stockholm County Council and Karolinska Institutet, the Swedish Cancer Society, The Gustav V Jubilee foundation and Bert von Kantzows foundation. The KARMA study was supported by Märit and Hans Rausings Initiative Against Breast Cancer. The KBCP was financially supported by the special Government Funding (EVO) of Kuopio University Hospital grants, Cancer Fund of North Savo, the Finnish Cancer Organizations, and by the strategic funding of the University of Eastern Finland. kConFab is supported by a grant from the National Breast Cancer Foundation, and previously by the National Health and Medical Research Council (NHMRC), the Queensland Cancer Fund, the Cancer Councils of New South Wales, Victoria, Tasmania and South Australia, and the Cancer Foundation of Western Australia. Financial support for the AOCS was provided by the United States Army Medical Research and Materiel Command [DAMD17-01-1-0729], Cancer Council Victoria, Queensland Cancer Fund, Cancer Council New South Wales, Cancer Council South Australia, The Cancer Foundation of Western Australia, Cancer Council Tasmania and the National Health and Medical Research Council of Australia (NHMRC; 400413, 400281, 199600). G.C-T. is supported by the NHMRC. The KOHBRA study was partially supported by a grant

from the Korea Health Technology R&D Project through the Korea Health Industry Development Institute (KHIDI), and the National R&D Program for Cancer Control, Ministry of Health & Welfare, Republic of Korea (HI16C1127; 1020350; 1420190). LAABC is supported by grants (1RB-0287, 3PB-0102, 5PB-0018, 10PB-0098) from the California Breast Cancer Research Program. Incident breast cancer cases were collected by the USC Cancer Surveillance Program (CSP) which is supported under subcontract by the California Department of Health. The CSP is also part of the National Cancer Institute's Division of Cancer Prevention and Control Surveillance, Epidemiology, and End Results Program, under contract number N01CN25403. LMBC is supported by the 'Stichting tegen Kanker'. DL is supported by the FWO. The MABCS study is funded by the Research Centre for Genetic Engineering and Biotechnology "Georgi D. Efremov", MASA. The MARIE study was supported by the Deutsche Krebshilfe e.V. [70-2892-BR I, 106332, 108253, 108419, 110826, 110828], the Hamburg Cancer Society, the German Cancer Research Center (DKFZ) and the Federal Ministry of Education and Research (BMBF) Germany [01KH0402]. MBCSG is supported by grants from the Italian Association for Cancer Research (AIRC). The MCBCS was supported by the NIH grants CA192393, CA116167, CA176785 an NIH Specialized Program of Research Excellence (SPORE) in Breast Cancer [CA116201], and the Breast Cancer Research Foundation and a generous gift from the David F. and Margaret T. Grohne Family Foundation. The Melbourne Collaborative Cohort Study (MCCS) cohort recruitment was funded by VicHealth and Cancer Council Victoria. The MCCS was further augmented by Australian National Health and Medical Research Council grants 209057, 396414 and 1074383 and by infrastructure provided by Cancer Council Victoria. Cases and their vital status were ascertained through the Victorian Cancer Registry and the Australian Institute of Health and Welfare, including the National Death Index and the Australian Cancer Database. The MEC was supported by NIH grants CA63464, CA54281, CA098758, CA132839 and CA164973. The MISS study is supported by funding from ERC-2011-294576 Advanced grant, Swedish Cancer Society, Swedish Research Council, Local hospital funds, Berta Kamprad Foundation, Gunnar Nilsson. The MMHS study was supported by NIH grants CA97396, CA128931, CA116201, CA140286 and CA177150. The work of MTLGEBCS was supported by the Quebec Breast Cancer Foundation, the Canadian Institutes of Health Research for the "CIHR Team in Familial Risks of Breast Cancer" program – grant # CRN-87521 and the Ministry of Economic Development, Innovation and Export Trade – grant # PSR-SIIRI-701. MYBRCA is funded by research grants from the Malaysian Ministry of Higher Education (UM.C/HlR/MOHE/06) and Cancer Research Malaysia. MYMAMMO is supported by research grants from Yayasan Sime Darby LPGA Tournament and Malaysian Ministry of Higher Education (RP046B-15HTM). The NBCS has received funding from the K.G. Jebsen Centre for Breast Cancer Research; the Research Council of Norway grant 193387/V50 (to A-L Børresen-Dale and V.N. Kristensen) and grant 193387/H10 (to A-L Børresen-Dale and V.N. Kristensen), South Eastern Norway Health Authority (grant 39346 to A-L Børresen-Dale) and the Norwegian Cancer Society (to A-L Børresen-Dale and V.N. Kristensen). The NBHS was supported by NIH grant R01CA100374. Biological sample preparation was conducted the Survey and Biospecimen Shared Resource, which is supported by P30 CA68485. The Northern California Breast Cancer Family Registry (NC-BCFR) and Ontario Familial Breast Cancer Registry (OFBCR) were supported by grant U01CA164920 from the USA National Cancer Institute of the National Institutes of Health. The content of this manuscript does not necessarily reflect the views or policies of the National Cancer Institute or any of the collaborating centers in the Breast Cancer Family Registry (BCFR), nor does mention of trade names, commercial products, or organizations imply endorsement by the USA Government or the BCFR. The NGOBCS was supported by the National Cancer Center Research and Development Fund (Japan). The NHS was supported by NIH grants P01 CA87969, UM1 CA186107, and U19 CA148065. The NHS2 was supported by NIH grants UM1 CA176726 and U19 CA148065. The OBCS was supported by research grants from the Finnish Cancer Foundation, the Academy of Finland (grant number 250083, 122715 and Center of Excellence grant number 251314), the Finnish Cancer Foundation, the Sigrid Juselius Foundation,

the University of Oulu, the University of Oulu Support Foundation and the special Governmental EVO funds for Oulu University Hospital-based research activities. The ORIGO study was supported by the Dutch Cancer Society (RUL 1997-1505) and the Biobanking and Biomolecular Resources Research Infrastructure (BBMRI-NL CP16). The PBCS was funded by Intramural Research Funds of the National Cancer Institute, Department of Health and Human Services, USA. Genotyping for PLCO was supported by the Intramural Research Program of the National Institutes of Health, NCI, Division of Cancer Epidemiology and Genetics. The PLCO is supported by the Intramural Research Program of the Division of Cancer Epidemiology and Genetics and supported by contracts from the Division of Cancer Prevention, National Cancer Institute, National Institutes of Health. The POSH study is funded by Cancer Research UK (grants C1275/A11699, C1275/C22524, C1275/A19187, C1275/A15956 and Breast Cancer Campaign 2010PR62, 2013PR044. The RBCS was funded by the Dutch Cancer Society (DDHK 2004-3124, DDHK 2009-4318). The SASBAC study was supported by funding from the Agency for Science, Technology and Research of Singapore (A*STAR), the US National Institute of Health (NIH) and the Susan G. Komen Breast Cancer Foundation. The SBCGS was supported primarily by NIH grants R01CA64277, R01CA148667, UMCA182910, and R37CA70867. Biological sample preparation was conducted the Survey and Biospecimen Shared Resource, which is supported by P30 CA68485. The scientific development and funding of this project were, in part, supported by the Genetic Associations and Mechanisms in Oncology (GAME-ON) Network U19 CA148065. The SBCS was supported by Sheffield Experimental Cancer Medicine Centre and Breast Cancer Now Tissue Bank. SEARCH is funded by Cancer Research UK [C490/A10124, C490/A16561] and supported by the UK National Institute for Health Research Biomedical Research Centre at the University of Cambridge. The University of Cambridge has received salary support for P.D.P.P. from the NHS in the East of England through the Clinical Academic Reserve. SEBCS was supported by the BRL (Basic Research Laboratory) program through the National Research Foundation of Korea funded by the Ministry of Education, Science and Technology (2012-0000347). SGBCC is funded by the National Research Foundation Singapore, NUS start-up Grant, National University Cancer Institute Singapore (NCIS) Centre Grant, Breast Cancer Prevention Programme, Asian Breast Cancer Research Fund and the NMRC Clinician Scientist Award (SI Category). Additional controls were recruited by the Singapore Consortium of Cohort Studies-Multi-ethnic cohort (SCCS-MEC), which was funded by the Biomedical Research Council, grant number: 05/1/21/19/425. The Sister Study (SISTER) is supported by the Intramural Research Program of the NIH, National Institute of Environmental Health Sciences (Z01-ES044005 and Z01-ES049033). The Two Sister Study (2SISTER) was supported by the Intramural Research Program of the NIH, National Institute of Environmental Health Sciences (Z01-ES044005 and Z01-ES102245), and, also by a grant from Susan G. Komen for the Cure, grant FAS0703856. SKKDKFZS is supported by the DKFZ. The SMC is funded by the Swedish Cancer Foundation and the Swedish Research Council (VR 2017-00644) grant for the Swedish Infrastructure for Medical Population-based Life-course Environmental Research (SIMPLER). The SZBCS was supported by Grant PBZ_KBN_122/P05/2004 and the program of the Minister of Science and Higher Education under the name "Regional Initiative of Excellence" in 2019-2022 project number 002/RID/2018/19 amount of financing 12 000 000 PLN. The TNBCC was supported by: a Specialized Program of Research Excellence (SPORE) in Breast Cancer (CA116201), a grant from the Breast Cancer Research Foundation, a generous gift from the David F. and Margaret T. Grohne Family Foundation. The TWBCS is supported by the Taiwan Biobank project of the Institute of Biomedical Sciences, Academia Sinica, Taiwan. The UCIBCS component of this research was supported by the NIH [CA58860, CA92044] and the Lon V Smith Foundation [LVS39420]. The UKBGS is funded by Breast Cancer Now and the Institute of Cancer Research (ICR), London. ICR acknowledges NHS funding to the NIHR Biomedical Research Centre. The WHI program is funded by the National Heart, Lung, and Blood Institute, the US National Institutes of Health and the US Department of Health and Human Services (HHSN268201100046C,

HHSN268201100001C, HHSN268201100002C, HHSN268201100003C, HHSN268201100004C and HHSN271201100004C). This work was also funded by NCI U19 CA148065-01.

BPC3: The BPC3 was supported by the U.S. National Institutes of Health, National Cancer Institute (cooperative agreements U01-CA98233 to D.J.H., U01-CA98710 to S.M.G., U01-CA98216 to E.R., and U01-CA98758 to B.E.H., and Intramural Research Program of NIH/National Cancer Institute, Division of Cancer Epidemiology and Genetics).

CAPS: CAPS GWAS study was supported by the Cancer Risk Prediction Center (CRisP; www.crispcenter.org), a Linneus Centre (Contract ID 70867902) financed by the Swedish Research Council, (grant no K2010-70X-20430-04-3), the Swedish Cancer Foundation (grant no 09-0677), the Hedlund Foundation, the Soederberg Foundation, the Enqvist Foundation, ALF funds from the Stockholm County Council. Stiftelsen Johanna Hagstrand och Sigfrid Linner's Minne, Karlsson's Fund for urological and surgical research.

CRUK and PRACTICAL consortium: This work was supported by the Canadian Institutes of Health Research, European Commission's Seventh Framework Programme grant agreement n° 223175 (HEALTH-F2-2009-223175), Cancer Research UK Grants C5047/A7357, C1287/A10118, C1287/A16563, C5047/A3354, C5047/A10692, C16913/A6135, and The National Institute of Health (NIH) Cancer Post-Cancer GWAS initiative grant: No. 1 U19 CA 148537-01 (the GAME-ON initiative). We would also like to thank the following for funding support: The Institute of Cancer Research and The Everyman Campaign, The Prostate Cancer Research Foundation, Prostate Research Campaign UK (now PCUK), The Orchid Cancer Appeal, Rosetrees Trust, The National Cancer Research Network UK, The National Cancer Research Institute (NCRI) UK. We are grateful for support of NIHR funding to the NIHR Biomedical Research Centre at The Institute of Cancer Research and The Royal Marsden NHS Foundation Trust. The Prostate Cancer Program of Cancer Council Victoria also acknowledge grant support from The National Health and Medical Research Council, Australia (126402, 209057, 251533, , 396414, 450104, 504700, 504702, 504715, 623204, 940394, 614296,), VicHealth, Cancer Council Victoria, The Prostate Cancer Foundation of Australia, The Whitten Foundation, PricewaterhouseCoopers, and Tattersall's. EAO, DMK, and EMK acknowledge the Intramural Program of the National Human Genome Research Institute for their support. Genotyping of the OncoArray was funded by the US National Institutes of Health (NIH) [U19 CA 148537 for ELucidating Loci Involved in Prostate cancer SuscEptibility (ELLIPSE) project and X01HG007492 to the Center for Inherited Disease Research (CIDR) under contract number HHSN268201200008I]. Additional analytic support was provided by NIH NCI U01 CA188392 (PI: Schumacher). Funding for the iCOGS infrastructure came from: the European Community's Seventh Framework Programme under grant agreement n° 223175 (HEALTH-F2-2009-223175) (COGS), Cancer Research UK (C1287/A10118, C1287/A 10710, C12292/A11174, C1281/A12014, C5047/A8384, C5047/A15007, C5047/A10692, C8197/A16565), the National Institutes of Health (CA128978) and Post-Cancer GWAS initiative (1U19 CA148537, 1U19 CA148065 and 1U19 CA148112 - the GAME-ON initiative), the Department of Defence (W81XWH-10-1-0341), the Canadian Institutes of Health Research (CIHR) for the CIHR Team in Familial Risks of Breast Cancer, Komen Foundation for the Cure, the Breast Cancer Research Foundation, and the Ovarian Cancer Research Fund.

ECAC: The Endometrial Cancer Association Consortium genome-wide association analyses were supported by the National Health and Medical Research Council of Australia (APP552402, APP1031333, APP110`9286, APP1111246 and APP1061779), the U.S. National Institutes of Health (R01-CA134958), European Research Council (EU FP7 Grant), Wellcome Trust Centre for Human Genetics (090532/Z/09Z) and Cancer Research UK. OncoArray genotyping of ECAC cases was performed with the generous assistance of the Ovarian Cancer Association Consortium (OCAC), which was funded through grants from

the U.S. National Institutes of Health (CA1X01HG007491-01 (C.I. Amos), U19-CA148112 (T.A. Sellers), R01-CA149429 (C.M. Phelan) and R01-CA058598 (M.T. Goodman); Canadian Institutes of Health Research (MOP-86727 (L.E. Kelemen)) and the Ovarian Cancer Research Fund (A. Berchuck). We particularly thank the efforts of Cathy Phelan. OncoArray genotyping of the BCAC controls was funded by Genome Canada Grant GPH-129344, NIH Grant U19 CA148065, and Cancer UK Grant C1287/A16563. All studies and funders are listed in O'Mara et al (2018).

CCFR, CORECT and GECCO: The Genetics and Epidemiology of Colorectal Cancer Consortium (GECCO) was supported by grants from the National Cancer Institute, National Institutes of Health, U.S. Department of Health and Human Services (U01 CA137088, R01 CA059045, R01 CA201407, R01 CA244588). This research was funded in part through the NIH/NCI Cancer Center Support Grant P30 CA015704. Scientific Computing Infrastructure at Fred Hutch funded by ORIP grant S10OD028685. The ATBC Study is supported by the Intramural Research Program of the U.S. National Cancer Institute, National Institutes of Health, Department of Health and Human Services. CLUE funding was from the National Cancer Institute (U01 CA86308, Early Detection Research Network; P30 CA006973), National Institute on Aging (U01 AG18033), and the American Institute for Cancer Research. The content of this publication does not necessarily reflect the views or policies of the Department of Health and Human Services, nor does mention of trade names, commercial products, or organizations imply endorsement by the US government. ColoCare: This work was supported by the National Institutes of Health (grant numbers R01 CA189184 (Li/Ulrich), U01 CA206110 (Ulrich/Li/Siegel/Figueiredo/Colditz, 2P30CA015704- 40 (Gilliland), R01 CA207371 (Ulrich/Li)), the Matthias Lackas-Foundation, the German Consortium for Translational Cancer Research, and the EU TRANSCAN initiative. The Colon Cancer Family Registry (CCFR, www.coloncfr.org) is supported in part by funding from the National Cancer Institute (NCI), National Institutes of Health (NIH) (award U01 CA167551). The CCFR Set-1 (Illumina 1M/1M-Duo) and Set-2 (Illumina Omni1-Quad) scans were supported by NIH awards U01 CA122839 and R01 CA143247 (to GC). The CCFR Set-3 (Affymetrix Axiom CORECT Set array) was supported by NIH award U19 CA148107 and R01 CA81488 (to SBG). The CCFR Set-4 (Illumina OncoArray 600K SNP array) was supported by NIH award U19 CA148107 (to SBG) and by the Center for Inherited Disease Research (CIDR), which is funded by the NIH to the Johns Hopkins University, contract number HHSN268201200008I. The content of this manuscript does not necessarily reflect the views or policies of the NCI, NIH or any of the collaborating centers in the Colon Cancer Family Registry (CCFR), nor does mention of trade names, commercial products, or organizations imply endorsement by the US Government, any cancer registry, or the CCFR. COLON: The COLON study is sponsored by Wereld Kanker Onderzoek Fonds, including funds from grant 2014/1179 as part of the World Cancer Research Fund International Regular Grant Programme, by Alpe d'Huzes and the Dutch Cancer Society (UM 2012–5653, UW 2013-5927, UW2015-7946), and by TRANSCAN (JTC2012-MetaboCCC, JTC2013-FOCUS). The Nqplus study is sponsored by a ZonMW investment grant (98-10030); by PREVIEW, the project PREVention of diabetes through lifestyle intervention and population studies in Europe and around the World (PREVIEW) project which received funding from the European Union Seventh Framework Programme (FP7/2007–2013) under grant no. 312057; by funds from TI Food and Nutrition (cardiovascular health theme), a public–private partnership on precompetitive research in food and nutrition; and by FOODBALL, the Food Biomarker Alliance, a project from JPI Healthy Diet for a Healthy Life. Colorectal Cancer Transdisciplinary (CORECT) Study: The CORECT Study was supported by the National Cancer Institute, National Institutes of Health (NCI/NIH), U.S. Department of Health and Human Services (grant numbers U19 CA148107, R01 CA81488, P30 CA014089, R01 CA197350; P01 CA196569; R01 CA201407) and National Institutes of Environmental Health Sciences, National Institutes of Health (grant number T32 ES013678). CORSA: The CORSA study was funded by Austrian Research Funding Agency (FFG) BRIDGE (grant 829675, to

Andrea Gsur), the "Herzfelder'sche Familienstiftung" (grant to Andrea Gsur) and was supported by COST Action BM1206. CPS-II: The American Cancer Society funds the creation, maintenance, and updating of the Cancer Prevention Study-II (CPS-II) cohort. This study was conducted with Institutional Review Board approval. CRCGEN: Colorectal Cancer Genetics & Genomics, Spanish study was supported by Instituto de Salud Carlos III, co-funded by FEDER funds –a way to build Europe– (grants PI14-613 and PI09-1286), Agency for Management of University and Research Grants (AGAUR) of the Catalan Government (grant 2017SGR723), and Junta de Castilla y León (grant LE22A10-2). Sample collection of this work was supported by the Xarxa de Bancs de Tumors de Catalunya sponsored by Pla Director d'Oncología de Catalunya (XBTC), Plataforma Biobancos PT13/0010/0013 and ICOBIOBANC, sponsored by the Catalan Institute of Oncology. Czech Republic CCS: This work was supported by the Grant Agency of the Czech Republic (18-09709S, 20-03997S), by the Grant Agency of the Ministry of Health of the Czech Republic (grants AZV NV18/03/00199 and AZV NV19-09-00237), and Charles University grants Unce/Med/006 and Progress Q28/LF1. DACHS: This work was supported by the German Research Council (BR 1704/6-1, BR 1704/6-3, BR 1704/6-4, CH 117/1-1, HO 5117/2-1, HE 5998/2-1, KL 2354/3-1, RO 2270/8-1 and BR 1704/17-1), the Interdisciplinary Research Program of the National Center for Tumor Diseases (NCT), Germany, and the German Federal Ministry of Education and Research (01KH0404, 01ER0814, 01ER0815, 01ER1505A and 01ER1505B). DALS: National Institutes of Health (R01 CA48998 to M. L. Slattery). EDRN: This work is funded and supported by the NCI, EDRN Grant (U01 CA 84968-06). EPIC: The coordination of EPIC is financially supported by International Agency for Research on Cancer (IARC) and also by the Department of Epidemiology and Biostatistics, School of Public Health, Imperial College London which has additional infrastructure support provided by the NIHR Imperial Biomedical Research Centre (BRC). The national cohorts are supported by: Danish Cancer Society (Denmark); Ligue Contre le Cancer, Institut Gustave Roussy, Mutuelle Générale de l'Education Nationale, Institut National de la Santé et de la Recherche Médicale (INSERM) (France); German Cancer Aid, German Cancer Research Center (DKFZ), German Institute of Human Nutrition Potsdam- Rehbruecke (DIfE), Federal Ministry of Education and Research (BMBF) (Germany); Associazione Italiana per la Ricerca sul Cancro-AIRC-Italy, Compagnia di SanPaolo and National Research Council (Italy); Dutch Ministry of Public Health, Welfare and Sports (VWS), Netherlands Cancer Registry (NKR), LK Research Funds, Dutch Prevention Funds, Dutch ZON (Zorg Onderzoek Nederland), World Cancer Research Fund (WCRF), Statistics Netherlands (The Netherlands); Health Research Fund (FIS) - Instituto de Salud Carlos III (ISCIII), Regional Governments of Andalucía, Asturias, Basque Country, Murcia and Navarra, and the Catalan Institute of Oncology - ICO (Spain); Swedish Cancer Society, Swedish Research Council and County Councils of Skåne and Västerbotten (Sweden); Cancer Research UK (14136 to EPIC-Norfolk; C8221/A29017 to EPIC-Oxford), Medical Research Council (1000143 to EPIC-Norfolk; MR/M012190/1 to EPIC-Oxford). (United Kingdom). EPICOLON: This work was supported by grants from Fondo de Investigación Sanitaria/FEDER (PI08/0024, PI08/1276, PS09/02368, P111/00219, PI11/00681, PI14/00173, PI14/00230, PI17/00509, 17/00878, PI20/00113, PI20/00226, Acción Transversal de Cáncer), Xunta de Galicia (PGIDIT07PXIB9101209PR), Ministerio de Economia y Competitividad (SAF07-64873, SAF 2010-19273, SAF2014-54453R), Fundación Científica de la Asociación Española contra el Cáncer (GCB13131592CAST), Beca Grupo de Trabajo "Oncología" AEG (Asociación Española de Gastroenterología), Fundación Privada Olga Torres, FP7 CHIBCHA Consortium, Agència de Gestió d'Ajuts Universitaris i de Recerca (AGAUR, Generalitat de Catalunya, 2014SGR135, 2014SGR255, 2017SGR21, 2017SGR653), Catalan Tumour Bank Network (Pla Director d'Oncologia, Generalitat de Catalunya), PERIS (SLT002/16/00398, Generalitat de Catalunya), CERCA Programme (Generalitat de Catalunya) and COST Action BM1206 and CA17118. CIBERehd is funded by the Instituto de Salud Carlos III. ESTHER/VERDI. This work was supported by grants from the Baden-Württemberg Ministry of Science, Research and Arts and the German Cancer Aid. Harvard cohorts (HPFS, NHS, PHS): HPFS is

supported by the National Institutes of Health (P01 CA055075, UM1 CA167552, U01 CA167552, R01 CA137178, R01 CA151993, and R35 CA197735), NHS by the National Institutes of Health (R01 CA137178, P01 CA087969, UM1 CA186107, R01 CA151993, and R35 CA197735) and PHS by the National Institutes of Health (R01 CA042182). Hawaii Adenoma Study: NCI grants R01 CA72520. HCES-CRC: the Hwasun Cancer Epidemiology Study–Colon and Rectum Cancer (HCES-CRC; grants from Chonnam National University Hwasun Hospital, HCRI15011-1). Kentucky: This work was supported by the following grant support: Clinical Investigator Award from Damon Runyon Cancer Research Foundation (CI-8); NCI R01CA136726. LCCS: The Leeds Colorectal Cancer Study was funded by the Food Standards Agency and Cancer Research UK Programme Award (C588/A19167). MCCS cohort recruitment was funded by VicHealth and Cancer Council Victoria. The MCCS was further supported by Australian NHMRC grants 509348, 209057, 251553 and 504711 and by infrastructure provided by Cancer Council Victoria. Cases and their vital status were ascertained through the Victorian Cancer Registry (VCR) and the Australian Institute of Health and Welfare (AIHW), including the National Death Index and the Australian Cancer Database. MEC: National Institutes of Health (R37 CA54281, P01 CA033619, and R01 CA063464). MECC: This work was supported by the National Institutes of Health, U.S. Department of Health and Human Services (R01 CA81488, R01 CA197350). MSKCC: The work at Sloan Kettering in New York was supported by the Robert and Kate Niehaus Center for Inherited Cancer Genomics and the Romeo Milio Foundation. Moffitt: This work was supported by funding from the National Institutes of Health (grant numbers R01 CA189184, P30 CA076292), Florida Department of Health Bankhead-Coley Grant 09BN-13, and the University of South Florida Oehler Foundation. Moffitt contributions were supported in part by the Total Cancer Care Initiative, Collaborative Data Services Core, and Tissue Core at the H. Lee Moffitt Cancer Center & Research Institute, a National Cancer Institute-designated Comprehensive Cancer Center (grant number P30 CA076292). NCCCS I & II: We acknowledge funding support for this project from the National Institutes of Health, R01 CA66635 and P30 DK034987. NFCCR: This work was supported by an Interdisciplinary Health Research Team award from the Canadian Institutes of Health Research (CRT 43821); the National Institutes of Health, U.S. Department of Health and Human Serivces (U01 CA74783); and National Cancer Institute of Canada grants (18223 and 18226). The authors wish to acknowledge the contribution of Alexandre Belisle and the genotyping team of the McGill University and Génome Québec Innovation Centre, Montréal, Canada, for genotyping the Sequenom panel in the NFCCR samples. Funding was provided to Michael O. Woods by the Canadian Cancer Society Research Institute. NSHDS: The research was supported by Biobank Sweden through funding from the Swedish Research Council (VR 2017-00650, VR 2017-01737), the Swedish Cancer Society (CAN 2017/581), Region Västerbotten (VLL-841671, VLL-833291), Knut and Alice Wallenberg Foundation (VLL-765961), and the Lion's Cancer Research Foundation (several grants) and Insamlingsstiftelsen, both at Umeå University. OFCCR: The Ontario Familial Colorectal Cancer Registry was supported in part by the National Cancer Institute (NCI) of the National Institutes of Health (NIH) under award U01 CA167551 and award U01/U24 CA074783 (to SG). Additional funding for the OFCCR and ARCTIC testing and genetic analysis was through and a Canadian Cancer Society CaRE (Cancer Risk Evaluation) program grant and Ontario Research Fund award GL201-043 (to BWZ), through the Canadian Institutes of Health Research award 112746 (to TJH), and through generous support from the Ontario Ministry of Research and Innovation. OSUMC: OCCPI funding was provided by Pelotonia and HNPCC funding was provided by the NCI (CA16058 and CA67941). PLCO: Intramural Research Program of the Division of Cancer Epidemiology and Genetics and supported by contracts from the Division of Cancer Prevention, National Cancer Institute, NIH, DHHS. Funding was provided by National Institutes of Health (NIH), Genes, Environment and Health Initiative (GEI) Z01 CP 010200, NIH U01 HG004446, and NIH GEI U01 HG 004438. SCCFR: The Seattle Colon Cancer Family Registry was supported in part by the National Cancer Institute (NCI) of the National Institutes of Health (NIH) under awards U01 CA167551, U01 CA074794

(to JDP), and awards U24 CA074794 and R01 CA076366 (to PAN). SEARCH: The University of Cambridge has received salary support in respect of PDPP from the NHS in the East of England through the Clinical Academic Reserve. Cancer Research UK (C490/A16561); the UK National Institute for Health Research Biomedical Research Centres at the University of Cambridge. SELECT: Research reported in this publication was supported in part by the National Cancer Institute of the National Institutes of Health under Award Numbers U10 CA37429 (CD Blanke), and UM1 CA182883 (CM Tangen/IM Thompson). The content is solely the responsibility of the authors and does not necessarily represent the official views of the National Institutes of Health. SMS and REACH: This work was supported by the National Cancer Institute (grant P01 CA074184 to J.D.P. and P.A.N., grants R01 CA097325, R03 CA153323, and K05 CA152715 to P.A.N., and the National Center for Advancing Translational Sciences at the National Institutes of Health (grant KL2 TR000421 to A.N.B.-H.) The Swedish Low-risk Colorectal Cancer Study: The study was supported by grants from the Swedish research council; K2015-55X-22674-01-4, K2008-55X-20157-03-3, K2006-72X-20157-01-2 and the Stockholm County Council (ALF project). Swedish Mammography Cohort and Cohort of Swedish Men: This work is supported by the Swedish Research Council /Infrastructure grant, the Swedish Cancer Foundation, and the Karolinska Institute´s Distinguished Professor Award to Alicja Wolk. UK Biobank: This research has been conducted using the UK Biobank Resource under Application Number 8614. VITAL: National Institutes of Health (K05 CA154337). WHI: The WHI program is funded by the National Heart, Lung, and Blood Institute, National Institutes of Health, U.S. Department of Health and Human Services through contracts HHSN268201100046C, HHSN268201100001C, HHSN268201100002C, HHSN268201100003C, HHSN268201100004C, and HHSN271201100004C.

GICC: The Glioma International Case Control Consortium (GICC) was supported by grants from the US National Institutes of Health (NIH) (R01 CA139020)

Head neck cancer GWAS consortium: Genotyping of cases and controls included was performed at the Center for Inherited Disease Research (CIDR) and funded by the US National Institute of Dental and Craniofacial Research (NIDCR; 1X01HG007780-0). Genotyping for shared controls with the Lung OncoArray initiative was funded through grant X01HG007492-0.

Head neck cancer GWAS individual studies: **ARCAGE:** The Alcohol-Related Cancers and Genetic Susceptibility Study in Europe (ARCAGE) was funded by the European Commission's fifth framework programme (QLK1-2001-00182), the Italian Association for Cancer Research, Compagnia di San Paolo/FIRMS, Region Piemonte and Padova University (CPDA057222). **CHANCE:** The Carolina Head and Neck Cancer Study (CHANCE) was supported by the National Cancer Institute (R01CA90731). **HN5000:** The HN5000 study was funded by the National Institute for Health Research (NIHR) under its Programme Grants for Applied Research scheme (RP-PG-0707-10034); the views expressed in this publication are those of the author(s) and not necessarily those of the NHS, the NIHR or the UK Department of Health. Core funding was also provided through awards from Above and Beyond, University Hospitals Bristol Research Capability Funding and the NIHR Senior Investigator award to Professor Andy Ness. Human papillomavirus (HPV) serology was supported by a Cancer Research UK Programme Grant, the Integrative Cancer Epidemiology Programme (grant number: C18281/A19169). **Pitt:** The University of Pittsburgh head and neck cancer case–control study is supported by US National Institutes of Health grants P50CA097190 and P30CA047904. **Toronto:** The Toronto study was funded by the Canadian Cancer Society Research Institute (020214) and the National Cancer Institute (U19CA148127) and by the Cancer Care Ontario Research Chair. **EPIC:** Coordination of the EPIC study is financially supported by the European Commission (DG SANCO) and the International Agency for Research on Cancer. **IARC Oral Cancer:** The IARC Oral Cancer Multicenter study was funded by grant S06 96 202489 05F02 from Europe

against Cancer; grants FIS 97/0024, FIS 97/0662 and BAE 01/5013 from Fondo de Investigaciones Sanitarias, Spain; the UICC Yamagiwa-Yoshida Memorial International Cancer Study; the National Cancer Institute of Canada; Associazione Italiana per la Ricerca sul Cancro; and the Pan-American Health Organization. **IARC Central Europe:** The IARC Central Europe study was supported by the European Commission's INCO-COPERNICUS Program (IC15-CT98-0332), US NIH/National Cancer Institute grant CA92039 and World Cancer Research Foundation grant WCRF 99A28. **Rome:** The Rome Study was supported by the Associazione Italiana per la Ricerca sul Cancro (AIRC) awards IG 2011 10491 and IG 2013 14220 to Stefania Boccia and by Fondazione Veronesi to Stefania Boccia. **GENCAPO:** Genome Project (GENCAPO) was supported by the Fundação de Amparo à Pesquisa do Estado de São Paulo (FAPESP; grants 04/12054-9 and 10/51168-0). **IARC Latin American study:** The IARC Latin American study was funded by the European Commission INCO-DC programme (IC18-CT97-0222), with additional funding from Fondo para la Investigación Científica y Tecnológica (Argentina) and the Fundação de Amparo à Pesquisa do Estado de São Paulo (01/01768-2).

ILCCO: Lung cancer GWAS from the International Lung Cancer Consortium (ILCCO) was supported by NIH U19 CA203654 and U19 CA148127 and the data harmonization was supported by Canada Research Chair to R. J. H. This work has been supported by the Intramural Research Program (IRP) of the Division of Cancer Epidemiology and Genetics, National Cancer Institute, US National Institutes of Health. The content of this publication does not necessarily reflect the views or policies of the US Department of Health and Human Services, nor does mention of trade names, commercial products, or organizations imply endorsement by the US government.

Melanoma GWAS Consortium: Please see reference 43.

PanScan: The authors acknowledge the research contributions of the Cancer Genomics Research Laboratory, Division of Cancer Epidemiology and Genetics, National Cancer Institute (NCI), National Institutes of Health (NIH) for their expertise, execution, and support of this research in the areas of project planning, wet laboratory processing of specimens, and bioinformatics analysis of generated data. This project has been funded in whole or in part with Federal funds from the NCI, NIH, under NCI Contract No. 75N910D00024. The content of this publication does not necessarily reflect the views or policies of the Department of Health and Human Services, nor does mention of trade names, commercial products, or organizations imply endorsement by the U.S. Government. The American Cancer Society (ACS) funds the creation, maintenance, and updating of the Cancer Prevention Study II cohort. Cancer incidence data for CLUE were provided by the Maryland Cancer Registry, Center for Cancer Surveillance and Control, Department of Health and Mental Hygiene, 201 W. Preston Street, Room 400, Baltimore, MD 21201, http://phpa.dhmh.maryland.gov/cancer, 410-767-4055. We acknowledge the State of Maryland, the Maryland Cigarette Restitution Fund, and the National Program of Cancer Registries of the Centers for Disease Control and Prevention for the funds that support the collection and availability of the cancer registry data." We thank all the CLUE participants. The coordination of EPIC is financially supported by International Agency for Research on Cancer (IARC) and by the Department of Epidemiology and Biostatistics, School of Public Health, Imperial College London which has additional infrastructure support provided by the NIHR Imperial Biomedical Research Centre (BRC). The Health Professionals Follow-up Study is supported by NIH grant UM1 CA167552. from the National Cancer Institute, Bethesda, MD USA. The Melbourne Collaborative Cohort Study (MCCS) cohort recruitment was funded by VicHealth and Cancer Council Victoria. The MCCS was further augmented by Australian National Health and Medical Research Council grants 209057, 396414 and 1074383 and by infrastructure provided by Cancer Council Victoria. Cases and their vital status were ascertained through the Victorian Cancer Registry and the Australian Institute of Health and Welfare, including the National Death Index and the Australian Cancer

Database. Nurses' Health Study is supported by NIH grants UM1 CA186107, P01 CA87969, and R01 CA49449 from the National Cancer Institute, Bethesda, MD USA. The NYU study (AZJ and AAA) was funded by NIH R01 CA098661, UM1 CA182934 and center grants P30 CA016087 and P30 ES000260. The PANKRAS II Study in Spain was supported by research grants from Instituto de Salud Carlos III-FEDER, Spain: Fondo de Investigaciones Sanitarias (FIS) (#PI13/00082 and #PI15/01573) and Red Temática de Investigación Cooperativa en Cáncer, Spain (#RD12/0036/0050); and European Cooperation in Science and Technology (COST Action #BM1204: EU_Pancreas), Ministerio de Ciencia y Tecnología (CICYT SAF 2000-0097), Fondo de Investigación Sanitaria (95/0017), Madrid, Spain; Generalitat de Catalunya (CIRIT - SGR); 'Red temática de investigación cooperativa de centros en Cáncer' (C03/10), 'Red temática de investigación cooperativa de centros en Epidemiología y salud pública' (C03/09), and CIBER de Epidemiología (CIBERESP), Madrid. The Physicians' Health Study was supported by research grants CA-097193, CA-34944, CA-40360, HL-26490, and HL-34595 from the National Institutes of Health, Bethesda, MD USA. The SELECT study is supported by National Institutes of Health grant award number U10 CA37429 (CD Blanke), and UM1 CA182883 (CM Tangen/IM Thompson). The authors thank the site investigators and staff and, most importantly, the participants from PCPT and SELECT who donated their time to this trial. The Shanghai Men's Health Study is supported by NIH grant UM1CA173640. The Shanghai Women's Health Study is supported by NIH grant UM1CA182910. The Women's Health Study was supported by research grants CA182913, CA-047988, HL-043851, HL-080467, and HL-099355 from the National Institutes of Health, Bethesda, MD USA. The WHI program is funded by the National Heart, Lung, and Blood Institute, National Institutes of Health, U.S. Department of Health and Human Services through contracts HHSN268201600018C, HHSN268201600001C, HHSN268201600002C, HHSN268201600003C, and HHSN268201600004C. The authors thank the WHI investigators and staff for their dedication, and the study participants for making the program possible. A full listing of WHI investigators can be found at: http://www.whi.org/researchers/Documents%20%20Write%20a%20Paper/WHI%20Investigator%20Long%20List.pdf.

<u>PAN4C:</u> This PANC4 GWAS was supported by RO1CA154823 and P50CA062924 (PI: A.P. Klein). The IARC/Central Europe study was supported by a grant from the US National Cancer Institute at the National Institutes of Health (R03 CA123546-02) and grants from the Ministry of Health of the Czech Republic (NR 9029-4/2006, NR9422-3, NR9998-3, MH CZ-DRO-MMCI 00209805). The work at Johns Hopkins University was supported by the NCI Grants P50CA062924 and R01CA97075. Additional support was provided by the Lustgarten Foundation, Susan Wojcicki and Dennis Troper and the Sol Goldman Pancreas Cancer Research Center. This work was supported by RO1 CA154823 and federal funds from the National Cancer Institute (NCI), US National Institutes of Health (NIH) under contract number HHSN261200800001E. The Mayo Clinic Biospecimen Resource for Pancreas Research study is supported by the Mayo Clinic SPORE in Pancreatic Cancer (P50 CA102701). The Memorial Sloan Kettering Cancer Center Pancreatic Tumor Registry is supported by P30CA008748, the Geoffrey Beene Foundation, the Arnold and Arlene Goldstein Family Foundation, and the Society of MSKCC. We acknowledge the contribution of Dr. Irene Orlow, MS, PhD to this analysis. The PACIFIC Study was supported by RO1CA102765, Kaiser Permanente and Group Health Cooperative. The Queensland Pancreatic Cancer Study was supported by a grant from the National Health and Medical Research Council of Australia (NHMRC) (Grant number 442302). RE Neale is supported by a NHMRC Senior Research Fellowship (#1060183). The UCSF pancreas study was supported by NIH-NCI grants (R01CA1009767, R01CA109767-S1 and R0CA059706) and the Joan Rombauer Pancreatic Cancer Fund. Collection of cancer incidence data was supported by the California Department of Public Health as part of the statewide cancer reporting program; the NCI's SEER Program under contract HSN261201000140C awarded to CPIC; and the CDC's National Program of Cancer Registries, under agreement #U58DP003862-01 awarded to

the California Department of Public Health. The Yale (CT) pancreas cancer study is supported by National Cancer Institute at the U.S. National Institutes of Health, grant 5R01CA098870. The cooperation of 30 Connecticut hospitals, including Stamford Hospital, in allowing patient access, is gratefully acknowledged. The Connecticut Pancreas Cancer Study was approved by the State of Connecticut Department of Public Health Human Investigation Committee. Certain data used in that study were obtained from the Connecticut Tumor Registry in the Connecticut Department of Public Health. The authors assume full responsibility for analyses and interpretation of these data.

OCAC: The Ovarian Cancer Association Consortium is supported by a grant from the Ovarian Cancer Research Fund thanks to donations by the family and friends of Kathryn Sladek Smith (PPD/RPCI.07). The scientific development and funding for this project were in part supported by the US National Cancer Institute GAME-ON Post-GWAS Initiative (U19-CA148112). This study made use of data generated by the Wellcome Trust Case Control consortium that was funded by the Wellcome Trust under award 076113. The results published here are in part based upon data generated by The Cancer Genome Atlas Pilot Project established by the National Cancer Institute and National Human Genome Research Institute (dbGap accession number phs000178.v8.p7). The OCAC OncoArray genotyping project was funded through grants from the U.S. National Institutes of Health (CA1X01HG007491-01 (C.I.A.), U19-CA148112 (T.A.S.), R01-CA149429 (C.M.P.) and R01-CA058598 (M.T.G.); Canadian Institutes of Health Research (MOP-86727 (L.E.K.) and the Ovarian Cancer Research Fund (A.B.). The COGS project was funded through a European Commission's Seventh Framework Programme grant (agreement number 223175 - HEALTH-F2-2009-223175).

OCAC Individual Studies: **AAS**: National Institutes of Health (RO1-CA142081); **AUS**: The Australian Ovarian Cancer Study (AOCS) was supported by the U.S. Army Medical Research and Materiel Command (DAMD17-01-1-0729), National Health & Medical Research Council of Australia (199600, 400413 and 400281), Cancer Councils of New South Wales, Victoria, Queensland, South Australia and Tasmania and Cancer Foundation of Western Australia (Multi-State Applications 191, 211 and 182). AOCS gratefully acknowledges additional support from Ovarian Cancer Australia and the Peter MacCallum Foundation; **BAV**: ELAN Funds of the University of Erlangen-Nuremberg; **BEL**: National Kankerplan; **BGS**: Breast Cancer Now, Institute of Cancer Research; **BVU**: Vanderbilt University Medical Center’s BioVU is supported by the 1S10RR025141-01 instrumentation award and Vanderbilt CTSA grant from the National Institutes of Health (NIH)/National Center for Advancing Translational Sciences (NCATS) (ULTR000445); **CAM**: National Institutes of Health Research Cambridge Biomedical Research Centre and Cancer Research UK Cambridge Cancer Centre; **CHA**: Innovative Research Team in University (PCSIRT) in China (IRT1076); **CNI**: Instituto de Salud Carlos III (PI 12/01319); Ministerio de Economía y Competitividad (SAF2012); **DKE**: Ovarian Cancer Research Fund; **DOV**: National Institutes of Health R01-CA112523 and R01-CA87538; **EPC**: The coordination of EPIC is financially supported by the European Commission (DG-SANCO) and the International Agency for Research on Cancer. The national cohorts are supported by Danish Cancer Society (Denmark) (EMC 2014-6699); Ligue Contre le Cancer, Institut Gustave Roussy, Mutuelle Générale de l’Education Nationale, Institut National de la Santé et de la Recherche Médicale (INSERM) (France); German Cancer Aid, German Cancer Research Center (DKFZ), Federal Ministry of Education and Research (BMBF) (Germany); the Hellenic Health Foundation (Greece); Associazione Italiana per la Ricerca sul Cancro-AIRC-Italy and National Research Council (Italy); Dutch Ministry of Public Health, Welfare and Sports (VWS), Netherlands Cancer Registry (NKR), LK Research Funds, Dutch Prevention Funds, Dutch ZON (Zorg Onderzoek Nederland), World Cancer Research Fund (WCRF), Statistics Netherlands (The Netherlands); ERC-2009-AdG 232997 and Nordforsk, Nordic Centre of Excellence programme on Food, Nutrition and Health (Norway); Health Research Fund (FIS), PI13/00061 to Granada, PI13/01162 to EPIC-Murcia, Regional Governments of Andalucía, Asturias, Basque Country,

Murcia and Navarra, ISCIII RETIC (RD06/0020) (Spain); Swedish Cancer Society, Swedish Research Council and County Councils of Skåne and Västerbotten (Sweden); Cancer Research UK (14136 to EPIC-Norfolk; C570/A16491 and C8221/A19170 to EPIC-Oxford), Medical Research Council (1000143 to EPIC-Norfolk, MR/M012190/1 to EPIC-Oxford) (United Kingdom); **GER**: German Federal Ministry of Education and Research, Programme of Clinical Biomedical Research (01 GB 9401) and the German Cancer Research Center (DKFZ); **GRC**: This research has been co-financed by the European Union (European Social Fund - ESF) and Greek national funds through the Operational Program "Education and Lifelong Learning" of the National Strategic Reference Framework (NSRF) - Research Funding Program of the General Secretariat for Research & Technology: SYN11_10_19 NBCA. Investing in knowledge society through the European Social Fund; **GRR**: Roswell Park Cancer Institute Alliance Foundation, P30 CA016056; HAW: U.S. National Institutes of Health (R01-CA58598, N01-CN-55424 and N01-PC-67001); **HJO**: Intramural funding; Rudolf-Bartling Foundation; **HMO**: Intramural funding; Rudolf-Bartling Foundation; HOC: Helsinki University Hospital Research Fund; **HOP**: University of Pittsburgh School of Medicine Dean’s Faculty Advancement Award (F. Modugno), Department of Defense (DAMD17-02-1-0669) and NCI (K07-CA080668, R01-CA95023, P50-CA159981 MO1-RR000056 R01-CA126841); **HUO**: Intramural funding; Rudolf-Bartling Foundation; JPN: Grant-in-Aid for the Third Term Comprehensive 10-Year Strategy for Cancer Control from the Ministry of Health, Labour and Welfare; **KRA**: This study (Ko-EVE) was supported by a grant from the Korea Health Technology R&D Project through the Korea Health Industry Development Institute (KHIDI), and the National R&D Program for Cancer Control, Ministry of Health & Welfare, Republic of Korea (HI16C1127; 0920010); **LAX**: American Cancer Society Early Detection Professorship (SIOP-06-258-01-COUN) and the National Center for Advancing Translational Sciences (NCATS), Grant UL1TR000124; **LUN**: ERC-2011-AdG 294576-risk factors cancer, Swedish Cancer Society, Swedish Research Council, Beta Kamprad Foundation; **MAC**: National Institutes of Health (R01-CA122443, P30-CA15083, P50-CA136393); Mayo Foundation; Minnesota Ovarian Cancer Alliance; Fred C. and Katherine B. Andersen Foundation; Fraternal Order of Eagles; **MAL**: Funding for this study was provided by research grant R01- CA61107 from the National Cancer Institute, Bethesda, MD, research grant 94 222 52 from the Danish Cancer Society, Copenhagen, Denmark; and the Mermaid I project; **MAS**: Malaysian Ministry of Higher Education (UM.C/HlR/MOHE/06) and Cancer Research Initiatives Foundation; **MAY**: National Institutes of Health (R01-CA122443, P30-CA15083, P50-CA136393); Mayo Foundation; Minnesota Ovarian Cancer Alliance; Fred C. and Katherine B. Andersen Foundation; **MCC**: MCCS cohort recruitment was funded by VicHealth and Cancer Council Victoria. Cancer Council Victoria, National Health and Medical Research Council of Australia (NHMRC) grants number 209057, 251533, 396414, and 504715; **MDA**: DOD Ovarian Cancer Research Program (W81XWH-07-0449); MEC: NIH (CA54281, CA164973, CA63464); **MOF**: Moffitt Cancer Center, Merck Pharmaceuticals, the state of Florida, Hillsborough County, and the city of Tampa; **NCO**: National Institutes of Health (R01-CA76016) and the Department of Defense (DAMD17-02-1-0666); **NEC**: National Institutes of Health R01-CA54419 and P50-CA105009 and Department of Defense W81XWH-10-1-02802; NHS: UM1 CA186107, P01 CA87969, R01 CA49449, R01-CA67262, UM1 CA176726; NJO: National Cancer Institute (NIH-K07 CA095666, R01-CA83918, NIH-K22-CA138563, and P30-CA072720) and the Cancer Institute of New Jersey; If Sara Olson and/or Irene Orlow is a co-author, please add NCI CCSG award (P30-CA008748) to the funding sources; **NOR**: Helse Vest, The Norwegian Cancer Society, The Research Council of Norway; **NTH**: Radboud University Medical Centre; **OPL**: National Health and Medical Research Council (NHMRC) of Australia (APP1025142) and Brisbane Women's Club; **ORE**: Sherie Hildreth Ovarian Cancer (SHOC) Foundation; **OVA**: This work was supported by Canadian Institutes of Health Research grant (MOP-86727) and by NIH/NCI 1 R01CA160669-01A1; **PLC**: Intramural Research Program of the National Cancer Institute; **POC**: Pomeranian Medical University; **POL**: Intramural Research Program of the National Cancer Institute; **PVD**: Canadian Cancer Society and Cancer

Research Society GRePEC Program; **RBH**: National Health and Medical Research Council of Australia; **RMH**: Cancer Research UK, Royal Marsden Hospital; **RPC**: National Institute of Health (P50 CA159981, R01CA126841); **SEA**: Cancer Research UK (C490/A10119 C490/A10124); UK National Institute for Health Research Biomedical Research Centres at the University of Cambridge; **SIS**: The Sister Study (SISTER) is supported by the Intramural Research Program of the NIH, National Institute of Environmental Health Sciences (Z01-ES044005 and Z01-ES049033); **SMC**: The Swedish Cancer Foundation and the Swedish Research Council (VR 2017-00644) grant for the Swedish Infrastructure for Medical Population-based Life-course Environmental Research (SIMPLER); **SRO**: Cancer Research UK (C536/A13086, C536/A6689) and Imperial Experimental Cancer Research Centre (C1312/A15589); **STA**: NIH grants U01 CA71966 and U01 CA69417; **SWH**: NIH (NCI) grant R37-CA070867; **TBO**: National Institutes of Health (R01-CA106414-A2), American Cancer Society (CRTG-00-196-01-CCE), Department of Defense (DAMD17-98-1-8659), Celma Mastry Ovarian Cancer Foundation; **TOR**: NIH grants R01 CA063678 and R01 CA063682; **UCI**: NIH R01-CA058860 and the Lon V Smith Foundation grant LVS-39420; **UHN**: Princess Margaret Cancer Centre Foundation-Bridge for the Cure; **UKO**: The UKOPS study was funded by The Eve Appeal (The Oak Foundation) with investigators supported by the National Institute for Health Research University College London Hospitals Biomedical Research Centre and MRC core funding (MR_UU_12023); **UKR**: Cancer Research UK (C490/A6187), UK National Institute for Health Research Biomedical Research Centres at the University of Cambridge; **USC**: P01CA17054, P30CA14089, R01CA61132, N01PC67010, R03CA113148, R03CA115195, N01CN025403, and California Cancer Research Program (00-01389V-20170, 2II0200); **VAN**: BC Cancer Foundation, VGH & UBC Hospital Foundation; **VTL**: NIH K05-CA154337; WMH: National Health and Medical Research Council of Australia, Enabling Grants ID 310670 & ID 628903. Cancer Institute NSW Grants 12/RIG/1-17 & 15/RIG/1-16; **WOC**: National Science Centre (N N301 5645 40), The Maria Sklodowska-Curie Memorial Cancer Centre and Institute of Oncology, Warsaw, Poland.

<u>PEGASUS</u>: PEGASUS was supported by the Intramural Research Program, Division of Cancer Epidemiology and Genetics, National Cancer Institute, National Institutes of Health.

<u>Renal Cancer GWAS Studies:</u> **AHS**: This research was supported by the Intramural Research Program of the NIH, National Cancer Institute, Division of Cancer Epidemiology and Genetics (Z01CP010119). **ATBC**: The ATBC Study was supported by funding provided by the Intramural Research Program of the NCI, NIH, and through U.S. Public Health Service contracts (N01-CN-45165, N01-RC-45035 and N01-RC-37004) from the NCI. **BioVU**: The dataset used in the analyses described were obtained from the Vanderbilt University Medical Center resource BioVU, which is supported by institutional funding, the 1S10RR025141-01 instrumentation award, and by the Vanderbilt CTSA grant UL1 TR000445 from NCATS/NIH. Sample processing and phenotyping algorithm development was supported by institutional funding for TLE. **Center 'Bioengineering' of the Russian Academy of Sciences/Kurchatov Scientific Center**: The work conducted for this study was supported by the grant Russian Scientific Fund 14-14-01202. **Centre National de Genotypage, France**: We thank Jean Guillaume Garnier and Delphine Bacq-Daian for their work on the IARC-2 scan. **ConFIRM/MCCS**: The ConFIRM study, also known as CARES, was supported by the Victorian Cancer Agency (PTCB08_05), the Australian National Health and Medical Research Council (Project Grant 1011626). We acknowledge the contribution of Professor Graham Giles in supporting this work and of Ms Olive Schmid and Ms Jennifer Walsh for the project management. The Melbourne Collaborative Cohort Study (MCCS) recruitment was funded by VicHealth and Cancer Council Victoria. The MCCS was further supported by Australian NHMRC grants 209057, 251553 and 504711 and by infrastructure provided by Cancer Council Victoria. **CPS-II**: The Cancer Prevention Study II Nutrition Cohort is supported by the American Cancer Society. The authors thank all of the men and women in the Cancer Prevention Study II Nutrition Cohort for their many years of dedicated participation in the study.

Health Professionals Follow-up Study (HPFS) and Nurses' Health Study (NHS): The HPFS is supported by National Institutes of Health, National Cancer Institute grant UM1 CA167552. The NHS is supported by National Institutes of Health, National Cancer Institute grants UM1 CA186107 and P01 CA87969. We would like to thank the following state cancer registries for their help: AL, AZ, AR, CA, CO, CT, DE, FL, GA, ID, IL, IN, IA, KY, LA, ME, MD, MA, MI, NE, NH, NJ, NY, NC, ND, OH, OK, OR, PA, RI, SC, TN, TX, VA, WA, WY. The authors assume full responsibility for analyses and interpretation of these data. **K2 study**: This study was supported by the EU FP7 under grant agreement number 241669 (the CAGEKID project). In Czech Republic, this work was also supported by MH CZ—DRO (MMCI, 00209805) and by the project MEYS – NPS I – LO1413, Czech Republic. **Leeds Cohort**: The infrastructure support from Cancer Research UK as part of the Leeds Centre and Experimental Cancer Medicine Centre funding is gratefully acknowledged together with the Leeds Multidisciplinary Research Tissue Bank, all patients who consented to take part in the research studies and the staff of the Urology and Oncology Departments in Leeds Teaching Hospitals Trust. **Mayo Clinic**: This study was partially supported by National Institutes of Health: R21CA176422 (JEEP) and R01CA134466 (ASP). The authors acknowledge the Mayo Clinic Comprehensive Cancer Center Biospecimens Accessioning and Processing Shared Resource and the Pathology Research Core Shared Resource. **MD Anderson**: This work was supported in part by the NIH (grant R01 CA170298) and the Center for Translational and Public Health Genomics, Duncan Family Institute for Cancer Prevention and Risk Assessment, The University of Texas MD Anderson Cancer Center. **NCI/IARC RCC Study in Central Europe (CE)**: This project was supported by the Intramural Research Program of the NIH and the National Cancer Institute. **Physicians' Health Study (PHS)**: This study was supported by grants CA 097193, CA 34944, CA 40360, HL 26490, and HL 34595 from the National Institutes of Health, Bethesda, MD. **PLCO**: This research was supported by the Intramural Research Program of the National Cancer Institute and by contracts from the Division of Cancer Prevention, National Cancer Institute, NIH, DHHS. **SEARCH**: This study is funded by Cancer Research UK (C490/A16651). **UK GWAS**: We acknowledge support from the Medical Research Council (MRC), Cancer Research UK, an educational grant from Bayer and NHS funding for the Royal Marsden Biomedical Research Centre and Cambridge University Health Partners. JL is supported by the NIHR RM/CR Biomedical Research Centre for Cancer. **US Kidney Cancer Study**: The NCI United Stated Kidney Cancer Study was supported by the Intramural Research Program of the National Institutes of Health and the National Cancer Institute under the following contracts: N02-CP-10128 (Westat, Inc.), N02-CP-11004 (Wayne State University), and N02-CP- 11161 (University of Illinois at Chicago). **Van Andel Research Institute (VARI)**: The authors would like to thank Dr. Kyle Furge for his role in this project as well as Drs. Anthony Avallone, John Ludlow and Philip Wise for contributing samples for this project. **Women's Health Initiative (WHI)**: The WHI program is funded by the National Heart, Lung, and Blood Institute, National Institutes of Health, U.S. Department of Health and Human Services through contracts HHSN268201100046C, HHSN268201100001C, HHSN268201100002C, HHSN268201100003C, HHSN268201100004C, and HHSN271201100004C. For a list of all the investigators who have contributed to WHI science, please visit: https://www.whi.org/researchers/Documents%20%20Write%20a%20Paper/WHI%20Investigator %20Long%20List.pdf". **Women's Health Study (WHS)**: The study is supported by grants CA-047988, HL-043851, HL-080467, HL-099355, and UM1CA182913 from the National Institutes of Health, Bethesda, MD.

S. M. is supported by an Australian National Health and Medical Research Fellowship.

T.O. M and A.B. S are supported by National Health and Medical Research Council of Australia Investigator Fellowships (APP1173170 & APP177524).

Acknowledgement

The internal LD reference panel used in the COJO-ASSET and PAINTOR analyses were based on breast cancer OncoArray data accessed through dbGap (accession number phs001265). OncoArray genotyping and phenotype data harmonization for the Discovery, Biology, and Risk of Inherited Variants in Breast Cancer (DRIVE) breast-cancer case control samples was supported by X01 HG007491 and U19 CA148065 and by Cancer Research UK (C1287/A16563). Genotyping was conducted by the Center for Inherited Disease Research (CIDR), Centre for Cancer Genetic Epidemiology, University of Cambridge, and the National Cancer Institute. The following studies contributed germline DNA from breast cancer cases and controls: the Two Sister Study (2SISTER), Breast Oncology Galicia Network (BREOGAN), Copenhagen General Population Study (CGPS), Cancer Prevention Study 2 (CPSII), The European Prospective Investigation into Cancer and Nutrition (EPIC), Melbourne Collaborative Cohort Study (MCCS), Multiethnic Cohort (MEC), Nashville Breast Health Study (NBHS), Nurses Health Study (NHS), Nurses Health Study 2 (NHS2), Polish Breast Cancer Study (PBCS), Prostate Lung Colorectal and Ovarian Cancer Screening Trial (PLCO), Studies of Epidemiology and Risk Factors in Cancer Heredity (SEARCH), The Sister Study (SISTER), Swedish Mammographic Cohort (SMC), Women of African Ancestry Breast Cancer Study (WAABCS), Women's Health Initiative (WHI).

From BCAC: We thank all the individuals who took part in these studies and all the researchers, clinicians, technicians and administrative staff who have enabled this work to be carried out. The COGS study would not have been possible without the contributions of the following: Craig Luccarini and the staff of the Centre for Genetic Epidemiology Laboratory, Anna Gonzalez-Neira and the staff of the CNIO genotyping unit, Daniel C. Tessier, Francois Bacot, Daniel Vincent, Sylvie LaBoissière, Frederic Robidoux and the staff of the McGill University and Génome Québec Innovation Centre, Sune F. Nielsen, Borge G. Nordestgaard, and the staff of the Copenhagen DNA laboratory, and, Julie M. Cunningham, Sharon A. Windebank, Christopher A. Hilker, Jeffrey Meyer and the staff of Mayo Clinic Genotyping Core Facility. ABCFS thank Maggie Angelakos, Judi Maskiell, Gillian Dite. ABCS thanks the Blood bank Sanquin, The Netherlands. ABCTB Investigators: Christine Clarke, Deborah Marsh, Rodney Scott, Robert Baxter, Desmond Yip, Jane Carpenter, Alison Davis, Nirmala Pathmanathan, Peter Simpson, J. Dinny Graham, Mythily Sachchithananthan. Samples are made available to researchers on a non-exclusive basis. The ACP study wishes to thank the participants in the Thai Breast Cancer study. Special Thanks also go to the Thai Ministry of Public Health (MOPH), doctors and nurses who helped with the data collection process. Finally, the study would like to thank Dr Prat Boonyawongviroj, the former Permanent Secretary of MOPH and Dr Pornthep Siriwanarungsan, the former Department Director-General of Disease Control who have supported the study throughout. BBCS thanks Eileen Williams, Elaine Ryder-Mills, Kara Sargus. BCEES thanks Allyson Thomson, Christobel Saunders, Terry Slevin, BreastScreen Western Australia, Elizabeth Wylie, Rachel Lloyd. The BCINIS study would not have been possible without the contributions of Dr. K. Landsman, Dr. N. Gronich, Dr. A. Flugelman, Dr. W. Saliba, Dr. F. Lejbkowicz, Dr. E. Liani, Dr. I. Cohen, Dr. S. Kalet, Dr. V. Friedman, Dr. O. Barnet of the NICCC in Haifa, and all the contributing family medicine, surgery, pathology and oncology teams in all medical institutes in Northern Israel. BIGGS thanks Niall McInerney, Gabrielle Colleran, Andrew Rowan, Angela Jones. The BREOGAN study would not have been possible without the contributions of the following: Manuela Gago-Dominguez, Jose Esteban Castelao, Angel Carracedo, Victor Muñoz Garzón, Alejandro Novo Domínguez, Maria Elena Martinez, Sara Miranda Ponte, Carmen Redondo Marey, Maite Peña Fernández, Manuel Enguix Castelo, Maria Torres, Manuel Calaza (BREOGAN), José Antúnez, Máximo Fraga and the staff of the Department of Pathology and Biobank of the University Hospital Complex of Santiago-CHUS, Instituto de Investigación Sanitaria de Santiago, IDIS, Xerencia de Xestion Integrada de Santiago-SERGAS; Joaquín González-Carreró and the staff of the Department of Pathology and Biobank of University Hospital Complex of Vigo, Instituto de

Investigacion Biomedica Galicia Sur, SERGAS, Vigo, Spain. The BSUCH study thanks Peter Bugert, Medical Faculty Mannheim. CBCS thanks study participants, co-investigators, collaborators and staff of the Canadian Breast Cancer Study, and project coordinators Agnes Lai and Celine Morissette. CCGP thanks Styliani Apostolaki, Anna Margiolaki, Georgios Nintos, Maria Perraki, Georgia Saloustrou, Georgia Sevastaki, Konstantinos Pompodakis. CGPS thanks staff and participants of the Copenhagen General Population Study. For the excellent technical assistance: Dorthe Uldall Andersen, Maria Birna Arnadottir, Anne Bank, Dorthe Kjeldgård Hansen. The Danish Cancer Biobank is acknowledged for providing infrastructure for the collection of blood samples for the cases. CNIO-BCS thanks Guillermo Pita, Charo Alonso, Nuria Álvarez, Pilar Zamora, Primitiva Menendez, and the Human Genotyping-CEGEN Unit (CNIO). Investigators from the CPS-II cohort thank the participants and Study Management Group for their invaluable contributions to this research. They also acknowledge the contribution to this study from central cancer registries supported through the Centers for Disease Control and Prevention National Program of Cancer Registries, as well as cancer registries supported by the National Cancer Institute Surveillance Epidemiology and End Results program. The authors would like to thank the California Teachers Study Steering Committee that is responsible for the formation and maintenance of the Study within which this research was conducted. A full list of California Teachers Study team members is available at https://www.calteachersstudy.org/team. DIETCOMPLYF thanks the patients, nurses and clinical staff involved in the study. The DietCompLyf study was funded by the charity Against Breast Cancer (Registered Charity Number 1121258) and the NCRN. We thank the participants and the investigators of EPIC (European Prospective Investigation into Cancer and Nutrition). ESTHER thanks Hartwig Ziegler, Sonja Wolf, Volker Hermann, Christa Stegmaier, Katja Butterbach. GC-HBOC thanks Stefanie Engert, Heide Hellebrand, Sandra Kröber and LIFE - Leipzig Research Centre for Civilization Diseases (Markus Loeffler, Joachim Thiery, Matthias Nüchter, Ronny Baber). The GENICA Network: Dr. Margarete Fischer-Bosch-Institute of Clinical Pharmacology, Stuttgart, and University of Tübingen, Germany [H.B., Wing-Yee Lo], Department of Internal Medicine, Evangelische Kliniken Bonn gGmbH, Johanniter Krankenhaus, Bonn, Germany [Yon-Dschun Ko, Christian Baisch], Institute of Pathology, University of Bonn, Germany [Hans-Peter Fischer], Molecular Genetics of Breast Cancer, Deutsches Krebsforschungszentrum (DKFZ), Heidelberg, Germany [Ute Hamann], Institute for Prevention and Occupational Medicine of the German Social Accident Insurance, Institute of the Ruhr University Bochum (IPA), Bochum, Germany [Thomas Brüning, Beate Pesch, Sylvia Rabstein, Anne Lotz]; and Institute of Occupational Medicine and Maritime Medicine, University Medical Center Hamburg-Eppendorf, Germany [Volker Harth]. HABCS thanks Michael Bremer. HEBCS thanks Johanna Kiiski, Carl Blomqvist, Taru A. Muranen, Kristiina Aittomäki, Kirsimari Aaltonen, Karl von Smitten, Irja Erkkilä. HKBCS thanks Hong Kong Sanatorium and Hospital, Dr Ellen Li Charitable Foundation, The Kerry Group Kuok Foundation, National Institute of Health 1R03CA130065 and the North California Cancer Center for support. HMBCS thanks Peter Hillemanns, Hans Christiansen and Johann H. Karstens. HUBCS thanks Shamil Gantsev. KARMA and SASBAC thank the Swedish Medical Research Counsel. KBCP thanks Eija Myöhänen, Helena Kemiläinen. kConFab/AOCS wish to thank Heather Thorne, Eveline Niedermayr, all the kConFab research nurses and staff, the heads and staff of the Family Cancer Clinics, and the Clinical Follow Up Study (which has received funding from the NHMRC, the National Breast Cancer Foundation, Cancer Australia, and the National Institute of Health (USA)) for their contributions to this resource, and the many families who contribute to kConFab. We thank all investigators of the KOHBRA (Korean Hereditary Breast Cancer) study. LAABC thanks all the study participants and the entire data collection team, especially Annie Fung and June Yashiki. LMBC thanks Gilian Peuteman, Thomas Van Brussel, EvyVanderheyden and Kathleen Corthouts. MABCS thanks Milena Jakimovska (RCGEB “Georgi D. Efremov”), Snezhana Smichkoska, Emilija Lazarova (University Clinic of Radiotherapy and Oncology), Katerina Kubelka-Sabit, Mitko Karadjozov (Adzibadem-Sistina Hospital), Andrej Arsovski and Liljana Stojanovska (Re-Medika Hospital)

for their contributions and commitment to this study. MARIE thanks Petra Seibold, Dieter Flesch-Janys, Judith Heinz, Nadia Obi, Alina Vrieling, Sabine Behrens, Ursula Eilber, Muhabbet Celik, Til Olchers and Stefan Nickels. MBCSG (Milan Breast Cancer Study Group): Paolo Radice, Paolo Peterlongo, Siranoush Manoukian, Bernard Peissel, Jacopo Azzollini, Benedetta Beltrami, Daniela Zaffaroni, Bernardo Bonanni, Irene Feroce, Mariarosaria Calvello, Aliana Guerrieri Gonzaga, Monica Marabelli, Davide Bondavalli and the personnel of the Cogentech Cancer Genetic Test Laboratory. The MCCS was made possible by the contribution of many people, including the original investigators, the teams that recruited the participants and continue working on follow-up, and the many thousands of Melbourne residents who continue to participate in the study. We thank the coordinators, the research staff and especially the MMHS participants for their continued collaboration on research studies in breast cancer. MTLGEBCS would like to thank Martine Tranchant (CHU de Québec – Université Laval Research Center), Marie-France Valois, Annie Turgeon and Lea Heguy (McGill University Health Center, Royal Victoria Hospital; McGill University) for DNA extraction, sample management and skilful technical assistance. J.S. is Chair holder of the Canada Research Chair in Oncogenetics. MYBRCA thanks study participants and research staff (particularly Patsy Ng, Nurhidayu Hassan, Yoon Sook-Yee, Daphne Lee, Lee Sheau Yee, Phuah Sze Yee and Norhashimah Hassan) for their contributions and commitment to this study. The following are NBCS Collaborators: Anne-Lise Børresen-Dale (Prof. Em.), Kristine K. Sahlberg (PhD), Lars Ottestad (MD), Rolf Kåresen (Prof. Em.) Dr. Ellen Schlichting (MD), Marit Muri Holmen (MD), Toril Sauer (MD), Vilde Haakensen (MD), Olav Engebråten (MD), Bjørn Naume (MD), Alexander Fosså (MD), Cecile E. Kiserud (MD), Kristin V. Reinertsen (MD), Åslaug Helland (MD), Margit Riis (MD), Jürgen Geisler (MD), OSBREAC and Grethe I. Grenaker Alnæs (MSc). NBHS and SBCGS thank study participants and research staff for their contributions and commitment to the studies. For NHS and NHS2 the study protocol was approved by the institutional review boards of the Brigham and Women's Hospital and Harvard T.H. Chan School of Public Health, and those of participating registries as required. We would like to thank the participants and staff of the NHS and NHS2 for their valuable contributions as well as the following state cancer registries for their help: AL, AZ, AR, CA, CO, CT, DE, FL, GA, ID, IL, IN, IA, KY, LA, ME, MD, MA, MI, NE, NH, NJ, NY, NC, ND, OH, OK, OR, PA, RI, SC, TN, TX, VA, WA, WY. The authors assume full responsibility for analyses and interpretation of these data. OBCS thanks Arja Jukkola-Vuorinen, Mervi Grip, Saila Kauppila, Meeri Otsukka, Leena Keskitalo and Kari Mononen for their contributions to this study. The OFBCR thanks Teresa Selander, Nayana Weerasooriya and Steve Gallinger. ORIGO thanks E. Krol-Warmerdam, and J. Blom for patient accrual, administering questionnaires, and managing clinical information. The LUMC survival data were retrieved from the Leiden hospital-based cancer registry system (ONCDOC) with the help of Dr. J. Molenaar. PBCS thanks Louise Brinton, Mark Sherman, Neonila Szeszenia-Dabrowska, Beata Peplonska, Witold Zatonski, Pei Chao, Michael Stagner. The ethical approval for the POSH study is MREC /00/6/69, UKCRN ID: 1137. We thank staff in the Experimental Cancer Medicine Centre (ECMC) supported Faculty of Medicine Tissue Bank and the Faculty of Medicine DNA Banking resource. PREFACE thanks Sonja Oeser and Silke Landrith. SBCS thanks Sue Higham, Helen Cramp, Dan Connley, Ian Brock, Sabapathy Balasubramanian and Malcolm W.R. Reed. We thank the SEARCH and EPIC teams. SGBCC thanks the participants and all research coordinators for their excellent help with recruitment, data and sample collection. SKKDKFZS thanks all study participants, clinicians, family doctors, researchers and technicians for their contributions and commitment to this study. We thank the SUCCESS Study teams in Munich, Duessldorf, Erlangen and Ulm. SZBCS thanks Ewa Putresza. UCIBCS thanks Irene Masunaka. UKBGS thanks Breast Cancer Now and the Institute of Cancer Research for support and funding of the Generations Study, and the study participants, study staff, and the doctors, nurses and other health care providers and health information sources who have contributed to the study. We acknowledge NHS funding to the Royal Marsden/ICR NIHR Biomedical Research Centre. The authors

thank the WHI investigators and staff for their dedication and the study participants for making the program possible.

From ILLCO: We would like to thank all investigators who have contributed to the individual cancer GWAS. We thank the study PIs for the data contribution to lung cancer summary statistics.

From ECAC: We thank the many women who participated in the Endometrial Cancer Association Consortium, and the numerous institutions and their staff who supported recruitment.

From esophageal cancer GWAS consortium: The MD Anderson controls were drawn from dbGaP (study accession: phs000187.v1.p1). Genotyping of these controls were done through the University of Texas MD Anderson Cancer Center (UTMDACC) and the Johns Hopkins University Center for Inherited Disease Research (CIDR). We acknowledge the principal investigators of this study: Christopher Amos, Qingyi Wei, and Jeffrey E Lee. Controls from the Genome-Wide Association Study of Parkinson Disease were obtained from dbGaP (study accession: phs000196.v2.p1). This work, in part, used data from the National Institute of Neurological Disorders and Stroke (NINDS) dbGaP database from the CIDR:NeuroGenetics Research Consortium Parkinson's disease study. We acknowledge the principal investigators and coinvestigators of this study: Haydeh Payami, John Nutt, Cyrus Zabetian, Stewart Factor, Eric Molho, and Donald Higgins. Controls from the Chronic Renal Insufficiency Cohort (CRIC) were drawn from dbGaP (study accession: phs000524.v1.p1). The CRIC study was done by the CRIC investigators and supported by the National Institute of Diabetes and Digestive and Kidney Diseases (NIDDK). Data and samples from CRIC reported here were supplied by NIDDK Central Repositories. This report was not prepared in collaboration with investigators of the CRIC study and does not necessarily reflect the opinions or views of the CRIC study, the NIDDK Central Repositories, or the NIDDK. We acknowledge the principal investigators and the project officer of this study: Harold I Feldman, Raymond R Townsend, Lawrence J Appel, Mahboob Rahman, Akinlolu Ojo, James P Lash, Jiang He, Alan S Go, and John W Kusek.

From OCAC: We are grateful to the family and friends of Kathryn Sladek Smith for their generous support of the Ovarian Cancer Association Consortium through their donations to the Ovarian Cancer Research Fund. The OncoArray and COGS genotyping projects would not have been possible without the contributions of the following: Per Hall (COGS); Douglas F. Easton, Paul Pharoah, Kyriaki Michailidou, Manjeet K. Bolla, Qin Wang (BCAC), Andrew Berchuck, Marjorie J. Riggan (OCAC), Rosalind A. Eeles, Douglas F. Easton, Ali Amin Al Olama, Zsofia Kote-Jarai, Sara Benlloch (PRACTICAL), Georgia Chenevix-Trench, Antonis Antoniou, Lesley McGuffog, Fergus Couch and Ken Offit (CIMBA), Joe Dennis, Jonathan P. Tyrer, Siddhartha Kar, Alison M. Dunning, Andrew Lee, and Ed Dicks, Craig Luccarini and the staff of the Centre for Genetic Epidemiology Laboratory, Javier Benitez, Anna Gonzalez-Neira and the staff of the CNIO genotyping unit, Jacques Simard and Daniel C. Tessier, Francois Bacot, Daniel Vincent, Sylvie LaBoissière and Frederic Robidoux and the staff of the McGill University and Génome Québec Innovation Centre, Stig E. Bojesen, Sune F. Nielsen, Borge G. Nordestgaard, and the staff of the Copenhagen DNA laboratory, and Julie M. Cunningham, Sharon A. Windebank, Christopher A. Hilker, Jeffrey Meyer and the staff of Mayo Clinic Genotyping Core Facility. We pay special tribute to the contribution of Professor Brian Henderson to the GAME-ON consortium; to Olga M. Sinilnikova for her contribution to CIMBA and for her part in the initiation and coordination of GEMO until she sadly passed away on the 30th June 2014 and to Catherine M. Phelan for her contribution to OCAC and coordination of the OncoArray until she passed away on 22 September 2017. We thank the study participants, doctors, nurses, clinical and scientific collaborators, health care providers and health information sources who have contributed to the many studies contributing to this manuscript.

From CCFR, CORECT and GECCO: ASTERISK: We are very grateful to Dr. Bruno Buecher without whom this project would not have existed. We also thank all those who agreed to participate in this study, including the patients and the healthy control persons, as well as all the physicians, technicians and students. CCFR: The Colon CFR graciously thanks the generous contributions of their study participants, dedication of study staff, and the financial support from the U.S. National Cancer Institute, without which this important registry would not exist. CLUE: We appreciate the continued efforts of the staff members at the Johns Hopkins George W. Comstock Center for Public Health Research and Prevention in the conduct of the CLUE II study. We thank the participants in CLUE. Cancer incidence data for CLUE were provided by the Maryland Cancer Registry, Center for Cancer Surveillance and Control, Maryland Department of Health, 201 W. Preston Street, Room 400, Baltimore, MD 21201, http://phpa.dhmh.maryland.gov/cancer, 410-767-4055. We acknowledge the State of Maryland, the Maryland Cigarette Restitution Fund, and the National Program of Cancer Registries of the Centers for Disease Control and Prevention for the funds that support the collection and availability of the cancer registry data. COLON and NQplus: the authors would like to thank the COLON and NQplus investigators at Wageningen University & Research and the involved clinicians in the participating hospitals. CORSA: We kindly thank all individuals who agreed to participate in the CORSA study. Furthermore, we thank all cooperating physicians and students and the Biobank Graz of the Medical University of Graz. CPS-II: The authors thank the CPS-II participants and Study Management Group for their invaluable contributions to this research. The authors would also like to acknowledge the contribution to this study from central cancer registries supported through the Centers for Disease Control and Prevention National Program of Cancer Registries, and cancer registries supported by the National Cancer Institute Surveillance Epidemiology and End Results program. Czech Republic CCS: We are thankful to all clinicians in major hospitals in the Czech Republic, without whom the study would not be practicable. We are also sincerely grateful to all patients participating in this study. DACHS: We thank all participants and cooperating clinicians, and Ute Handte-Daub, Utz Benscheid, Muhabbet Celik and Ursula Eilber for excellent technical assistance. EDRN: We acknowledge all the following contributors to the development of the resource: University of Pittsburgh School of Medicine, Department of Gastroenterology, Hepatology and Nutrition: Lynda Dzubinski; University of Pittsburgh School of Medicine, Department of Pathology: Michelle Bisceglia; and University of Pittsburgh School of Medicine, Department of Biomedical Informatics. EPIC: Where authors are identified as personnel of the International Agency for Research on Cancer/World Health Organization, the authors alone are responsible for the views expressed in this article and they do not necessarily represent the decisions, policy or views of the International Agency for Research on Cancer/World Health Organization. EPICOLON: We are sincerely grateful to all patients participating in this study who were recruited as part of the EPICOLON project. We acknowledge the Spanish National DNA Bank, Biobank of Hospital Clínic–IDIBAPS and Biobanco Vasco for the availability of the samples. The work was carried out (in part) at the Esther Koplowitz Centre, Barcelona. Harvard cohorts (HPFS, NHS, PHS): The study protocol was approved by the institutional review boards of the Brigham and Women’s Hospital and Harvard T.H. Chan School of Public Health, and those of participating registries as required.We acknowledge Channing Division of Network Medicine, Department of Medicine, Brigham and Women's Hospital as home of the NHS.We would like to thank the participants and staff of the HPFS, NHS and PHS for their valuable contributions as well as the following state cancer registries for their help: AL, AZ, AR, CA, CO, CT, DE, FL, GA, ID, IL, IN, IA, KY, LA, ME, MD, MA, MI, NE, NH, NJ, NY, NC, ND, OH, OK, OR, PA, RI, SC, TN, TX, VA, WA, WY.The authors assume full responsibility for analyses and interpretation of these data. Kentucky: We would like to acknowledge the staff at the Kentucky Cancer Registry. LCCS: We acknowledge the contributions of Jennifer Barrett, Robin Waxman, Gillian Smith and Emma Northwood in conducting this study. NCCCS I & II: We would like to thank the study participants, and the NC Colorectal Cancer Study staff. NSHDS investigators thank the Västerbotten Intervention Programme, the Northern Sweden MONICA study, the

Biobank Research Unit at Umeå University and Biobanken Norr at Region Västerbotten for providing data and samples and acknowledge the contribution from Biobank Sweden, supported by the Swedish Research Council. PLCO: The authors thank the PLCO Cancer Screening Trial screening center investigators and the staff from Information Management Services Inc and Westat Inc. Most importantly, we thank the study participants for their contributions that made this study possible. Cancer incidence data have been provided by the District of Columbia Cancer Registry, Georgia Cancer Registry, Hawaii Cancer Registry, Minnesota Cancer Surveillance System, Missouri Cancer Registry, Nevada Central Cancer Registry, Pennsylvania Cancer Registry, Texas Cancer Registry, Virginia Cancer Registry, and Wisconsin Cancer Reporting System. All are supported in part by funds from the Center for Disease Control and Prevention, National Program for Central Registries, local states or by the National Cancer Institute, Surveillance, Epidemiology, and End Results program. The results reported here and the conclusions derived are the sole responsibility of the authors. SCCFR: The authors would like to thank the study participants and staff of the Seattle Colon Cancer Family Registry and the Hormones and Colon Cancer study (CORE Studies). SEARCH: We thank the SEARCH team. SELECT: We thank the research and clinical staff at the sites that participated on SELECT study, without whom the trial would not have been successful. We are also grateful to the 35,533 dedicated men who participated in SELECT. WHI: The authors thank the WHI investigators and staff for their dedication, and the study participants for making the program possible. A full listing of WHI investigators can be found at: http://www.whi.org/researchers/Documents%20%20Write%20a%20Paper/WHI%20Investigator%20Short%20List.pdfEsophageal cancer GWAS consortium: US National Cancer Institute, US National Institutes of Health, National Health and Medical Research Council of Australia, Swedish Cancer Society, Medical Research Council UK, Cambridge NIHR Biomedical Research Centre, Cambridge Experimental Cancer Medicine Centre, Else Kröner Fresenius Stiftung, Wellcome Trust, Cancer Research UK, AstraZeneca UK, University Hospitals of Leicester, University of Oxford, Australian Research Council.

<u>From OCAC individual studies:</u> **AUS:** The AOCS also acknowledges the cooperation of the participating institutions in Australia, and the contribution of the study nurses, research assistants and all clinical and scientific collaborators. The complete AOCS Study Group can be found at www.aocstudy.org. We would like to thank all of the women who participated in this research program; **BEL:** We would like to thank Gilian Peuteman, Thomas Van Brussel, Annick Van den Broeck and Joke De Roover for technical assistance; **BGS:** The Institute of Cancer Research (ICR) acknowledges NHS funding to the NIHR Biomedical Research Centre. We thank the Study staff, study participants , doctors, nurses, health care providers and health information sources who have contributed to the study and Breast Cancer Now and The Institute of Cancer Research for their support; **BVU:** The dataset(s) used for the analyses described were obtained from Vanderbilt University Medical Center’s BioVU; **CHA:** Innovative Research Team in University (PCSIRT) in China (IRT1076); **CHN:** To thank all members of Department of Obstetrics and Gynaecology, Hebei Medical University, Fourth Hospital and Department of Molecular Biology, Hebei Medical University, Fourth Hospital; **EPC:** To thank all members and investigators of the Rotterdam Ovarian Cancer Study; **GER:** The German Ovarian Cancer Study (GER) thank Ursula Eilber for competent technical assistance; **MAS:** We would like to thank Famida Zulkifli and Ms Moey for assistance in patient recruitment, data collection and sample preparation; **MCC:** Cases and their vital status were ascertained through the Victorian Cancer Registry (VCR) and the Australian Institute of Health and Welfare (AIHW), including the National Death Index and the Australian Cancer Database; **MOF:** the Total Cancer Care™ Protocol and the Collaborative Data Services and Tissue Core Facilities at the H. Lee Moffitt Cancer Center & Research Institute, an NCI designated Comprehensive Cancer Center (P30-CA076292), Merck Pharmaceuticals and the state of Florida; **NHS:** The NHS/NHSII studies thank the following state cancer registries for their help: AL, AZ, AR, CA, CO, CT, DE, FL, GA, ID, IL, IN, IA, KY, LA, ME, MD, MA,

MI, NE, NH, NJ, NY, NC, ND, OH, OK, OR, PA, RI, SC, TN, TX, VA, WA, and WY; **OPL:** Members of the OPAL Study Group (http://opalstudy.qimrberghofer.edu.au/); **SEA:** SEARCH team, Craig Luccarini, Caroline Baynes, Don Conroy; **SRO:** To thank all members of Scottish Gynaecological Clinical Trails group and SCOTROC1 investigators; **SWH:** We thank the participants and the research staff of the Shanghai Women's Health Study for making this study possible; **UHN:** Princess Margaret Cancer Centre Foundation-Bridge for the Cure; **UKO:** We particularly thank I. Jacobs, M.Widschwendter, E. Wozniak, A. Ryan, J. Ford and N. Balogun for their contribution to the study**; UKR:** Carole Pye; **VAN:** BC Cancer Foundation, VGH & UBC Hospital Foundation; **WMH:** We thank the Gynaecological Oncology Biobank at Westmead, a member of the Australasian Biospecimen Network-Oncology group.

Consortium Member List

Breast Cancer Association Consortium (BCAC) Members

Thomas U. Ahearn[1], Irene L. Andrulis[2, 3], Hoda Anton-Culver[4], Kristan J. Aronson[5], Matthias W. Beckmann[6], Javier Benitez[7, 8], Marina Bermisheva[9], Natalia V. Bogdanova[10-12], Stig E. Bojesen[13-15], Manjeet K. Bolla[16], Hiltrud Brauch[17-19], Hermann Brenner[20-22], Sara Y. Brucker[23], Barbara Burwinkel[24, 25], Saundra S. Buys[26], Federico Canzian[27], Jenny Chang-Claude[28, 29], Georgia Chenevix-Trench[30], Christine L. Clarke[31], Fergus J. Couch[32], Angela Cox[33], Kamila Czene[34], Mary B. Daly[35], Peter Devilee[36, 37], Thilo Dörk[11], Alison M. Dunning[38], Miriam Dwek[39], Diana M. Eccles[40], Peter A. Fasching[6, 41], Olivia Fletcher[42], Lin Fritschi[43], Manuela Gago-Dominguez[44, 45], Montserrat García-Closas[1], José A. García-Sáenz[46], Mark S. Goldberg[47, 48], Pascal Guénel[49], Per Hall[34, 50], Ute Hamann[51], Mikael Hartman[52, 53], Alexander Hein[6], Antoinette Hollestelle[54], Maartje J. Hooning[54], John L. Hopper[55], David J. Hunter[56, 57], ABCTB Investigators[58], Motoki Iwasaki[59], Anna Jakubowska[60, 61], Esther M. John[62, 63], Rudolf Kaaks[28], Daehee Kang[64, 65], Vessela N. Kristensen[66, 67], Ava Kwong[68-70], James V. Lacey[71, 72], Diether Lambrechts[73, 74], Jingmei Li[75], Annika Lindblom[76, 77], Arto Mannermaa[78-80], Keitaro Matsuo[81, 82], Roger L. Milne[55, 83, 84], Kenneth Muir[85], Rachel A. Murphy[86, 87], Heli Nevanlinna[88], Håkan Olsson[89], Sue K. Park[64, 65, 90], Dijana Plaseska-Karanfilska[91], Ross Prentice[92], Paolo Radice[93], Gad Rennert[94], Emmanouil Saloustros[95], Dale P. Sandler[96], Elinor J. Sawyer[97], Marjanka K. Schmidt[98, 99], Rita K. Schmutzler[100-102], Lukas Schwentner[103], Chen-Yang Shen[104, 105], Xiao-Ou Shu[106], Melissa C. Southey[83, 84, 107], Anthony J. Swerdlow[108, 109], Rulla M. Tamimi[57, 110], Jack A. Taylor[96, 111], Soo Hwang Teo[112, 113], Lauren R. Teras[114], Mary Beth Terry[115], Celine M. Vachon[116], Qin Wang[16], Clarice R. Weinberg[117], Robert Winqvist[118, 119], Alicja Wolk[120, 121], Anna H. Wu[122], Wei Zheng[106]

Affiliations: [1] Division of Cancer Epidemiology and Genetics, National Cancer Institute, National Institutes of Health, Department of Health and Human Services, Bethesda, MD, USA; [2] Fred A. Litwin Center for Cancer Genetics, Lunenfeld-Tanenbaum Research Institute of Mount Sinai Hospital, Toronto, ON, Canada; [3] Department of Molecular Genetics, University of Toronto, Toronto, ON, Canada; [4] Department of Medicine, Genetic Epidemiology Research Institute, University of California Irvine, Irvine, CA, USA; [5] Department of Public Health Sciences, and Cancer Research Institute, Queen's University, Kingston, ON, Canada; [6] Department of Gynecology and Obstetrics, Comprehensive Cancer Center Erlangen-EMN, University Hospital Erlangen, Friedrich-Alexander University Erlangen-Nuremberg (FAU), Erlangen, Germany; [7] Biomedical Network on Rare Diseases (CIBERER), Madrid, Spain; [8] Human Cancer Genetics Programme, Spanish National Cancer Research Centre (CNIO), Madrid, Spain; [9] Institute of Biochemistry and Genetics, Ufa Federal Research Centre of the Russian Academy of Sciences, Ufa, Russia; [10] Department of Radiation Oncology, Hannover Medical School, Hannover, Germany; [11] Gynaecology Research Unit, Hannover Medical School, Hannover, Germany; [12] N.N. Alexandrov Research Institute of Oncology and Medical Radiology, Minsk, Belarus; [13] Copenhagen General Population Study, Herlev and Gentofte Hospital, Copenhagen University Hospital, Herlev, Denmark; [14] Department of Clinical Biochemistry, Herlev and Gentofte Hospital, Copenhagen University Hospital, Herlev, Denmark; [15] Faculty of Health and Medical Sciences, University of Copenhagen, Copenhagen, Denmark; [16] Centre for Cancer Genetic Epidemiology, Department of Public Health and Primary Care, University of Cambridge, Cambridge, UK; [17] Dr. Margarete Fischer-Bosch-Institute of Clinical Pharmacology, Stuttgart, Germany; [18] iFIT-Cluster of Excellence, University of Tübingen, Tübingen, Germany; [19] German Cancer Consortium (DKTK) and German Cancer Research Center (DKFZ), Partner Site Tübingen, Tübingen, Germany; [20] Division of Clinical Epidemiology and Aging Research, German Cancer Research Center (DKFZ), Heidelberg, Germany; [21] Division of Preventive Oncology, German Cancer Research Center (DKFZ) and National Center for Tumor Diseases (NCT), Heidelberg, Germany; [22] German Cancer Consortium (DKTK),

German Cancer Research Center (DKFZ), Heidelberg, Germany; [23] Department of Gynecology and Obstetrics, University of Tübingen, Tübingen, Germany; [24] Molecular Epidemiology Group, C080, German Cancer Research Center (DKFZ), Heidelberg, Germany; [25] Molecular Biology of Breast Cancer, University Womens Clinic Heidelberg, University of Heidelberg, Heidelberg, Germany; [26] Department of Medicine, Huntsman Cancer Institute, Salt Lake City, UT, USA; [27] Genomic Epidemiology Group, German Cancer Research Center (DKFZ), Heidelberg, Germany; [28] Division of Cancer Epidemiology, German Cancer Research Center (DKFZ), Heidelberg, Germany; [29] Cancer Epidemiology Group, University Cancer Center Hamburg (UCCH), University Medical Center Hamburg-Eppendorf, Hamburg, Germany; [30] Department of Genetics and Computational Biology, QIMR Berghofer Medical Research Institute, Brisbane, Queensland, Australia; [31] Westmead Institute for Medical Research, University of Sydney, Sydney, New South Wales, Australia; [32] Department of Laboratory Medicine and Pathology, Mayo Clinic, Rochester, MN, USA; [33] Sheffield Institute for Nucleic Acids (SInFoNiA), Department of Oncology and Metabolism, University of Sheffield, Sheffield, UK; [34] Department of Medical Epidemiology and Biostatistics, Karolinska Institutet, Stockholm, Sweden; [35] Department of Clinical Genetics, Fox Chase Cancer Center, Philadelphia, PA, USA; [36] Department of Pathology, Leiden University Medical Center, Leiden, The Netherlands; [37] Department of Human Genetics, Leiden University Medical Center, Leiden, The Netherlands; [38] Centre for Cancer Genetic Epidemiology, Department of Oncology, University of Cambridge, Cambridge, UK; [39] School of Life Sciences, University of Westminster, London, UK; [40] Faculty of Medicine, University of Southampton, Southampton, UK; [41] David Geffen School of Medicine, Department of Medicine Division of Hematology and Oncology, University of California at Los Angeles, Los Angeles, CA, USA; [42] The Breast Cancer Now Toby Robins Research Centre, The Institute of Cancer Research, London, UK; [43] School of Public Health, Curtin University, Perth, Western Australia, Australia; [44] Fundación Pública Galega de Medicina Xenómica, Instituto de Investigación Sanitaria de Santiago de Compostela (IDIS), Complejo Hospitalario Universitario de Santiago, SERGAS, Santiago de Compostela, Spain; [45] Moores Cancer Center, University of California San Diego, La Jolla, CA, USA; [46] Medical Oncology Department, Hospital Clínico San Carlos, Instituto de Investigación Sanitaria San Carlos (IdISSC), Centro Investigación Biomédica en Red de Cáncer (CIBERONC), Madrid, Spain; [47] Department of Medicine, McGill University, Montréal, QC, Canada; [48] Division of Clinical Epidemiology, Royal Victoria Hospital, McGill University, Montréal, QC, Canada; [49] Center for Research in Epidemiology and Population Health (CESP), Team Exposome and Heredity, INSERM, University Paris-Saclay, Villejuif, France; [50] Department of Oncology, Södersjukhuset, Stockholm, Sweden; [51] Molecular Genetics of Breast Cancer, German Cancer Research Center (DKFZ), Heidelberg, Germany; [52] Saw Swee Hock School of Public Health, National University of Singapore and National University Health System, Singapore, Singapore; [53] Department of Surgery, National University Health System, Singapore, Singapore; [54] Department of Medical Oncology, Erasmus MC Cancer Institute, Rotterdam, The Netherlands; [55] Centre for Epidemiology and Biostatistics, Melbourne School of Population and Global Health, The University of Melbourne, Melbourne, Victoria, Australia; [56] Nuffield Department of Population Health, University of Oxford, Oxford, UK; [57] Department of Epidemiology, Harvard T.H. Chan School of Public Health, Boston, MA, USA; [58] Australian Breast Cancer Tissue Bank, Westmead Institute for Medical Research, University of Sydney, Sydney, New South Wales, Australia; [59] Division of Epidemiology, Center for Public Health Sciences, National Cancer Center, Tokyo, Japan; [60] Department of Genetics and Pathology, Pomeranian Medical University, Szczecin, Poland; [61] Independent Laboratory of Molecular Biology and Genetic Diagnostics, Pomeranian Medical University, Szczecin, Poland; [62] Department of Epidemiology & Population Health, Stanford University School of Medicine, Stanford, CA, USA; [63] Department of Medicine, Division of Oncology, Stanford Cancer Institute, Stanford University School of Medicine, Stanford, CA, USA; [64] Department of Preventive Medicine, Seoul National University College of Medicine, Seoul, Korea; [65] Cancer Research Institute, Seoul National University, Seoul, Korea; [66] Department of Medical Genetics, Oslo University Hospital and University of Oslo, Oslo, Norway; [67]

Institute of Clinical Medicine, Faculty of Medicine, University of Oslo, Oslo, Norway; [68] Hong Kong Hereditary Breast Cancer Family Registry, Hong Kong; [69] Department of Surgery, The University of Hong Kong, Hong Kong; [70] Department of Surgery and Cancer Genetics Center, Hong Kong Sanatorium and Hospital, Hong Kong; [71] Department of Computational and Quantitative Medicine, City of Hope, Duarte, CA, USA; [72] City of Hope Comprehensive Cancer Center, City of Hope, Duarte, CA, USA; [73] VIB Center for Cancer Biology, Leuven, Belgium; [74] Laboratory for Translational Genetics, Department of Human Genetics, University of Leuven, Leuven, Belgium; [75] Human Genetics Division, Genome Institute of Singapore, Singapore, Singapore; [76] Department of Molecular Medicine and Surgery, Karolinska Institutet, Stockholm, Sweden; [77] Department of Clinical Genetics, Karolinska University Hospital, Stockholm, Sweden; [78] Translational Cancer Research Area, University of Eastern Finland, Kuopio, Finland; [79] Institute of Clinical Medicine, Pathology and Forensic Medicine, University of Eastern Finland, Kuopio, Finland; [80] Biobank of Eastern Finland, Kuopio University Hospital, Kuopio, Finland; [81] Division of Cancer Epidemiology and Prevention, Aichi Cancer Center Research Institute, Nagoya, Japan; [82] Division of Cancer Epidemiology, Nagoya University Graduate School of Medicine, Nagoya, Japan; [83] Cancer Epidemiology Division, Cancer Council Victoria, Melbourne, Victoria, Australia; [84] Precision Medicine, School of Clinical Sciences at Monash Health, Monash University, Clayton, Victoria, Australia; [85] Division of Population Health, Health Services Research and Primary Care, School of Health Sciences, Faculty of Biology, Medicine and Health, The University of Manchester, Manchester, UK; [86] School of Population and Public Health, University of British Columbia, Vancouver, BC, Canada; [87] Cancer Control Research, BC Cancer, Vancouver, BC, Canada; [88] Department of Obstetrics and Gynecology, Helsinki University Hospital, University of Helsinki, Helsinki, Finland; [89] Department of Cancer Epidemiology, Clinical Sciences, Lund University, Lund, Sweden; [90] Integrated Major in Innovative Medical Science, Seoul National University College of Medicine, Seoul, South Korea; [91] Research Centre for Genetic Engineering and Biotechnology 'Georgi D. Efremov', MASA, Skopje, Republic of North Macedonia; [92] Cancer Prevention Program, Fred Hutchinson Cancer Research Center, Seattle, WA, USA; [93] Unit of Molecular Bases of Genetic Risk and Genetic Testing, Department of Research, Fondazione IRCCS Istituto Nazionale dei Tumori (INT), Milan, Italy; [94] Clalit National Cancer Control Center, Carmel Medical Center and Technion Faculty of Medicine, Haifa, Israel; [95] Department of Oncology, University Hospital of Larissa, Larissa, Greece; [96] Epidemiology Branch, National Institute of Environmental Health Sciences, NIH, Research Triangle Park, NC, USA; [97] School of Cancer & Pharmaceutical Sciences, Comprehensive Cancer Centre, Guy’s Campus, King's College London, London, UK; [98] Division of Molecular Pathology, The Netherlands Cancer Institute - Antoni van Leeuwenhoek Hospital, Amsterdam, The Netherlands; [99] Division of Psychosocial Research and Epidemiology, The Netherlands Cancer Institute - Antoni van Leeuwenhoek hospital, Amsterdam, The Netherlands; [100] Center for Familial Breast and Ovarian Cancer, Faculty of Medicine and University Hospital Cologne, University of Cologne, Cologne, Germany; [101] Center for Integrated Oncology (CIO), Faculty of Medicine and University Hospital Cologne, University of Cologne, Cologne, Germany; [102] Center for Molecular Medicine Cologne (CMMC), Faculty of Medicine and University Hospital Cologne, University of Cologne, Cologne, Germany; [103] Department of Gynaecology and Obstetrics, University Hospital Ulm, Ulm, Germany; [104] Institute of Biomedical Sciences, Academia Sinica, Taipei, Taiwan; [105] School of Public Health, China Medical University, Taichung, Taiwan; [106] Division of Epidemiology, Department of Medicine, Vanderbilt Epidemiology Center, Vanderbilt-Ingram Cancer Center, Vanderbilt University School of Medicine, Nashville, TN, USA; [107] Department of Clinical Pathology, The University of Melbourne, Melbourne, Victoria, Australia; [108] Division of Genetics and Epidemiology, The Institute of Cancer Research, London, UK; [109] Division of Breast Cancer Research, The Institute of Cancer Research, London, UK; [110] Department of Population Health Sciences, Weill Cornell Medicine, New York, NY, USA; [111] Epigenetic and Stem Cell Biology Laboratory, National Institute of Environmental Health Sciences, NIH, Research Triangle Park, NC, USA; [112] Breast Cancer

Research Programme, Cancer Research Malaysia, Subang Jaya, Selangor, Malaysia; [113] Department of Surgery, Faculty of Medicine, University of Malaya, Kuala Lumpur, Malaysia; [114] Department of Population Science, American Cancer Society, Atlanta, GA, USA; [115] Department of Epidemiology, Mailman School of Public Health, Columbia University, New York, NY, USA; [116] Department of Health Science Research, Division of Epidemiology, Mayo Clinic, Rochester, MN, USA; [117] Biostatistics and Computational Biology Branch, National Institute of Environmental Health Sciences, NIH, Research Triangle Park, NC, USA; [118] Laboratory of Cancer Genetics and Tumor Biology, Cancer and Translational Medicine Research Unit, Biocenter Oulu, University of Oulu, Oulu, Finland; [119] Laboratory of Cancer Genetics and Tumor Biology, Northern Finland Laboratory Centre Oulu, Oulu, Finland; [120] Institute of Environmental Medicine, Karolinska Institutet, Stockholm, Sweden; [121] Department of Surgical Sciences, Uppsala University, Uppsala, Sweden; [122] Department of Preventive Medicine, Keck School of Medicine, University of Southern California, Los Angeles, CA, USA

Colon Cancer Family Registry (CCFR), Colorectal Cancer Transdisciplinary (CORECT) and Genetics and Epidemiology of Colorectal Cancer Consortium (GECCO) Members

Goncalo R Abecasis[1], Demetrius Albanes[2], M Henar Alonso[3,4,5], Kristin Anderson[6], Coral Arnau-Collell[7], Volker Arndt[8], Christina Bamia[9,10], Elizabeth L Barry[11], Michael C Bassik[12], Sonja I Berndt[13], Stéphane Bézieau[14], Stephanie Bien[15], D Timothy Bishop[16], Juergen Boehm[17], Heiner Boeing[18], Hermann Brenner[8,20,21], Stefanie Brezina[21], Stephan Buch[22], Daniel D Buchanan[23,24,25], Andrea Burnett-Hartman[26], Bette J Caan[27], Qiuyin Cai[28], Peter T Campbell[29], Christopher S Carlson[2,30], Graham Casey[31], Jose Esteban Castelao[32], Sergi Castellví-Bel[33], Andrew T Chan[34,35,36,37], Jenny Chang-Claude[38,39], Stephen J Chanock[2], Sai Chen[1], Lee Soon[40], Maria-Dolores Chirlaque[4, 41], Sang Hee Cho[42], James Church[43], Gerhard Coetzee[44], David V Conti[45], Chiara Cremolini[46], Amanda J Cross[47,48], Marcia Cruz-Correa[49], Katarina Cuk[8], Keith R Curtis[15], Albert de la Chapelle[50], Kimberly F Doheny[51], David Duggan[52], Douglas F Easton[53], Christopher K Edlund[45], Sjoerd G Elias[54], Faye Elliott[16], Dallas R English[55,56], Alfredo Falcone[46], Jane C Figueiredo[45,57], Liesel M FitzGerald[56,58], Charles Fuchs[35,59], Manuela Gago-Dominguez[60], Manish Gala[34,36], Steven J Gallinger[61,62], William Gauderman[45], Graham G Giles[55,56], Edward Giovannucci[63,64], Jian Gong[15], Phyllis J Goodman[65], William M Grady[66], Peyton Greenside[67], Joel Greenson[68], John S Grove[69], Stephen B Gruber[70], Andrea Gsur[21], Marc J Gunter[71], Robert W Haile[72], Christopher A, Haiman[45], Jochen Hampe[22], Heather Hampel[73], Sophia Harlid[74], Tabitha A Harrison[15], Richard B Hayes[75], Volker Heinemann[76], Philipp Hofer[21], Michael Hoffmeister[8], John L Hopper[55], Wan-Ling Hsu[64], Li Hsu[15,64], Wen-Yi Huang[2], Thomas J Hudson[77], David J Hunter[78,79], Jeroen R Huyghe[15], Gregory E Idos[70], Rebecca Jackson[73], Mark A Jenkins[55], Jihyoun Jeon[80], Amit D Joshi[36,78], Corinne E Joshu[81], Hyun Min Kang[1], Temitope O Keku[82], Timothy J Key[79], Hyeong Rok Kim[42], Laurence N Kolonel[83], Charles Kooperberg[15], Tilman Kuhn[38], Anshul Kundaje[12,84], Sébastien Küry[14], Sun-Seog Kweon[42], Susanna C Larsson[85], Cecelia A Laurie[64], Loic Le Marchand[69], Suzanne M Leal[86], Soo Chin Lee[40], Flavio Lejbkowicz[87,88,89], Heinz-Josef Lenz[45], David M Levine[64], Christopher I Li[15], Li Li[90], Wolfgang Lieb[91], Yi Lin[15], Annika Lindblom[92,93], Noralane M Lindor[94], Hua Ling[51], Yun-Ru Liu[95], Tin L Louie[64], Fotios Loupakis[96], Frank Luh[97], Satu Männistö[98], Sanford D Markowitz[90], Vicente Martín[4,99], Giovanna Masala[100], Kevin J McDonnell[70], Caroline E McNeil[45], Marilena Melas[101], Roger L Milne[55,56], Lorena Moreno[7], Victor Moreno[4], Bhramar Mukherjee[102], Victor Muñoz-Garzón[32], Neil Murphy[71], Alessio Naccarati[103,104], Sarah C Nelson[64], Polly A Newcomb[15,30] , Deborah A Nickerson[105], Kenneth Offit[106,107], Shuji Ogino[35,37,59,78], N Charlotte Onland-Moret[54], Barbara Pardini[103,104], Patrick S Parfrey[108], Rachel Pearlman[50], Vittorio Perduca[109,110], Julyann Pérez-Mayoral[49], Ulrike Peters[15,30], Paul D P Pharoah[53], Mila Pinchev[88], Elizabeth A Platz[81], Sarah Plummer[31], John D Potter[15,111], Ross L Prentice[15,64], Elizabeth Pugh[51], Conghui Qu[15], Chenxu Qu[45], Leon Raskin[28], Gad Rennert[88,89], Hedy S Rennert[88,89], Elio Riboli[47], Miguel Rodríguez-Barranco[4], Jane Romm[51], Lori C Sakoda[15,27], Peter C Scacheri[112], Clemens Schafmayer[113], Stephanie L Schmit[114], Robert E Schoen[115], Fredrick R Schumacher[116], Daniela Seminara[117], Gianluca Severi[56], Mitul Shah[118], Tameka Shelford[51], David Shibata[119], Min-Ho Shin[120], Xiao-Ou Shu[28], Katerina Shulman[121], Erin Siegel[122], Sabina Sieri[123], Nasa A Sinnott-Armstrong[12], Martha L Slattery[124], Joshua D Smith[105], Melissa C Southey[125], Zsofia K Stadler[106,107], Mariana Stern[45], Sebastian Stintzing[126], Yu-Ru Su[127], Catherine M Tangen[65], Stephen N Thibodeau[94], Sushma S Thomas[15], Amanda E Toland[73], Antonia Trichopoulou[9,10], Cornelia M Ulrich[17], David J Van Den Berg[45], Franzel JB van Duijnhoven[128], Bethany Van Guelpen[74], Henk van Kranen[129], Joseph Vijai[106], Kala Visvanathan[81], Pavel Vodicka[130,131,132], Ludmila Vodickova[130,131,132], Veronika Vymetalkova[130,131,132], Michael Wainberg[84], Hansong Wang[69], Korbinian Weigl[8,20,133], Stephanie J Weinstein[2], Emily White[15,30], Lynne R, Wilkens[69], Aung Ko Win[25,55], C Roland Wolf[134], Alicja Wolk[85], Michael O Woods[135], Anna H Wu[45], Yun Yen[95], Syed H Zaidi[77], Brent W Zanke[136], Wei Zheng[28]

Affiliations: [1]Department of Biostatistics and Center for Statistical Genetics, University of Michigan, Ann Arbor, Michigan, USA, [2]Division of Cancer Epidemiology and Genetics, National Cancer Institute,

National Institutes of Health, Bethesda, Maryland, USA, [3]Cancer Prevention and Control Program, Catalan Institute of Oncology-IDIBELL, L'Hospitalet de Llobregat, Barcelona, Spain, [4]CIBER Epidemiología y Salud Pública (CIBERESP), Madrid, Spain, [5]Department of Clinical Sciences, Faculty of Medicine, University of Barcelona, Barcelona, Spain, [6]Division of Epidemiology and Community Health, University of Minnesota, Minneapolis, Minnesota, USA, [7]Gastroenterology Department, Hospital Clínic, Institut d'Investigacions Biomèdiques August Pi i Sunyer (IDIBAPS), Centro de Investigación Biomédica en Red de Enfermedades Hepáticas y Digestivas (CIBEREHD), University of Barcelona, Barcelona, Spain, [8]Division of Clinical Epidemiology and Aging Research, German Cancer Research Center (DKFZ), Heidelberg, Germany, [9]Hellenic Health Foundation, Athens, Greece, [10]WHO Collaborating Center for Nutrition and Health, Unit of Nutritional Epidemiology and Nutrition in Public Health, Department of Hygiene, Epidemiology and Medical Statistics, School of Medicine, National and Kapodistrian University of Athens, Greece, [11]Department of Epidemiology, Geisel School of Medicine, Dartmouth College, Hanover, New Hampshire, USA, [12]Department of Genetics, Stanford University, Stanford, California, USA, [13]Division of Cancer Epidemiology and Genetics, National Cancer Institute, National Institutes of Health, Bethesda, Maryland, USA, [14]Service de Génétique Médicale, Centre Hospitalier Universitaire (CHU) Nantes, Nantes, France, [15]Public Health Sciences Division, Fred Hutchinson Cancer Research Center, Seattle, Washington, USA, [16]Leeds Institute of Cancer and Pathology, University of Leeds, Leeds, UK, [17]Huntsman Cancer Institute and Department of Population Health Sciences, University of Utah, Salt Lake City, Utah, USA, [18]Department of Epidemiology, German Institute of Human Nutrition (DIfE), Potsdam-Rehbrücke, Germany, [19]Division of Preventive Oncology, German Cancer Research Center (DKFZ) and National Center for Tumor Diseases (NCT), Heidelberg, Germany, [20]German Cancer Consortium (DKTK), German Cancer Research Center (DKFZ), Heidelberg, Germany, [21]Institute of Cancer Research, Department of Medicine I, Medical University Vienna, Vienna, Austria, [22]Department of Medicine I, University Hospital Dresden, Technische Universität Dresden (TU Dresden), Dresden, Germany, [23]Colorectal Oncogenomics Group, Department of Clinical Pathology, The University of Melbourne, Parkville, Victoria 3010 Australia, [24]University of Melbourne Centre for Cancer Research, Victorian Comprehensive Cancer Centre, Parkville, Victoria 3010 Australia, [25]Genomic Medicine and Family Cancer Clinic, The Royal Melbourne Hospital, Parkville, Victoria, Australia, [26]Institute for Health Research, Kaiser Permanente Colorado, Denver, Colorado, USA, [27]Division of Research, Kaiser Permanente Medical Care Program, Oakland, California, USA, [28]Division of Epidemiology, Department of Medicine, Vanderbilt Epidemiology Center, Vanderbilt-Ingram Cancer Center, Vanderbilt University Medical Center, Nashville, TN, USA, [29]Department of population Science, American Cancer Society, Atlanta, Georgia, USA, [30]Department of Epidemiology, University of Washington, Seattle, Washington, USA, [31]Center for Public Health Genomics, University of Virginia, Charlottesville, Virginia, USA, [32]Genetic Oncology Unit, Complexo Hospitalario Universitario de Vigo (CHUVI), SERGAS, Vigo, Spain, [33]Gastroenterology Department, Hospital Clínic, Institut d'Investigacions Biomèdiques August Pi i Sunyer (IDIBAPS), Centro de Investigación Biomédica en Red de Enfermedades Hepáticas y Digestivas (CIBEREHD), University of Barcelona, Barcelona, Spain, [34]Division of Gastroenterology, Massachusetts General Hospital and Harvard Medical School, Boston, Massachusetts, USA, [35]Channing Division of Network Medicine, Brigham and Women's Hospital and Harvard Medical School, Boston, Massachusetts, USA, [36]Clinical and Translational Epidemiology Unit, Massachusetts General Hospital and Harvard Medical School, Boston, Massachusetts, USA, [37]Broad Institute of Harvard and MIT, Cambridge, Massachusetts, USA, [38]Division of Cancer Epidemiology, German Cancer Research Center (DKFZ), Heidelberg, Germany, [39]University Medical Centre Hamburg-Eppendorf, University Cancer Centre Hamburg (UCCH), Hamburg, Germany, [40]National University Cancer Institute, Singapore; Cancer Science Institute of Singapore, National University of Singapore, Singapore, [41]Department of Epidemiology, Regional Health Council, IMIB-Arrixaca, Murcia University, Murcia, Spain, [42]Department of Hematology-Oncology, Chonnam National University Hospital,

Hwasun, South Korea, [43]Department of Colorectal Surgery, Cleveland Clinic, Cleveland, Ohio 44195, USA, [44]Van Andel Research Institute, Grand Rapids, Michigan 49502, USA, [45]Department of Preventive Medicine, USC Norris Comprehensive Cancer Center, Keck School of Medicine, University of Southern California, Los Angeles, California, USA, [46]University of Pisa, Pisa, Italy, [47]Department of Epidemiology and Biostatistics, Imperial College London, London, UK, [48]Department of Surgery and Cancer, Imperial College London, London, UK, [49]Comprehensive Cancer Center, University of Puerto Rico, San Juan, Puerto Rico, [50]Department of Cancer Biology and Genetics and the Comprehensive Cancer Center, The Ohio State University, Columbus, Ohio, USA, [51]Center for Inherited Disease Research (CIDR), Department of Genetic Medicine, Johns Hopkins University School of Medicine, Baltimore, Maryland, USA, [52]Translational Genomics Research Institute - An Affiliate of City of Hope, Phoenix, Arizona, USA, [53]Department of Public Health and Primary Care, University of Cambridge, Cambridge, UK, [54]Julius Center for Health Sciences and Primary Care, University Medical Center Utrecht, Utrecht, The Netherlands, [55]Centre for Epidemiology and Biostatistics, Melbourne School of Population and Global Health, The University of Melbourne, Melbourne, Victoria, Australia, [56]Cancer Epidemiology and Intelligence Division, Cancer Council Victoria, Melbourne, Victoria, Australia, [57]Department of Medicine, Samuel Oschin Comprehensive Cancer Institute, Cedars-Sinai Medical Center, Los Angeles, CA, USA, [58]Menzies Institute for Medical Research, University of Tasmania, Hobart, Tasmania, Australia, [59]Department of Medical Oncology, Dana-Farber Cancer Institute, Brookline, Massachusetts 02115, USA, [60]Genomic Medicine Group, Galician Foundation of Genomic Medicine, Complejo Hospitalario Universitario de Santiago, Servicio Galego de Saude (SERGAS), Instituto de Investigación Sanitaria de Santiago de Compostela (IDIS), Santiago De Compostela, Spain, [61]Lunenfeld Tanenbaum Research Institute, Mount Sinai Hospital, University of Toronto, Toronto, Ontario, Canada, [62]Zane Cohen Centre for Digestive Diseases, Mount Sinai Hospital, Toronto, Ontario M5T 3L9, Canada, [63]Harvard Medical School, Boston, Massachusetts 02114, USA, [64]Department of Biostatistics, University of Washington, Seattle, Washington 98195, USA, [65]SWOG Statistical Center, Fred Hutchinson Cancer Research Center, Seattle, Washington, USA, [66]Clinical Research Division, Fred Hutchinson Cancer Research Center, Seattle, Washington, USA, [67]Department of Biomedical Data Science, Stanford University, Stanford, California, USA, [68]Department of Pathology, University of Michigan, Ann Arbor, Michigan 48104, USA, [69]University of Hawaii Cancer Research Center, Honolulu, Hawaii, USA, [70]City of Hope National Medical Center, Duarte, California, USA, [71]Nutrition and Metabolism Section, International Agency for Research on Cancer, World Health Organization, Lyon, France, [72]Division of Oncology, Department of Medicine, Stanford University, Stanford, California, USA, [73]Division of Human Genetics, Department of Internal Medicine, The Ohio State University Comprehensive Cancer Center, Columbus, Ohio, USA, [74]Department of Radiation Sciences, Oncology Unit, Umeå University, Umeå, Sweden, [75]Division of Epidemiology, Department of Population Health, New York University School of Medicine, New York, New York, USA, [76]Department of Medical Oncology and Comprehensive Cancer Center, Ludwig Maximilians University - Grosshadern, Munich, Germany, [77]Ontario Institute for Cancer Research, Toronto, Ontario, Canada, [78]Department of Epidemiology, Harvard T,H, Chan School of Public Health, Harvard University, Boston, Massachusetts, USA, [79]Nuffield Department of Population Health, University of Oxford, Oxford, UK, [80]Department of Epidemiology, University of Michigan, Ann Arbor, Michigan, USA, [81]Department of Epidemiology, Johns Hopkins Bloomberg School of Public Health, Baltimore, Maryland, USA, [82]Center for Gastrointestinal Biology and Disease, University of North Carolina, Chapel Hill, North Carolina, USA, [83]Office of Public Health Studies, University of Hawaii Manoa, Honolulu, Hawaii, USA, [84]Department of Computer Science, Stanford University, Stanford, California, USA, [85]Institute of Environmental Medicine, Karolinska Institutet, Stockholm, Sweden, [86]Center for Statistical Genetics, Sergievsky Center, Taub Institute for Alzheimer's Disease and the Aging Brain, and the Department of Neurology, Columbia University Medical Center, New York, NY, [87]The Clalit Health Services, Personalized Genomic Service, Carmel, Haifa, Israel, [88]Department

of Community Medicine and Epidemiology, Carmel Medical Center, Haifa, Israel, [89]Clalit National Cancer Control Center, Haifa, Israel, [90]Case Comprehensive Cancer Center, Case Western Reserve University, Cleveland, Ohio, USA, [91]Institute of Epidemiology, PopGen Biobank, Christian-Albrechts-University Kiel, Kiel, Germany, [92]Department of Clinical Genetics, Karolinska University Hospital, Stockholm, Sweden, [93]Department of Molecular Medicine and Surgery, Karolinska Institutet, Stockholm, Sweden, [94]Department of Health Science Research, Mayo Clinic, Rochester, MN, USA, [95]Office of Human Research, Taipei Medical University, Taipei, Taiwan, [96]Unit of Oncology 1 - Department of Oncology, Istituto Oncologico Veneto, IRCCS Padua, Italy, [97]Sino-American Cancer Foundation, Covina, CA, USA, [98]Department of Public Health Solutions, National Institute for Health and Welfare, Helsinki, Finland, [99]Biomedicine Institute (IBIOMED), University of León, León, Spain, [100]Cancer Risk Factors and Life-Style Epidemiology Unit, Institute of Cancer Research, Prevention and Clinical Network - ISPRO, Florence, Italy, [101]The Steve and Cindy Rasmussen Institute for Genomic Medicine, Nationwide Children's Hospital, Columbus, OH, USA, [102]University of Michigan Comprehensive Cancer Center, Ann Arbor, Michigan 48105, USA, [103]Candiolo Cancer Institute, FPO-IRCCS, Candiolo, Torino, Italy, [104]Italian Institute for Genomic Medicine (IIGM), Turin, Italy, [105]Department of Genome Sciences, University of Washington, Seattle, Washington, USA, [106]Clinical Genetics Service, Department of Medicine, Memorial Sloan-Kettering Cancer Center, New York, New York, USA, [107]Department of Medicine, Weill Cornell Medical College, New York, New York, USA, [108]The Clinical Epidemiology Unit, Memorial University Medical School, Newfoundland, Canada, [109]Université de Paris, CNRS, MAP5 UMR 8145, F-75006 Paris, France,[110]CESP (Inserm U1018), Facultés de Medicine Université Paris-Sud, UVSQ, Université Paris-Saclay, Gustave Roussy, Villejuif, France, [111]Centre for Public Health Research, Massey University, Wellington, New Zealand, [112]Department of Genetics and Genome Sciences, Case Western Reserve University, Cleveland, Ohio, USA, [113]Department of General Surgery, University Hospital Rostock, Rostock, Germany, [114]Genomic Medicine Institute, Cleveland Clinic, Cleveland, Ohio, USA, [115]Department of Medicine and Epidemiology, University of Pittsburgh Medical Center, Pittsburgh, Pennsylvania, USA, [116]Department of Population and Quantitative Health Sciences, Case Western Reserve University, Cleveland, Ohio, USA, [117]Division of Cancer Control and Population Sciences, National Cancer Institute, Bethesda, Maryland, USA, [118]Centre for Cancer Genetic Epidemiology, Department of Oncology, University of Cambridge, Cambridge, UK, [119]Department of Surgery, University of Tennessee Health Science Center, Memphis, TN, USA, [120]Department of Preventive Medicine, Chonnam National University Medical School, Gwangju, Korea, [121]Oncology Unit, Hillel Yaffe Medical Center, Hadera, Israel, [122]Cancer Epidemiology Program, H, Lee Moffitt Cancer Center and Research Institute, Tampa, FL, USA, [123]Epidemiology and Prevention Unit, Fondazione IRCCS Istituto Nazionale dei Tumori, Milan, Italy, [124]Department of Internal Medicine, University of Utah, Salt Lake City, Utah, USA, [125]Genetic Epidemiology Laboratory, Department of Pathology, The University of Melbourne, Melbourne, Australia, [126]Department of Hematology and Oncology, University of Munich (LMU), Munich, Germany, [127]Kaiser Permanente Washington Health Research Institute, Seattle, Washington, USA, [128]Division of Human Nutrition and Health, Wageningen University & Research, Wageningen, The Netherlands, [129]National Institute for Public Health and the Environment (RIVM), Bilthoven, The Netherlands, [130]Department of Molecular Biology of Cancer, Institute of Experimental Medicine of the Czech Academy of Sciences, Prague, Czech Republic, [131]Institute of Biology and Medical Genetics, First Faculty of Medicine, Charles University, Prague, Czech Republic, [132]Faculty of Medicine and Biomedical Center in Pilsen, Charles University, Pilsen, Czech Republic, [133]Medical Faculty, University of Heidelberg, Germany, [134]School of Medicine, University of Dundee, Dundee, Scotland, [135]Memorial University of Newfoundland, Discipline of Genetics, St, John's, Canada, [136]University of Ottawa, Division of Hematology, Ottawa, Canada

Endometrial Cancer Association Consortium (ECAC) Members

Frederic Amant[1], Daniela Annibali[1], Katie Ashton[2-4], John Attia[2, 5], Paul L. Auer[6, 7], Matthias W. Beckmann[8], Amanda Black[9], Louise Brinton[9], Daniel D. Buchanan[10-13], Stephen J. Chanock[14], Chu Chen[15], Maxine M. Chen[16], Timothy H.T. Cheng[17], Linda S. Cook[18, 19], Marta Crous-Bous[16, 20], Kamila Czene[21], Immaculata De Vivo[16, 20], Joe Dennis[22], Thilo Dörk[23], Sean C. Dowdy[24], Alison M. Dunning[25], Matthias Dürst[26], Douglas F. Easton[22, 25], Arif B. Ekici[27], Peter A. Fasching[8, 28], Brooke L. Fridley[29], Christine M. Friedenreich[19], Montserrat García-Closas[14], Mia M. Gaudet[30], Graham G. Giles[11, 31, 32], Dylan M. Glubb[33], Ellen L. Goode[34], Christopher A. Haiman[35], Per Hall[21, 36], Susan E. Hankinson[20, 37], Catherine S. Healey[25], Alexander Hein[8], Peter Hillemanns[23], Shirley Hodgson[38], Erling Hoivik[39, 40], Elizabeth G. Holliday[2, 5], David J. Hunter[16, 41], Angela Jones[17], Peter Kraft[16, 42], Camilla Krakstad[39, 40], Diether Lambrechts[43, 44], Loic Le Marchand[45], Xiaolin Liang[46], Annika Lindblom[47, 48], Jolanta Lissowska[49], Jirong Long[50], Lingeng Lu[51], Anthony M. Magliocco[52], Lynn Martin[53], Mark McEvoy[5], Roger L. Milne[11, 31, 32], Miriam Mints[54], Rami Nassir[55], Tracy A. O'Mara[33], Irene Orlow[46], Geoffrey Otton[56], Claire Palles[17], Paul D.P. Pharoah[22, 25], Loreall Pooler[35], Tony Proietto[56], Timothy R. Rebbeck[57, 58], Stefan P. Renner[59], Harvey A. Risch[51], Matthias Rübner[59], Ingo Runnebaum[26], Carlotta Sacerdote[60, 61], Gloria E. Sarto[62], Fredrick Schumacher[63], Rodney J. Scott[2, 4, 64], V. Wendy Setiawan[35], Mitul Shah[25], Xin Sheng[35], Xiao-Ou Shu[50], Melissa C. Southey[10, 31, 32], Amanda B. Spurdle[33], Emma Tham[47, 65], Deborah J. Thompson[22], Ian Tomlinson[17, 53], Jone Trovik[39, 40], Constance Turman[16], David Van Den Berg[35], Zhaoming Wang[9], Penelope M. Webb[66], Nicolas Wentzensen[9], Stacey J. Winham[67], Lucy Xia[35], Yong-Bing Xiang[68], Hannah P. Yang[9], Herbert Yu[45], Wei Zheng[50]

Affliations: [1] Department of Obstetrics and Gynecology, Division of Gynecologic Oncology, University Hospitals KU Leuven, University of Leuven, Leuven, Belgium. [2] Hunter Medical Research Institute, John Hunter Hospital, Newcastle, New South Wales, Australia. [3] Centre for Information Based Medicine, University of Newcastle, Callaghan, New South Wales, Australia. [4] Discipline of Medical Genetics, School of Biomedical Sciences and Pharmacy, Faculty of Health, University of Newcastle, Callaghan, New South Wales, Australia. [5] Centre for Clinical Epidemiology and Biostatistics, School of Medicine and Public Health, University of Newcastle, Callaghan, New South Wales, Australia. [6] Cancer Prevention Program, Fred Hutchinson Cancer Research Center, Seattle, WA, USA. [7] Zilber School of Public Health, University of Wisconsin-Milwaukee, Milwaukee, WI, USA. [8] Department of Gynecology and Obstetrics, Comprehensive Cancer Center ER-EMN, University Hospital Erlangen, Friedrich-Alexander-University Erlangen-Nuremberg, Erlangen, Germany. [9] Division of Cancer Epidemiology and Genetics, National Cancer Institute, Bethesda, MD, USA. [10] Department of Clinical Pathology, The University of Melbourne, Melbourne, Victoria, Australia. [11] Centre for Epidemiology and Biostatistics, Melbourne School of Population and Global Health, The University of Melbourne, Melbourne, Victoria, Australia. [12] Genomic Medicine and Family Cancer Clinic, Royal Melbourne Hospital, Parkville, Victoria, Australia. [13] University of Melbourne Centre for Cancer Research, Victorian Comprehensive Cancer Centre, Parkville, Victoria, Australia. [14] Division of Cancer Epidemiology and Genetics, National Cancer Institute, National Institutes of Health, Department of Health and Human Services, Bethesda, MD, USA. [15] Epidemiology Program, Fred Hutchinson Cancer Research Center, Seattle, WA, USA. [16] Department of Epidemiology, Harvard T.H. Chan School of Public Health, Boston, MA, USA. [17] Wellcome Trust Centre for Human Genetics and Oxford NIHR Biomedical Research Centre, University of Oxford, Oxford, UK. [18] University of New Mexico Health Sciences Center, University of New Mexico, Albuquerque, NM, USA. [19] Department of Cancer Epidemiology and Prevention Research, Alberta Health Services, Calgary, AB, Canada. [20] Channing Division of Network Medicine, Department of Medicine, Brigham and Women's Hospital and Harvard Medical School, Boston, MA, USA. [21] Department of Medical Epidemiology and Biostatistics, Karolinska Institutet, Stockholm, Sweden. [22] Centre for Cancer Genetic Epidemiology, Department of

Public Health and Primary Care, University of Cambridge, Cambridge, UK. [23] Gynaecology Research Unit, Hannover Medical School, Hannover, Germany. [24] Department of Obstetrics and Gynecology, Division of Gynecologic Oncology, Mayo Clinic, Rochester, MN, USA. [25] Centre for Cancer Genetic Epidemiology, Department of Oncology, University of Cambridge, Cambridge, UK. [26] Department of Gynaecology, Jena University Hospital - Friedrich Schiller University, Jena, Germany. [27] Institute of Human Genetics, University Hospital Erlangen, Friedrich-Alexander University Erlangen-Nuremberg, Comprehensive Cancer Center Erlangen-EMN, Erlangen, Germany. [28] David Geffen School of Medicine, Department of Medicine Division of Hematology and Oncology, University of California at Los Angeles, Los Angeles, CA, USA. [29] Department of Biostatistics, Kansas University Medical Center, Kansas City, KS, USA. [30] Department of Population Science, American Cancer Society, Atlanta, GA, USA. [31] Cancer Epidemiology Division, Cancer Council Victoria, Melbourne, Victoria, Australia. [32] Precision Medicine, School of Clinical Sciences at Monash Health, Monash University, Clayton, Victoria, Australia. [33] Department of Genetics and Computational Biology, QIMR Berghofer Medical Research Institute, Brisbane, Queensland, Australia. [34] Department of Health Science Research, Division of Epidemiology, Mayo Clinic, Rochester, MN, USA. [35] Department of Preventive Medicine, Keck School of Medicine, University of Southern California, Los Angeles, CA, USA. [36] Department of Oncology, Södersjukhuset, Stockholm, Sweden. [37] Department of Biostatistics & Epidemiology, University of Massachusetts, Amherst, Amherst, MA, USA. [38] Department of Clinical Genetics, St George's, University of London, London, UK. [39] Centre for Cancer Biomarkers CCBIO, Department of Clinical Science, University of Bergen, Bergen, Norway. [40] Department of Obstetrics and Gynecology, Haukeland University Hospital, Bergen, Norway. [41] Nuffield Department of Population Health, University of Oxford, Oxford, UK. [42] Program in Genetic Epidemiology and Statistical Genetics, Harvard T.H. Chan School of Public Health, Boston, MA, USA. [43] VIB Center for Cancer Biology, Leuven, Belgium. [44] Laboratory for Translational Genetics, Department of Human Genetics, University of Leuven, Leuven, Belgium. [45] Epidemiology Program, University of Hawaii Cancer Center, Honolulu, HI, USA. [46] Department of Epidemiology and Biostatistics, Memorial Sloan-Kettering Cancer Center, New York, NY, USA. [47] Department of Molecular Medicine and Surgery, Karolinska Institutet, Stockholm, Sweden. [48] Department of Clinical Genetics, Karolinska University Hospital, Stockholm, Sweden. [49] Department of Cancer Epidemiology and Prevention, M. Sklodowska-Curie Cancer Center, Oncology Institute, Warsaw, Poland. [50] Division of Epidemiology, Department of Medicine, Vanderbilt Epidemiology Center, Vanderbilt-Ingram Cancer Center, Vanderbilt University School of Medicine, Nashville, TN, USA. [51] Chronic Disease Epidemiology, Yale School of Public Health, New Haven, CT, USA. [52] Department of Anatomic Pathology, Moffitt Cancer Center & Research Institute, Tampa, FL, USA. [53] Institute of Cancer and Genomic Sciences, University of Birmingham, Birmingham, UK. [54] Department of Women's and Children's Health, Karolinska Institutet, Stockholm, Sweden. [55] Department of Biochemistry and Molecular Medicine, University of California Davis, Davis, CA, USA. [56] School of Medicine and Public Health, University of Newcastle, Callaghan, New South Wales, Australia. [57] Harvard T.H. Chan School of Public Health, Boston, MA, USA. [58] Dana-Farber Cancer Institute, Boston, MA, USA. [59] Department of Gynaecology and Obstetrics, University Hospital Erlangen, Friedrich-Alexander University Erlangen-Nuremberg, Comprehensive Cancer Center Erlangen-EMN, Erlangen, Germany. [60] Center for Cancer Prevention (CPO-Peimonte), Turin, Italy. [61] Human Genetics Foundation (HuGeF), Turino, Italy. [62] Department of Obstetrics and Gynecology, School of Medicine and Public Health, University of Wisconsin, Madison, WI, USA. [63] Department of Population and Quantitative Health Sciences, Case Western Reserve University, Cleveland, OH, USA. [64] Division of Molecular Medicine, Pathology North, John Hunter Hospital, Newcastle, New South Wales, Australia. [65] Clinical Genetics, Karolinska Institutet, Stockholm, Sweden. [66] Population Health Department, QIMR Berghofer Medical Research Institute, Brisbane, Queensland, Australia. [67] Department of Health Sciences Research, Division of Biomedical Statistics and Informatics, Mayo Clinic, Rochester, MN, USA. [68] State Key Laboratory of Oncogene and Related Genes

& Department of Epidemiology, Shanghai Cancer Institute, Renji Hospital, Shanghai Jiaotong University School of Medicine, Shanghai, China.

Esophageal Cancer GWAS Consortium Members

Puya Gharahkhania[1], Rebecca C Fitzgerald[2], Thomas L Vaughan[3], Claire Palles[4], Ines Gockel[5], Ian Tomlinson[4], Matthew F Buas[3], Andrea May[6], Christian Gerges[7], Mario Anders[8,9], Jessica Becker[10], Nicole Kreuser[11], Tania Noder[8], Marino Venerito[12], Lothar Veits[13], Thomas Schmidt[14], Hendrik Manner[15], Claudia Schmidt[16], Timo Hess[10], Anne C Böhmer[10], Jakob R Izbicki[17], Arnulf H Hölscher[16], Hauke Lang[18], Dietmar Lorenz[19], Brigitte Schumacher[7,20], Andreas Hackelsberger[21], Rupert Mayershofer[22], Oliver Pech[23], Yogesh Vashist[17,24], Katja Ott[14,25], Michael Vieth[13], Josef Weismüller[26], Markus M Nöthen[10], Barrett's and Esophageal Adenocarcinoma Consortium (BEACON), Esophageal Adenocarcinoma GenEtics Consortium (EAGLE), Wellcome Trust Case Control Consortium 2 (WTCCC2), Stephen Attwood[27], Hugh Barr[28], Laura Chegwidden[29], John de Caestecker[30], Rebecca Harrison[31], Sharon B Love[5], David MacDonald[32], Paul Moayyedi[33], Hans Prenen[34], R G Peter Watson[35], Prasad G Iyer[36], Lesley A Anderson[37], Leslie Bernstein[38], Wong-Ho Chow[39], Laura J Hardie[40], Jesper Lagergren[41,42], Geoffrey Liu[43], Harvey A Risch[44], Anna H Wu[45], Weimin Ye[46], Nigel C Bird[47], Nicholas J Shaheen[48], Marilie D Gammon[49], Douglas A Corley[50], Carlos Caldas[51], Susanne Moebus[52], Michael Knapp[53], Wilbert H M Peters[54], Horst Neuhaus[7], Thomas Rösch[8], Christian Ell[6], Stuart MacGregor[1], Paul Pharoah[55], David C Whiteman[56], Janusz Jankowski, Prof[57,58], Johannes Schumacher[10]

Affiliations: [1]Statistical Genetics, QIMR Berghofer Medical Research Institute, Brisbane, QLD, Australia; [2]Medical Research Council (MRC) Cancer Unit, Hutchison-MRC Research Centre and University of Cambridge, Cambridge, UK; [3]Division of Public Health Sciences, Fred Hutchinson Cancer Research Center, Seattle, WA, USA; [4]Wellcome Trust Centre for Human Genetics, University of Oxford, Oxford, UK; [5]Centre for Statistics in Medicine, NDORMS, University of Oxford, Oxford, UK; [6]Department of Medicine II, Sana Klinikum, Offenbach, Germany; [7]Department of Internal Medicine, Evangelisches Krankenhaus, Düsseldorf, Germany; [8]Department of Interdisciplinary Endoscopy, University Hospital Hamburg-Eppendorf, Hamburg, Germany; [9]Department of Gastroenterology and Interdisciplinary Endoscopy, Vivantes Wenckebach-Klinikum, Berlin, Germany; [10]Institute of Human Genetics, and Department of Genomics, Life & Brain Center, University of Bonn, Bonn, Germany; [11]Department of Visceral, Transplant, Thoracic and Vascular Surgery, University Hospital of Leipzig, Leipzig, Germany; [12]Department of Gastroenterology, Hepatology and Infectious Diseases, Otto-von-Guericke University Hospital, Magdeburg, Germany; [13]Institute of Pathology, Klinikum Bayreuth, Bayreuth, Germany; [14]Department of General, Visceral and Transplantation Surgery, University of Heidelberg, Heidelberg, Germany; [15]Department of Internal Medicine II, Horst Schmidt Kliniken Hospital, Wiesbaden, Germany; [16]Department of General, Visceral and Cancer Surgery, University of Cologne, Cologne, Germany; [17]Department of General, Visceral and Thoracic Surgery, University Medical Center Hamburg-Eppendorf, University of Hamburg, Hamburg, Germany; [18]Department of General, Visceral and Transplant Surgery, University Medical Center, University of Mainz, Mainz, Germany; [19]Department of General and Visceral Surgery, Sana Klinikum, Offenbach, Germany; [20]Department of Internal Medicine and Gastroenterology, Elisabeth Hospital, Essen, Germany; [21]Gastropraxis, Wiesbaden, Germany; [22]Gastroenterologie am Burgweiher, Bonn, Germany; [23]Department of Gastroenterology and Interventional Endoscopy, St John of God Hospital, Regensburg, Germany; [24]Department of Visceral Surgery, Kantonsspital Aarau AG, Aarau, Switzerland; [25]Department of General, Visceral and Thorax Surgery, RoMed Klinikum Rosenheim, Rosenheim, Germany; [26]Gastroenterologische Gemeinschaftspraxis, Koblenz, Germany; [27]Centre For Integrated Health Care Research, Durham University, Durham, UK; [28]Gloucestershire Royal Hospital, Gloucester, UK; [29]Plymouth University Peninsula School of Medicine and Dentistry, Plymouth, UK; [30]Digestive Diseases Centre, University Hospitals of Leicester, Leicester, UK; [31]Department of Cellular Pathology, Leicester Royal Infirmary, Leicester, UK; [32]Department of Oral Biological and Medical Sciences, University of British Columbia, Vancouver, BC, Canada; [33]Department of Medicine, McMaster

University, Hamilton, ON, Canada; [34]Department of Gastroenterology, University Hospitals Gasthuisberg, Leuven, Belgium; [35]Queen's University Belfast, Centre of Medical Education, Royal Victoria Hospital, Belfast, UK; [36]Division of Gastroenterology and Hepatology, Department of Internal Medicine, Mayo Clinic, Rochester, MN, USA; [37]Centre for Public Health, Queen's University Belfast, Belfast, UK; [38]Department of Population Sciences, Beckman Research Institute and City of Hope Comprehensive Cancer Center, Duarte, CA, USA; [39]Department of Epidemiology, MD Anderson Cancer Center, Houston, TX, USA; [40]Division of Epidemiology, University of Leeds, Leeds, UK; [41]Department of Molecular Medicine and Surgery, Karolinska Institute, Stockholm, Sweden; [42]Division of Cancer Studies, King's College London, London, UK; [43]Pharmacogenomic Epidemiology, Ontario Cancer Institute, Toronto, ON, Canada; [44]Department of Chronic Disease Epidemiology, Yale School of Public Health, New Haven, CT, USA; [45]Department of Preventive Medicine, University of Southern California/Norris Comprehensive Cancer Center, Los Angeles, CA, USA; [46]Department of Medical Epidemiology and Biostatistics, Karolinska Institute, Stockholm, Sweden; [47]Department of Oncology, Medical School, University of Sheffield, Sheffield, UK; [48]Division of Gastroenterology and Hepatology, University of North Carolina School of Medicine, University of North Carolina, Chapel Hill, North Carolina, USA; [49]Department of Epidemiology, University of North Carolina, Chapel Hill, North Carolina, USA; [50]Division of Research, and San Francisco Medical Center, Kaiser Permanente Northern California, Oakland, CA, USA; [51]Department of Oncology, and Cancer Research UK Cambridge Institute, University of Cambridge, Cambridge, UK; [52]Centre of Urban Epidemiology, Institute of Medical Informatics, Biometry and Epidemiology, University of Essen, Essen, Germany; [53]Institute for Medical Biometry, Informatics, and Epidemiology, University of Bonn, Bonn, Germany; [54]Department of Gastroenterology, Radboud University Nijmegen Medical Center, Nijmegen, Netherlands; [55]Centre for Cancer Genetic Epidemiology, Department of Oncology, University of Cambridge, Cambridge, UK; [56]Cancer Control, QIMR Berghofer Medical Research Institute, Brisbane, QLD, Australia; [57]University of Central Lancashire, Westlakes Science and Technology Park, Moor Row, UK; [58]Warwick Medical School, University of Warwick, Warwick, UK;

Glioma International Case Control Consortium (GICC) Members

Melissa L. Bondy[1], Ryan T. Merrell[2,], Daniel Lachance[3,], Georgina N. Armstrong[1], Margaret R. Wrensch[5], Dora Il'yasova[7,] Elizabeth B. Claus[9], Jill S. Barnholtz-Sloan[10], Joellen Schildkraut[11], Siegal Sadetzki[12,13], Christoffer Johansen[14,15], Richard S. Houlston[17,16], Robert B. Jenkins[17], Jonine L. Bernstein[6], Rose Lai[18], Sanjay Shete[19], Christopher I. Amos[20], and Beatrice S. Melin[21]

Affiliations: [1]Department of Epidemiology and Population Health, Stanford Cancer Institute, Stanford Medicine, Stanford University, Palo Alto, California [2]Department of Neurology, NorthShore University HealthSystem, Evanston, Illinois; [3]Department of Neurology, Mayo Clinic Comprehensive Cancer Center, Mayo Clinic, Rochester, Minnesota; [5]Department of Neurological Surgery, University of California, San Francisco, San Francisco, California; [6]Department of Epidemiology and Biostatistics, Memorial Sloan-Kettering Cancer Center, New York, New York; [7]Department of Epidemiology and Biostatistics, Georgia State University School of Public Health, Atlanta, Georgia; [8]Department of Pediatrics, Dan L. Duncan Cancer Center, Baylor College of Medicine, Houston, Texas; [9]Department of Epidemiology and Public Health, Yale University School of Medicine, New Haven, Connecticut; [10]Case Comprehensive Cancer Center, Case Western Reserve University School of Medicine, Cleveland, Ohio; [11]Department of Epidemiology, Rollins School of Public Health, Emory University, Atlanta, Georgia; [12]Cancer and Radiation Epidemiology Unit, Gertner Institute, Chaim Sheba Medical Center, Tel Hashomer, Israel; [13]Sackler School of Medicine, Tel-Aviv University, Tel-Aviv, Israel; [14]Institute of Cancer Epidemiology, Danish Cancer Society, Copenhagen, Denmark; [15]Rigshospitalet, University of Copenhagen, Copenhagen, Denmark; [16]Section of Cancer Genetics, Institute of Cancer Research, Sutton, Surrey, United Kingdom; [17]Department of Laboratory Medicine and Pathology, Mayo Clinic Comprehensive Cancer Center, Mayo Clinic, Rochester, Minnesota; [18]Departments of Neurology, Neurosurgery, and Preventive Medicine, The University of Southern California Keck School of Medicine, Los Angeles, California; [19]Department of Biostatistics, The University of Texas MD Anderson Cancer Center, Houston, Texas; [20]Division of Medicine, Dan L. Duncan Cancer Center, Baylor College of Medicine, Houston, Texas; [21]Department of Radiation Sciences Oncology, Umeå University, Umeå, Sweden

Head and Neck Cancer GWAS Consortium Members

Corina Lesseur,[1] Brenda Diergaarde,[2,3] Andrew F Olshan,[4,5] Victor Wünsch-Filho,[6] Andrew R Ness,[7,8] Geoffrey Liu,[9] Martin Lacko,[10] José Eluf-Neto,[11] Silvia Franceschi,[1] Pagona Lagiou,[12] Gary J Macfarlane,[13] Lorenzo Richiardi,[14] Stefania Boccia,[15] Jerry Polesel,[16] Kristina Kjaerheim,[17] David Zaridze,[18] Mattias Johansson,[1] Ana M Menezes,[19] Maria Paula Curado,[20] Max Robinson,[21] Wolfgang Ahrens,[22] Cristina Canova,[23] Ariana Znaor,[1],[24] Xavier Castellsagué,[25] David I Conway,[26],[27] Ivana Holcátová,[28] Dana Mates,[29] Marta Vilensky,[30] Claire M Healy,[31] Neonila Szeszenia-Dąbrowska,[32] Eleonóra Fabiánová,[33] Jolanta Lissowska,[34] Jennifer R Grandis,[35],[36] Mark C Weissler,[37] Eloiza H Tajara,[38] Fabio D Nunes,[39] Marcos B de Carvalho,[40] Steve Thomas,[8] Rayjean J Hung,[41] Wilbert H M Peters,[42] Rolando Herrero,[1] Gabriella Cadoni,[44] H Bas Bueno-de-Mesquita,[44,45,46] Annika Steffen,[47] Antonio Agudo,[48] Oxana Shangina,[18] Xiangjun Xiao,[49] Valérie Gaborieau,[1] Amélie Chabrier,[1] Devasena Anantharaman,[1] Paolo Boffetta,[50] Christopher I Amos,[49] James D McKay,[1] Paul Brennan[1]

Affiliations: [1]International Agency for Research on Cancer (IARC/WHO), Lyon, France, [2]Department of Epidemiology, Graduate School of Public Health, University of Pittsburgh, Pittsburgh, PA, USA, [3]University of Pittsburgh Cancer Institute, Pittsburgh, PA, USA, [4]Department of Epidemiology, Gillings School of Global Public Health, University of North Carolina, NC, USA, [5]UNC Lineberger Comprehensive Cancer Center, Chapel Hill, NC, USA, [6]Faculdade de Saúde Pública, Universidade de São Paulo, São Paulo, SP, Brazil, [7]National Institute for Health Research (NIHR) Bristol Biomedical Research Centre, University Hospitals Bristol and Weston NHS Foundation Trust and the University of Bristol, UK, [8]Bristol Dental School, University of Bristol, Bristol, UK, [9]Princess Margaret Cancer Centre, Toronto, Ontario, Canada, [10]Department of Otorhinolaryngology, Head and Neck Surgery, Maastricht University Medical Center, Maastricht, Netherlands, [11]Departamento de Medicina Preventiva, Faculdade de Medicina da Universidade de São Paulo, São Paulo, Brazil, [12]Department of Hygiene, Epidemiology and Medical Statistics, School of Medicine, National Kapodistrian University of Athens, Athens, Greece, [13]School of Medicine, Medical Sciences and Nutrition, University of Aberdeen, Aberdeen, UK, [14]Department of Medical Sciences, University of Turin, Turin, Italy, [15]Section of Hygiene, Institute of Public Health, Università Cattolica del Sacro Cuore, Fondazione Policlinico 'Agostino Gemelli', Rome, Italy, [16]Unit of Cancer Epidemiology, CRO Aviano National Cancer Institute, Aviano, Italy, [17]Cancer Registry of Norway, Oslo, Norway, [18]Department of Cancer Epidemiology and Prevention, Institute of Carcinogenesis, N.N. Blokhin Russian Cancer Research Centre of the Russian Ministry of Health, Moscow, Russian Federation, [19]Programa de Pós-Graduacão em Epidemiologia, Universidade Federal de Pelotas (UFPel), Pelotas, Brazil, [20]Epidemiology, International Center for Research (CIPE), A.C.Camargo Cancer Center, Sao Paulo, Brazil, [21]Centre for Oral Health Research, Newcastle University, Newcastle, UK, [22]Leibniz Institute for Prevention Research and Epidemiology - BIPS, Bremen, Germany, [23]Deparment of Molecular Medicine, University of Padova, Padova, Italy, [24]Croatian National Institute of Public Health, Zagreb, Croatia, [25]Institut Català d'Oncologia (ICO)-DIBELL, CIBER-ESP, L'Hospitalet de Llobregat, Catalonia, Spain, [26]School of Medicine, Dentistry and Nursing, University of Glasgow, University of Glasgow, Glasgow, UK, [27]NHS NSS National Services Scotland, Edinburgh, UK, [28]Institute of Hygiene & Epidemiology 1st Faculty of Medicine, Charles University, Prague, Czech Republic, [29]National Institute of Public Health, Bucharest, Romania, [30]Instituto de Oncologia "Angel H Roffo", Universidad de Buenos Aires, Buenos Aires, Argentina, [31]Trinity College School of Dental Science, Dublin, Ireland, [32]Department of Environmental Epidemiology, Nofer Institute of Occupational Medicine, Lodz, Poland, [33]Regional Authority of Public Health, Banská Bystrica, Slovakia, [34]The Maria Skłodowska-Curie Memorial Cancer Centre and Institute of Oncology (MCMCC), Warsaw, Poland, [35]Department of Otolaryngology-Head and Neck Surgery, University of California San Francisco, San Francisco, California, USA, [36]Clinical Translational Science Institute, University of California at San Francisco, San Francisco, CA, USA, [37]Department of

Otolaryngology/Head and Neck Surgery, University of North Carolina at Chapel Hill, Chapel Hill, NC, USA, [38]Department of Molecular Biology, School of Medicine of São José do Rio Preto, São José do Rio Preto, SP, Brazil, [39]Department of Stomatology, School of Dentistry, University of São Paulo, São Paulo, SP, Brazil, [40]Department of Head and Neck Surgery, Heliópolis Hospital, São Paulo, SP, Brazil, [41]Lunenfeld-Tanenbaum Research Institute, Mount Sinai Hospital, Toronto, Ontario, Canada, [42]Department of Gastroenterology, Radboud University Nijmegen, Medical Center, Nijmegen, the Netherlands, [43]Institute of Otorhinolaryngology, Università Cattolica del Sacro Cuore, Fondazione Policlinico 'Agostino Gemelli', Rome, Italy, [44]Department for Determinants of Chronic Diseases (DCD), National Institute for Public Health and the Environment (RIVM), Bilthoven, Netherlands, [45]Department of Epidemiology and Biostatistics, School of Public Health, Imperial College London, London, UK, [46]Department of Social and Preventive Medicine, Faculty of Medicine, University of Malaya, Kuala Lumpur, Malaysia, [47]German Institute of Human Nutrition in Potsdam-Rehbruecke (DIfE), Nuthetal, Germany, [48]Unit of Nutrition and Cancer, Cancer Epidemiology Research Program, Institut Català d'Oncologia (ICO)-IDIBELL, l'Hospitalet de Llobregat, Barcelona, Spain, [49]Department of Community and Family Medicine, Geisel School of Medicine, Dartmouth College, Lebanon, New Hampshire, USA, [50]Tisch Cancer Institute, Icahn School of Medicine at Mount Sinai, New York, New York, USA

International Lung Cancer Consortium (ILCCO) Members

Demetrios Albanes[1], Melinda C. Aldrich[2], Christopher I. Amos[3], Angeline Andrew[4], Susanne Arnold[5], Heike Bickeböller[6], Stig E. Bojesen[7], Paul Brennan[8], Hans Brunnström[9], Neil Caporaso[1], Chu Chen[10], David Christiani[11], John K. Field[12], Kjell Grankvist[13], Rayjean J. Hung[14], Mattias Johansson[8,13], Lambertus A. Kiemeney[15], Stephen Lam[16], Maria Teresa Landi[1], Philip Lazarus[17], Geoffrey Liu[18], Loic Le Marchand[19], Olle Melander[9], Gadi Rennert[20], Angela Risch[21], Matthew B. Schabath[22], Sanjay S. Shete[23], Adonina Tardon[24], M. Dawn Teare[25], H.-Erich Wichmann[26], Shan Zienolddiny[27]

Affiliations: [1]Division of Cancer Epidemiology and Genetics, National Cancer Institute, National Institutes of Health, Bethesda, Maryland, USA, [2]Department of Thoracic Surgery, Division of Epidemiology, Vanderbilt University Medical Center, Nashville, TN, USA, [3]Institute for Clinical and Translational Research, Baylor Medical College, Houston, TX, USA, [4]Norris Cotton Cancer Center, Dartmouth Geisel School of Medicine, Lebanon, NH, USA, [5]Markey Cancer Center, University of Kentucky, Lexington, KY, USA, [6]Department of Genetic Epidemiology, University Medical Center Göttingen, Gottingen, Germany, [7]Department of Clinical Biochemistry, Herlev and Gentofte Hospital, Copenhagen University Hospital, Denmark, [8]International Agency for Research on Cancer, Lyon, France, [9]Department of Clinical Sciences, Lund University, Malmo, Sweden, [10]Public Health Sciences Division, Fred Hutchinson Cancer Research Center, Seattle, WA, USA, [11]Department of Epidemiology, Harvard T.H. Chan School of Public Health, Boston, Massachusetts, USA, [12]Roy Castle Lung Cancer Research Programme, The University of Liverpool, Liverpool, UK, [13]Department of Medical Biosciences, Umeå University, Umeå, Sweden, [14]Sinai Health System, Lunenfeld-Tanenbaum Research Institute, Toronto, Canada, [15]Radboud university medical center, Nijmegen, the Netherlands, [16]British Columbia Cancer Agency, Vancouver, Canada, [17]College of Pharmacy, Washington State University, Spokane, WA, USA, [18]Princess Margaret Cancer Centre, University Health Network, Toronto, Canada, [19]Cancer Epidemiology Program, University of Hawaii Cancer Center at Manoa, Honolulu, HI, USA, [20]Carmel Medical Center, Faculty of Medicine, Israel Institute of Technology, Haifa, Israel, [21]Department of Molecular Biology, Division of Epigenomics and Cancer Risk Factors, German Cancer Research Center (DKFZ), Heidelberg, Germany, [22]Department of Cancer Epidemiology, H. Lee Moffitt Cancer Center and Research Institute, Tampa, FL, USA, [23]Department of Epidemiology, Division of Cancer Prevention and Population Science, MD Anderson Cancer Center, Houston, Texas, USA, [24]CIBER Epidemiología y Salud Pública (CIBERESP), Madrid, Spain, [25]School of Health and Related Research (ScHARR), University of Sheffield, Sheffield, UK, [26]Institute of Medical Informatics, Biometry and Epidemiology, Chair of Epidemiology, Ludwig Maximilians University, Munich, Germany, [27]National Institute of Occupational Health (STAMI), Oslo, Norway

Melanoma GWAS consortium Members

Matthew H Law[1], D Timothy Bishop[2], Jeffrey E Lee[3], Myriam Brossard[4,5], Nicholas G Martin[6], Eric K Moses[7], Fengju Song[8], Jennifer H Barrett[2], Rajiv Kumar[9], Douglas F Easton[10], Paul D P Pharoah[11], Anthony J Swerdlow[12,13], Katerina P Kypreou[14], John C Taylor[2], Mark Harland[2], Juliette Randerson-Moor[2], Lars A Akslen[15,16], Per A Andresen[17], Marie-Françoise Avril[18], Esther Azizi[19,20], Giovanna Bianchi Scarrà[21,22], Kevin M Brown[23], Tadeusz Dębniak[24], David L Duffy[6], David E Elder[25], Shenying Fang[3], Eitan Friedman[20], Pilar Galan[26], Paola Ghiorzo[21,22], Elizabeth M Gillanders[27], Alisa M Goldstein[23], Nelleke A Gruis[28], Johan Hansson[29], Per Helsing[30], Marko Hočevar[31], Veronica Höiom[29], Christian Ingvar[32], Peter A Kanetsky[33], Wei V Chen[34], GenoMEL Consortium, Essen-Heidelberg Investigators, The SDH Study Group, Q-MEGA and QTWIN Investigators, AMFS Investigators, ATHENS Melanoma Study Group, Maria Teresa Landi[23], Julie Lang[35], G Mark Lathrop[36], Jan Lubiński[24], Rona M Mackie[35,37], Graham J Mann[38], Anders Molven[16,39], Grant W Montgomery[40], Srdjan Novaković[41], Håkan Olsson[42,43], Susana Puig[44,45], Joan Anton Puig-Butille[44,45], Abrar A Qureshi[46], Graham L Radford-Smith[47,48,49], Nienke van der Stoep[50], Remco van Doorn[28], David C Whiteman[51], Jamie E Craig[52], Dirk Schadendorf[53,54], Lisa A Simms[47], Kathryn P Burdon[55], Dale R Nyholt[40,56], Karen A Pooley[10], Nick Orr[57], Alexander J Stratigos[14], Anne E Cust[58], Sarah V Ward[7], Nicholas K Hayward[59], Jiali Han[60,61], Hans-Joachim Schulze[62], Alison M Dunning[11], Julia A Newton Bishop[2], Florence Demenais[4,5], Christopher I Amos[63], Stuart MacGregor[1] & Mark M Iles[2]

Affiliations: [1]Statistical Genetics, QIMR Berghofer Medical Research Institute, Brisbane, Queensland, Australia, [2]Section of Epidemiology and Biostatistics, Leeds Institute of Cancer and Pathology, University of Leeds, Leeds, UK, [3]Department of Surgical Oncology, University of Texas MD Anderson Cancer Center, Houston, Texas, USA, [4]INSERM, UMR 946, Genetic Variation and Human Diseases Unit, Paris, France, [5]Institut Universitaire d'Hématologie, Université Paris Diderot, Sorbonne Paris Cité, Paris, France, [6]Genetic Epidemiology, QIMR Berghofer Medical Research Institute, Brisbane, Queensland, Australia, [7]Centre for Genetic Origins of Health and Disease, Faculty of Medicine, Dentistry and Health Sciences, University of Western Australia, Perth, Western Australia, Australia, [8]Department of Epidemiology and Biostatistics, Key Laboratory of Cancer Prevention and Therapy, Tianjin, National Clinical Research Center of Cancer, Tianjin Medical University Cancer Institute and Hospital, Tianjin, China, [9]Division of Molecular Genetic Epidemiology, German Cancer Research Center, Heidelberg, Germany, [10]Department of Public Health and Primary Care, Centre for Cancer Genetic Epidemiology, University of Cambridge, Cambridge, UK, [11]Department of Oncology, Centre for Cancer Genetic Epidemiology, University of Cambridge, Cambridge, UK, [12]Division of Genetics and Epidemiology, The Institute of Cancer Research, London, UK, [13]Division of Breast Cancer Research, The Institute of Cancer Research, London, UK, [14]Department of Dermatology, University of Athens School of Medicine, Andreas Sygros Hospital, Athens, Greece, [15]Department of Clinical Medicine, Centre for Cancer Biomarkers (CCBIO), University of Bergen, Bergen, Norway, [16]Department of Pathology, Haukeland University Hospital, Bergen, Norway, [17]Department of Pathology, Molecular Pathology, Oslo University Hospital, Rikshospitalet, Oslo, Norway, [18]Assistance Publique–Hôpitaux de Paris, Hôpital Cochin, Service de Dermatologie, Université Paris Descartes, Paris, France, [19]Department of Dermatology, Sheba Medical Center, Tel Hashomer, Sackler Faculty of Medicine, Tel Aviv, Israel, [20]Oncogenetics Unit, Sheba Medical Center, Tel Hashomer, Sackler Faculty of Medicine, Tel Aviv University, Tel Aviv, Israel, [21]Department of Internal Medicine and Medical Specialties, University of Genoa, Genoa, Italy, [22]Laboratory of Genetics of Rare Cancers, Istituto di Ricovero e Cura a Carattere Scientifico Azienda Ospedaliera Universitaria (IRCCS AOU) San Martino l'Istituto Scientifico Tumori Istituto Nazionale per la Ricerca sul Cancro, Genoa, Italy, [23]Division of Cancer Epidemiology and Genetics, National Cancer Institute, US National Institutes of Health, Bethesda, Maryland, USA, [24]International Hereditary Cancer Center, Pomeranian Medical University, Szczecin, Poland, [25]Department of Pathology and Laboratory Medicine, Perelman School of Medicine at the University of Pennsylvania, Philadelphia,

Pennsylvania, USA, [26]Université Paris 13, Equipe de Recherche en Epidémiologie Nutritionnelle (EREN), Centre de Recherche en Epidémiologie et Statistiques, INSERM U1153, Institut National de la Recherche Agronomique (INRA) U1125, Conservatoire National des Arts et Métiers, Communauté d'Université Sorbonne Paris Cité, Bobigny, France, [27]Inherited Disease Research Branch, National Human Genome Research Institute, US National Institutes of Health, Baltimore, Maryland, USA, [28]Department of Dermatology, Leiden University Medical Center, Leiden, the Netherlands, [29]Department of Oncology-Pathology, Karolinska Institutet, Karolinska University Hospital, Stockholm, Sweden, [30]Department of Dermatology, Oslo University Hospital, Rikshospitalet, Oslo, Norway, [31]Department of Surgical Oncology, Institute of Oncology Ljubljana, Ljubljana, Slovenia, [32]Department of Surgery, Clinical Sciences, Lund University, Lund, Sweden, [33]Department of Cancer Epidemiology, H. Lee Moffitt Cancer Center and Research Institute, Tampa, Florida, USA, [34]Department of Genetics, University of Texas MD Anderson Cancer Center, Houston, Texas, USA, [35]Department of Medical Genetics, University of Glasgow, Glasgow, UK, [36]McGill University and Génome Québec Innovation Centre, Montreal, Quebec, Canada, [37]Department of Public Health, University of Glasgow, Glasgow, UK, [38]Centre for Cancer Research, University of Sydney at Westmead, Millennium Institute for Medical Research and Melanoma Institute Australia, Sydney, New South Wales, Australia, [39]Department of Clinical Medicine, Gade Laboratory for Pathology, University of Bergen, Bergen, Norway, [40]Molecular Epidemiology, QIMR Berghofer Medical Research Institute, Brisbane, Queensland, Australia, [41]Department of Molecular Diagnostics, Institute of Oncology Ljubljana, Ljubljana, Slovenia, [42]Department of Oncology/Pathology, Clinical Sciences, Lund University, Lund, Sweden, [43]Department of Cancer Epidemiology, Clinical Sciences, Lund University, Lund, Sweden, [44]Departments of Dermatology, Melanoma Unit, Biochemistry and Molecular Genetics, Hospital Clinic, Institut d'Investigacions Biomèdica August Pi Suñe, Universitat de Barcelona, Barcelona, Spain, [45]Centro de Investigación Biomédica en Red (CIBER) de Enfermedades Raras, Instituto de Salud Carlos III, Barcelona, Spain, [46]Department of Dermatology, Warren Alpert Medical School of Brown University, Providence, Rhode Island, USA, [47]Inflammatory Bowel Diseases, QIMR Berghofer Medical Research Institute, Brisbane, Queensland, Australia, [48]Department of Gastroenterology and Hepatology, Royal Brisbane and Women's Hospital, Brisbane, Queensland, Australia, [49]University of Queensland School of Medicine, Herston Campus, Brisbane, Queensland, Australia, [50]Department of Clinical Genetics, Center of Human and Clinical Genetics, Leiden University Medical Center, Leiden, the Netherlands, [51]Cancer Control Group, QIMR Berghofer Medical Research Institute, Brisbane, Queensland, Australia, [52]Department of Ophthalmology, Flinders University, Adelaide, South Australia, Australia, [53]Department of Dermatology, University Hospital Essen, Essen, Germany, [54]German Consortium for Translational Cancer Research (DKTK), Heidelberg, Germany, [55]Menzies Institute for Medical Research, University of Tasmania, Hobart, Tasmania, Australia, [56]Institute of Health and Biomedical Innovation, Queensland University of Technology, Brisbane, Queensland, Australia, [57]Breakthrough Breast Cancer Research Centre, The Institute of Cancer Research, London, UK, [58]Cancer Epidemiology and Services Research, Sydney School of Public Health, University of Sydney, Sydney, New South Wales, Australia, [59]Oncogenomics, QIMR Berghofer Medical Research Institute, Brisbane, Queensland, Australia, [60]Department of Epidemiology, Richard M. Fairbanks School of Public Health, Indiana University, Indianapolis, Indiana, USA, [61]Melvin and Bren Simon Cancer Center, Indiana University, Indianapolis, Indiana, USA, [62]Department of Dermatology, Fachklinik Hornheide, Institute for Tumors of the Skin at the University of Münster, Münster, Germany, [63]Department of Community and Family Medicine, Geisel School of Medicine, Dartmouth College, Hanover, New Hampshire, USA

Ovarian Cancer Association Consortium (OCAC) Members

Hoda Anton-Culver[1], Elisa V. Bandera[2], Susana N Banerjee[3], Javier Benitez[4, 5], Andrew Berchuck[6], Line Bjorge[7, 8], James D. Brenton[9], Ralf Butzow[10], Ian Campbell[11, 12], Kexin Chen[13], Georgia Chenevix-Trench[14], Linda S. Cook[15, 16], Daniel W. Cramer[17, 18], Anna deFazio[19, 20], Jennifer A. Doherty[21], Thilo Dörk[22], Diana M. Eccles[23], Peter A. Fasching[24, 25], Renée T. Fortner[26], Simon A. Gayther[27], Ellen L. Goode[28], Marc T. Goodman[29], Jacek Gronwald[30], Holly R. Harris[31, 32], Florian Heitz[33, 34, 35], Michelle A.T. Hildebrandt[36], Estrid Høgdall[37, 38], Claus K. Høgdall[39], David G. Huntsman[40, 41, 42, 43], Beth Y. Karlan[44], Lambertus A. Kiemeney[45], Susanne K. Kjaer[37, 39], Jolanta Kupryjanczyk[46], Diether Lambrechts[47, 48], Nhu D. Le[49], Douglas A. Levine[50, 51], Keitaro Matsuo[52, 53], Taymaa May[54], Iain A. McNeish[55, 56], Usha Menon[57], Roger L. Milne[58, 59, 60], Francesmary Modugno[61, 62], Alvaro N. Monteiro[63], Patricia G. Moorman[64], Kirsten B. Moysich[65], Heli Nevanlinna66, Håkan Olsson[67], Sue K. Park[68, 69, 70], Celeste L. Pearce[71, 72], Tanja Pejovic[73, 74], Malcolm C. Pike[72, 75], Susan J. Ramus[76, 77], Elio Riboli[78], Marjorie J. Riggan[6], Harvey A. Risch[79], Cristina Rodriguez-Antona[4, 5], Isabelle Romieu[80], Dale P. Sandler[81], Joellen M. Schildkraut[82], V. Wendy Setiawan[83], Kang Shan[84], Nadeem Siddiqui[85], Weiva Sieh[86, 87], Rebecca Sutphen[88], Anthony J. Swerdlow[89, 90], Soo Hwang Teo[91, 92], Kathryn L. Terry[17, 18], Shelley S. Tworoger[17, 63], Digna Velez Edwards[93], Roel C.H. Vermeulen[94], Penelope M. Webb[95], Nicolas Wentzensen[96], Emily White[32, 97], Walter Willett[17, 98, 99], Alicja Wolk[100, 101], Yin-Ling Woo[102], Anna H. Wu[83], Li Yan[103], Drakoulis Yannoukakos[104], Wei Zheng[105]

Affiliations: [1]Department of Medicine, Genetic Epidemiology Research Institute, University of California Irvine, Irvine, CA, USA, [2]Cancer Prevention and Control Program, Rutgers Cancer Institute of New Jersey, New Brunswick, NJ, USA, [3]Gynaecology Unit, Royal Marsden Hospital, London, UK, [4]Biomedical Network on Rare Diseases (CIBERER), Madrid, Spain, [5]Human Cancer Genetics Programme, Spanish National Cancer Research Centre (CNIO), Madrid, Spain, [6]Department of Gynecologic Oncology, Duke University Hospital, Durham, NC, USA, [7]Department of Obstetrics and Gynecology, Haukeland University Hospital, Bergen, Norway, [8]Centre for Cancer Biomarkers CCBIO, Department of Clinical Science, University of Bergen, Bergen, Norway, [9]Cancer Research UK Cambridge Institute, University of Cambridge, Cambridge, UK, [10]Department of Pathology, Helsinki University Hospital, University of Helsinki, Helsinki, Finland, [11]Peter MacCallum Cancer Center, Melbourne, Victoria, Australia, [12]Sir Peter MacCallum Department of Oncology, The University of Melbourne, Melbourne, Victoria, Australia, [13]Department of Epidemiology, Tianjin Medical University Cancer Institute and Hospital, Tianjin, China, [14]Department of Genetics and Computational Biology, QIMR Berghofer Medical Research Institute, Brisbane, Queensland, Australia, [15]University of New Mexico Health Sciences Center, University of New Mexico, Albuquerque, NM, USA, [16]Department of Cancer Epidemiology and Prevention Research, Alberta Health Services, Calgary, AB, Canada, [17]Department of Epidemiology, Harvard TH Chan School of Public Health, Boston, MA, USA, [18]Obstetrics and Gynecology Epidemiology Center, Brigham and Women's Hospital and Harvard Medical School, Boston, MA, USA, [19]Centre for Cancer Research, The Westmead Institute for Medical Research, The University of Sydney, Sydney, New South Wales, Australia, [20]Department of Gynaecological Oncology, Westmead Hospital, Sydney, New South Wales, Australia, [21]Huntsman Cancer Institute, Department of Population Health Sciences, University of Utah, Salt Lake City, UT, USA, [22]Gynaecology Research Unit, Hannover Medical School, Hannover, Germany, [23]Faculty of Medicine, University of Southampton, Southampton, UK, [24]David Geffen School of Medicine, Department of Medicine Division of Hematology and Oncology, University of California at Los Angeles, Los Angeles, CA, USA, [25]Department of Gynecology and Obstetrics, Comprehensive Cancer Center Erlangen-EMN, University Hospital Erlangen, Friedrich-Alexander University Erlangen-Nuremberg (FAU), Erlangen, Germany, [26]Division of Cancer Epidemiology, German Cancer Research Center (DKFZ), Heidelberg, Germany, [27]Center for Bioinformatics and Functional Genomics and the Cedars Sinai Genomics Core, Cedars-Sinai Medical Center, Los Angeles, CA, USA, [28]Department of Health Science Research, Division

of Epidemiology, Mayo Clinic, Rochester, MN, USA, [29]Samuel Oschin Comprehensive Cancer Institute, Cancer Prevention and Genetics Program, Cedars-Sinai Medical Center, Los Angeles, CA, USA, [30]Department of Genetics and Pathology, Pomeranian Medical University, Szczecin, Poland, [31]Program in Epidemiology, Division of Public Health Sciences, Fred Hutchinson Cancer Research Center, Seattle, WA, USA, [32]Department of Epidemiology, University of Washington, Seattle, WA, USA, [33]Humboldt-Universität zu Berlin, and Berlin Institute of Health, Department for Gynecology with the Center for Oncologic Surgery Charité Campus Virchow-Klinikum, Charité – Universitätsmedizin Berlin, corporate member of Freie Universität Berlin, Berlin, Germany, [34]Department of Gynecology and Gynecological Oncology; HSK, Dr Horst-Schmidt Klinik, Wiesbaden, Wiesbaden, Germany, [35]Department of Gynecology and Gynecologic Oncology, Ev Kliniken Essen-Mitte (KEM), Essen, Germany, [36]Department of Epidemiology, University of Texas MD Anderson Cancer Center, Houston, TX, USA, [37]Department of Virus, Lifestyle and Genes, Danish Cancer Society Research Center, Copenhagen, Denmark, [38]Molecular Unit, Department of Pathology, Herlev Hospital, University of Copenhagen, Copenhagen, Denmark, [39]Department of Gynaecology, Rigshospitalet, University of Copenhagen, Copenhagen, Denmark, [40]British Columbia's Ovarian Cancer Research (OVCARE) Program, BC Cancer, Vancouver General Hospital, and University of British Columbia, Vancouver, BC, Canada, [41]Department of Pathology and Laboratory Medicine, University of British Columbia, Vancouver, BC, Canada, [42]Department of Obstetrics and Gynecology, University of British Columbia, Vancouver, BC, Canada, [43]Department of Molecular Oncology, BC Cancer Research Centre, Vancouver, BC, Canada, [44]David Geffen School of Medicine, Department of Obstetrics and Gynecology, University of California at Los Angeles, Los Angeles, CA, USA, [45]Radboud Institute for Health Sciences, Radboud University Medical Center, Nijmegen, The Netherlands, [46]Department of Pathology and Laboratory Diagnostics, Maria Sklodowska-Curie National Research Institute of Oncology, Warsaw, Poland, [47]VIB Center for Cancer Biology, Leuven, Belgium, [48]Laboratory for Translational Genetics, Department of Human Genetics, University of Leuven, Leuven, Belgium, [49]Cancer Control Research, BC Cancer, Vancouver, BC, Canada, [50]Gynecology Service, Department of Surgery, Memorial Sloan Kettering Cancer Center, New York, NY, USA, [51]Gynecologic Oncology, Laura and Isaac Pearlmutter Cancer Center, NYU Langone Medical Center, New York, NY, USA, [52]Division of Cancer Epidemiology and Prevention, Aichi Cancer Center Research Institute, Nagoya, Japan, [53]Division of Cancer Epidemiology, Nagoya University Graduate School of Medicine, Nagoya, Japan, [54]Division of Gynecologic Oncology, University Health Network, Princess Margaret Hospital, Toronto, Ontario, Canada, [55]Division of Cancer and Ovarian Cancer Action Research Centre, Department Surgery & Cancer, Imperial College London, London, UK, [56]Institute of Cancer Sciences, University of Glasgow, Glasgow, UK, [57]Institute of Clinical Trials & Methodology, University College London, London, UK, [58]Cancer Epidemiology Division, Cancer Council Victoria, Melbourne, Victoria, Australia, [59]Centre for Epidemiology and Biostatistics, Melbourne School of Population and Global Health, The University of Melbourne, Melbourne, Victoria, Australia, [60]Precision Medicine, School of Clinical Sciences at Monash Health, Monash University, Clayton, Victoria, Australia, [61]Womens Cancer Research Center, Magee-Womens Research Institute and Hillman Cancer Center, Pittsburgh, PA, USA, [62]Division of Gynecologic Oncology, Department of Obstetrics, Gynecology and Reproductive Sciences, University of Pittsburgh School of Medicine, Pittsburgh, PA, USA, [63]Department of Cancer Epidemiology, Moffitt Cancer Center, Tampa, FL, USA, [64]Department of Community and Family Medicine, Duke University Hospital, Durham, NC, USA, [65]Division of Cancer Prevention and Control, Roswell Park Cancer Institute, Buffalo, NY, USA, [66]Department of Obstetrics and Gynecology, Helsinki University Hospital, University of Helsinki, Helsinki, Finland, [67]Department of Cancer Epidemiology, Clinical Sciences, Lund University, Lund, Sweden, [68]Department of Preventive Medicine, Seoul National University College of Medicine, Seoul, Korea, [69]Convergence Graduate Program in Innovative Medical Science, Seoul National University College of Medicine, Seoul, South Korea, [70]Cancer Research Institute, Seoul National University, Seoul, Korea,

[71]epartment of Epidemiology, University of Michigan School of Public Health, Ann Arbor, MI, USA, [72]Department of Preventive Medicine, Keck School of Medicine, University of Southern California Norris Comprehensive Cancer Center, Los Angeles, CA, USA, [73]Department of Obstetrics and Gynecology, Oregon Health & Science University, Portland, OR, USA, [74]Knight Cancer Institute, Oregon Health & Science University, Portland, OR, USA, [75]Department of Epidemiology and Biostatistics, Memorial Sloan-Kettering Cancer Center, New York, NY, USA, [76]School of Women's and Children's Health, Faculty of Medicine, University of NSW Sydney, Sydney, New South Wales, Australia, [77]Adult Cancer Program, Lowy Cancer Research Centre, University of NSW Sydney, Sydney, New South Wales, Australia, [78]Imperial College London, London, UK,[79]Chronic Disease Epidemiology, Yale School of Public Health, New Haven, CT, USA, [80]Nutrition and Metabolism Section, International Agency for Research on Cancer (IARC-WHO), Lyon, France, [81]Epidemiology Branch, National Institute of Environmental Health Sciences, NIH, Research Triangle Park, NC, USA, [82]Department of Epidemiology, Rollins School of Public Health, Emory University, Atlanta, GA, USA, [83]Department of Preventive Medicine, Keck School of Medicine, University of Southern California, Los Angeles, CA, USA, [84]Department of Obstetrics and Gynaecology, Hebei Medical University, Fourth Hospital, Shijiazhuang, China, [85]Department of Gynaecological Oncology, Glasgow Royal Infirmary, Glasgow, UK, [86]Department of Population Health Science and Policy, Icahn School of Medicine at Mount Sinai, New York, NY, USA, [87]Department of Genetics and Genomic Sciences, Icahn School of Medicine at Mount Sinai, New York, NY, USA, [88]Epidemiology Center, College of Medicine, University of South Florida, Tampa, FL, USA, [89]Division of Genetics and Epidemiology, The Institute of Cancer Research, London, UK, [90]Division of Breast Cancer Research, The Institute of Cancer Research, London, UK, [91]Breast Cancer Research Programme, Cancer Research Malaysia, Subang Jaya, Selangor, Malaysia,[92]Department of Surgery, Faculty of Medicine, University of Malaya, Kuala Lumpur, Malaysia, [93]Division of Quantitative Sciences, Department of Obstetrics and Gynecology, Department of Biomedical Sciences, Women's Health Research, Vanderbilt University Medical Center, Nashville, TN, USA, [94]Julius Center for Health Sciences and Primary Care, University Utrecht, UMC Utrecht, Utrecht, The Netherlands, [95]Population Health Department, QIMR Berghofer Medical Research Institute, Brisbane, Queensland, Australia, [96]Division of Cancer Epidemiology and Genetics, National Cancer Institute, Bethesda, MD, USA, [97]Fred Hutchinson Cancer Research Center, Seattle, WA, USA, 98109, [98]Department of Nutrition, Harvard TH Chan School of Public Health, Boston, MA, USA, [99]Channing Division of Network Medicine, Department of Medicine, Brigham and Women's Hospital and Harvard Medical School, Boston, MA, USA, [100]Institute of Environmental Medicine, Karolinska Institutet, Stockholm, Sweden, [101]Department of Surgical Sciences, Uppsala University, Uppsala, Sweden, [102]Department of Obstetrics and Gynaecology, University of Malaya Medical Centre, University of Malaya, Kuala Lumpur, Malaysia, [103]Department of Molecular Biology, Hebei Medical University, Fourth Hospital, Shijiazhuang, China, [104]Molecular Diagnostics Laboratory, INRASTES, National Centre for Scientific Research Demokritos, Athens, Greece, [105]Division of Epidemiology, Department of Medicine, Vanderbilt Epidemiology Center, Vanderbilt-Ingram Cancer Center, Vanderbilt University School of Medicine, Nashville, TN, USA

Pancreatic Cancer Cohort Consortium and Pancreatic Cancer Case Control Consortium (PanScan GWAS and PANC4 GWAS) Members:

Demetrius Albanes[1], Gabriella Andreotti[1], Alan A. Arslan[2,3,4], Laura Beane-Freeman[1], Julie Buring[5,6], Peter Campbell[7], Federico Canzian[8], Neal D. Freedman[1], J. Michael Gaziano[5,9,10], Graham G. Giles[11,12,13], Edward Giovannucci[14], Phyllis J. Goodman[15], Christopher Haiman[16], Eric J Jacobs[17], Verena Katzke[18], Manolis Kogevinas[19,20,21,22], Charles Kooperberg[23], Peter Kraft[6,24], Loic LeMarchand[25], Núria Malats[26], Roger L. Milne[11,12,13], Alpa V. Patel[27], Ulrike Peters[23], Elio Riboli[28], Howard D. Sesso[5,6], Xiao-Ou Shu[29], Malin Sund[30], Anne Tjønneland[31], Rosario Tumino[32], Kala Visvanathan[33], Jean Wactawski-Wende[34], Emily White[23,35], Anne Zeleniuch-Jacquotte[3,36], Wei Zheng[29], Stephen J. Chanock[1], Brian M. Wolpin[14], Rachael Z. Stolzenberg-Solomon[1], Laufey T. Amundadottir[37], Alison P Klein[38], Rayjean J Hung[39], Erica J Childs[38], Paige M Bracci[40], Steven Gallinger[39], Rachel E Neale[41], Mengmeng Du[42], William R Bamlet[43], Paul Brennan[44], Kari G Rabe[43], Manal Hassan[45], Elizabeth A Holly[40], Michael Goggins[38], Robert C Kurtz[46], Stephen Van Den Eeden[47], Sandra Perdomo[44], Gloria M Petersen[43], Harvey A Risch[48], Donghui Li[45]

Affiliations: [1]Division of Cancer Epidemiology and Genetics, National Cancer Institute, National Institutes of Health, Bethesda, MD, USA, [2]Department of Obstetrics and Gynecology, New York University School of Medicine, New York, NY, USA, [3]Department of Population Health, New York University School of Medicine, New York, NY, USA, [4]Department of Environmental Medicine, New York University School of Medicine, New York, NY, USA, [5]Division of Preventive Medicine, Brigham and Women's Hospital, Boston, MA, USA, [6]Department of Epidemiology, Harvard T.H. Chan School of Public Health, Boston, MA, USA, [7]Department of Population Science, American Cancer Society, Atlanta, GA, USA, [8]Genomic Epidemiology Group, German Cancer Research Center (DKFZ), Heidelberg, Germany, [9]Division of Aging, Brigham and Women's Hospital, Boston, MA, USA, [10]Boston VA Healthcare System, Boston, MA, USA, [11]Cancer Epidemiology Division, Cancer Council Victoria, Melbourne, VIC, Australia, [12]Centre for Epidemiology and Biostatistics, Melbourne School of Population and Global Health, The University of Melbourne, Parkville, VIC, Australia, [13]Precision Medicine, School of Clinical Sciences at Monash Health, Monash University, Melbourne, VIC, Australia, [14]Department of Medical Oncology, Dana-Farber Cancer Institute, Boston, MA, USA, [15]SWOG Statistical Center, Fred Hutchinson Cancer Research Center, Seattle, WA, USA, [16]Department of Preventive Medicine, Keck School of Medicine, University of Southern California, Los Angeles, CA, USA, [17]Behavioral and Epidemiology Research Group, American Cancer Society, Atlanta, GA, USA, [18]Division of Cancer Epidemiology, German Cancer Research Center (DKFZ), Heidelberg, Germany, [19]ISGlobal, Centre for Research in Environmental Epidemiology (CREAL), Barcelona, Spain, [20]CIBER Epidemiología y Salud Pública (CIBERESP), Barcelona, Spain, [21]Hospital del Mar Institute of Medical Research (IMIM), Universitat Autònoma de Barcelona, Barcelona, Spain, [22]Universitat Pompeu Fabra (UPF), Barcelona, Spain, [23]Division of Public Health Sciences, Fred Hutchinson Cancer Research Center, Seattle, WA, USA, [24]Department of Biostatistics, Harvard School of Public Health, Boston, MA, USA, [25]Cancer Epidemiology Program, University of Hawaii Cancer Center, Honolulu, HI, USA, [26]Genetic and Molecular Epidemiology Group, Spanish National Cancer Research Center (CNIO), Madrid, Spain, [27]Epidemiology Research Program, American Cancer Society, Atlanta, GA, USA, [28]Department of Epidemiology and Biostatistics, School of Public Health, Imperial College London, London, UK, [29]Division of Epidemiology, Department of Medicine, Vanderbilt Epidemiology Center, Vanderbilt-Ingram Cancer Center, Vanderbilt University Medical Center, Nashville, TN, USA, [30]Department of Surgical and Perioperative Sciences, Umeå University, Umeå, Sweden, [31]Danish Cancer Society Research Center, Copenhagen, Denmark, [32]Cancer Registry and Histopathology Department, Provincial Health Authority (ASP 7), Ragusa, Italy, [33]Department of Epidemiology, Johns Hopkins Bloomberg School of Public Health, Baltimore, MD, USA, [34]Department of Epidemiology and Environmental Health, University at Buffalo, Buffalo, NY, USA, [35]Department of Epidemiology,

University of Washington, Seattle, WA, USA, [36]Perlmutter Cancer Center, New York University School of Medicine, New York, NY, USA, [37]Laboratory of Translational Genomics, Division of Cancer Epidemiology and Genetics, National Cancer Institute, National Institutes of Health, Bethesda, MD, USA, [38]Department of Oncology and Pathology, Sidney Kimmel Comprehensive Cancer Center, Johns Hopkins School of Medicine, Baltimore, Maryland, USA, [39]Lunenfeld-Tanenbaum Research Institute, Sinai Health System and University of Toronto, Toronto, Ontario, Canada, [40]Department of Epidemiology and Biostatistics, University of California, San Francisco, San Francisco, California, USA, [41]Department of Population Health, QIMR Berghofer Medical Research Institute, Brisbane, Australia, [42]Department of Epidemiology and Biostatistics, Memorial Sloan Kettering Cancer Center, New York, New York, USA, [43]Department of Health Sciences Research, Mayo Clinic College of Medicine, Rochester, Minnesota, USA, [44]International Agency for Research on Cancer, Lyon, France, [45]Department of Gastrointestinal Medical Oncology, University of Texas MD Anderson Cancer Center, Houston, Texas, USA, [46]Department of Gastroenterology, Hepatology, and Nutrition Service, Memorial Sloan Kettering Cancer Center, New York, New York, USA, [47]Division of Research, Kaiser Permanente Northern California, Oakland, CA, USA, [48]Department of Chronic Disease Epidemiology, Yale School of Public Health, New Haven, Connecticut, USA.

PRACTICAL consortium, CRUK, BPC3, CAPS and PEGASUS Members

Rosalind A. Eeles[1,2], Christopher A. Haiman[3], Zsofia Kote-Jarai[1], Fredrick R. Schumacher[4,5], Sara Benlloch[6,1], Ali Amin Al Olama[6,7], Kenneth Muir[8,9], Sonja I. Berndt[10], David V. Conti[3], Fredrik Wiklund[11], Stephen Chanock[10], Ying Wang[12], Catherine M. Tangen[13], Jyotsna Batra[14,15], Judith A. Clements[14,15], APCB BioResource (Australian Prostate Cancer BioResource)[14,15], Henrik Grönberg[11], Nora Pashayan[16,17], Johanna Schleutker[18,19], Demetrius Albanes[10], Stephanie Weinstein[10], Alicja Wolk[20,21], Catharine M. L. West[22], Lorelei A. Mucci[23], Géraldine Cancel-Tassin[24,25], Stella Koutros[10], Karina Dalsgaard Sørensen[26,27], Eli Marie Grindedal[28], David E. Neal[29,30,31], Freddie C. Hamdy[32,33], Jenny L. Donovan[34], Ruth C. Travis[35], Robert J. Hamilton[36,37], Sue Ann Ingles[38], Barry S. Rosenstein[39,40], Yong-Jie Lu[41], Graham G. Giles[42,43,44], Adam S. Kibel[45], Ana Vega[46,47,48], Manolis Kogevinas[49,50,51,52], Kathryn L. Penney[53], Jong Y. Park[54], Janet L. Stanford[55,56], Cezary Cybulski[57], Børge G. Nordestgaard[58,59], Sune F. Nielsen[58,59], Hermann Brenner[60,61,62], Christiane Maier[63], Jeri Kim[64], Esther M. John[65], Manuel R. Teixeira[66,67,68], Susan L. Neuhausen[69], Kim De Ruyck[70], Azad Razack[71], Lisa F. Newcomb[55,72], Davor Lessel[73], Radka Kaneva[74], Nawaid Usmani[75,76], Frank Claessens[77], Paul A. Townsend[78,79], Manuela Gago-Dominguez[80,81], Monique J. Roobol[82], Florence Menegaux[83], Kay-Tee Khaw[84], Lisa Cannon-Albright[85,86], Hardev Pandha[79], Stephen N. Thibodeau[87], David J. Hunter[88], Peter Kraft[89], William J. Blot[90,91], Elio Riboli[92]

Affiliations: [1]The Institute of Cancer Research, London, SM2 5NG, UK, [2] Royal Marsden NHS Foundation Trust, London, SW3 6JJ, UK, [3] Center for Genetic Epidemiology, Department of Preventive Medicine, Keck School of Medicine, University of Southern California/Norris Comprehensive Cancer Center, Los Angeles, CA 90015, USA, [4] Department of Population and Quantitative Health Sciences, Case Western Reserve University, Cleveland, OH 44106-7219, USA, [5] Seidman Cancer Center, University Hospitals, Cleveland, OH 44106, USA, [6] Centre for Cancer Genetic Epidemiology, Department of Public Health and Primary Care, University of Cambridge, Strangeways Research Laboratory, Cambridge CB1 8RN, UK, [7] University of Cambridge, Department of Clinical Neurosciences, Stroke Research Group, R3, Box 83, Cambridge Biomedical Campus, Cambridge CB2 0QQ, UK, [8] Division of Population Health, Health Services Research and Primary Care, University of Manchester, Oxford Road, Manchester, M13 9PL, UK, [9] Warwick Medical School, University of Warwick, Coventry, CV4 7AL, UK, [10] Division of Cancer Epidemiology and Genetics, National Cancer Institute, NIH, Bethesda, Maryland, 20892, USA, [11] Department of Medical Epidemiology and Biostatistics, Karolinska Institute, SE-171 77 Stockholm, Sweden, 12 Department of Population Science, American Cancer Society, 250 Williams Street, Atlanta, GA 30303, USA, [13] SWOG Statistical Center, Fred Hutchinson Cancer Research Center, Seattle, WA 98109, USA, [14] Australian Prostate Cancer Research Centre-Qld, Institute of Health and Biomedical Innovation and School of Biomedical Sciences, Queensland University of Technology, Brisbane QLD 4059, Australia, [15] Translational Research Institute, Brisbane, Queensland 4102, Australia, [16] Department of Applied Health Research, University College London, London, WC1E 7HB, UK, [17] Centre for Cancer Genetic Epidemiology, Department of Oncology, University of Cambridge, Strangeways Laboratory, Worts Causeway, Cambridge, CB1 8RN, UK, [18] Institute of Biomedicine, University of Turku, Finland, [19] Department of Medical Genetics, Genomics, Laboratory Division, Turku University Hospital, PO Box 52, 20521 Turku, Finland, [20] Unit of Cardiovascular and Nutritional Epidemiology, Institute of Environmental Medicine, Karolinska Institutet, SE-171 77 Stockholm, Sweden, [21] Department of Surgical Sciences, Uppsala University, 75185 Uppsala, Sweden, [22] Division of Cancer Sciences, University of Manchester, Manchester Academic Health Science Centre, Radiotherapy Related Research, The Christie Hospital NHS Foundation Trust, Manchester, M13 9PL UK, [23] Department of Epidemiology, Harvard T. H. Chan School of Public Health, Boston, MA 02115, USA, [24] CeRePP, Tenon Hospital, F-75020 Paris, France, [25] Sorbonne Universite, GRC n°5 , AP-HP, Tenon Hospital, 4 rue de la Chine, F-75020 Paris, France, [26] Department of Molecular Medicine, Aarhus University Hospital, Palle Juul-Jensen Boulevard 99, 8200 Aarhus N,

Denmark, [27] Department of Clinical Medicine, Aarhus University, DK-8200 Aarhus N, Denmark [28] Department of Medical Genetics, Oslo University Hospital, 0424 Oslo, Norway, [29] Nuffield Department of Surgical Sciences, University of Oxford, Room 6603, Level 6, John Radcliffe Hospital, Headley Way, Headington, Oxford, OX3 9DU, UK, [30] University of Cambridge, Department of Oncology, Box 279, Addenbrooke's Hospital, Hills Road, Cambridge CB2 0QQ, UK, [31] Cancer Research UK, Cambridge Research Institute, Li Ka Shing Centre, Cambridge, CB2 0RE, UK, [32] Nuffield Department of Surgical Sciences, University of Oxford, Oxford, OX1 2JD, UK, [33] Faculty of Medical Science, University of Oxford, John Radcliffe Hospital, Oxford, UK, [34] Population Health Sciences, Bristol Medical School, University of Bristol, BS8 2PS, UK, [35] Cancer Epidemiology Unit, Nuffield Department of Population Health, University of Oxford, Oxford, OX3 7LF, UK, [36] Dept. of Surgical Oncology, Princess Margaret Cancer Centre, Toronto ON M5G 2M9, Canada, [37] Dept. of Surgery (Urology), University of Toronto, Canada, [38] Department of Preventive Medicine, Keck School of Medicine, University of Southern California/Norris Comprehensive Cancer Center, Los Angeles, CA 90015, USA, [39] Department of Radiation Oncology and Department of Genetics and Genomic Sciences, Box 1236, Icahn School of Medicine at Mount Sinai, One Gustave L. Levy Place, New York, NY 10029, USA, [40] Department of Genetics and Genomic Sciences, Icahn School of Medicine at Mount Sinai, New York, NY 10029-5674 , USA, [41] Centre for Cancer Biomarker and Biotherapeutics, Barts Cancer Institute, Queen Mary University of London, John Vane Science Centre, Charterhouse Square, London, EC1M 6BQ, UK, [42] Cancer Epidemiology Division, Cancer Council Victoria, 615 St Kilda Road, Melbourne, VIC 3004, Australia, [43] Centre for Epidemiology and Biostatistics, Melbourne School of Population and Global Health, The University of Melbourne, Grattan Street, Parkville, VIC 3010, Australia, [44] Precision Medicine, School of Clinical Sciences at Monash Health, Monash University, Clayton, Victoria 3168, Australia, [45] Division of Urologic Surgery, Brigham and Womens Hospital, 75 Francis Street, Boston, MA 02115, USA, [46] Fundación Pública Galega Medicina Xenómica, Santiago de Compostela, 15706, Spain, [47] Instituto de Investigación Sanitaria de Santiago de Compostela, Santiago De Compostela, 15706, Spain, [48] Centro de Investigación en Red de Enfermedades Raras (CIBERER), Spain, [49] ISGlobal, Barcelona, Spain, [50] IMIM (Hospital del Mar Medical Research Institute), Barcelona, Spain, [51] Universitat Pompeu Fabra (UPF), Barcelona, Spain, [52] CIBER Epidemiología y Salud Pública (CIBERESP), 28029 Madrid, Spain,[53] Channing Division of Network Medicine, Department of Medicine, Brigham and Women's Hospital/Harvard Medical School, Boston, MA 02115, USA, [54] Department of Cancer Epidemiology, Moffitt Cancer Center, 12902 Magnolia Drive, Tampa, FL 33612, USA, [55] Division of Public Health Sciences, Fred Hutchinson Cancer Research Center, Seattle, Washington, 98109-1024, USA, [56] Department of Epidemiology, School of Public Health, University of Washington, Seattle, Washington 98195, USA, [57] International Hereditary Cancer Center, Department of Genetics and Pathology, Pomeranian Medical University, 70-115 Szczecin, Poland, [58] Faculty of Health and Medical Sciences, University of Copenhagen, 2200 Copenhagen, Denmark, [59] Department of Clinical Biochemistry, Herlev and Gentofte Hospital, Copenhagen University Hospital, Herlev, 2200 Copenhagen, Denmark, [60] Division of Clinical Epidemiology and Aging Research, German Cancer Research Center (DKFZ), D-69120, Heidelberg, Germany, [61] German Cancer Consortium (DKTK), German Cancer Research Center (DKFZ), D-69120 Heidelberg, Germany, [62] Division of Preventive Oncology, German Cancer Research Center (DKFZ) and National Center for Tumor Diseases (NCT), Im Neuenheimer Feld 460, 69120 Heidelberg, Germany, [63] Humangenetik Tuebingen, Paul-Ehrlich-Str 23, D-72076 Tuebingen, Germany, [64] The University of Texas M. D. Anderson Cancer Center, Department of Genitourinary Medical Oncology, 1515 Holcombe Blvd., Houston, TX 77030, USA, [65] Departments of Epidemiology & Population Health and of Medicine, Division of Oncology, Stanford Cancer Institute, Stanford University School of Medicine, Stanford, CA 94304 USA, [66] Department of Genetics, Portuguese Oncology Institute of Porto (IPO-Porto), 4200-072 Porto, Portugal, [67] Biomedical Sciences Institute (ICBAS), University of Porto, 4050-313 Porto, Portugal, [68] Cancer Genetics Group, IPO-Porto Research

Center (CI-IPOP), Portuguese Oncology Institute of Porto (IPO-Porto), 4200-072 Porto, Portugal, [69] Department of Population Sciences, Beckman Research Institute of the City of Hope, 1500 East Duarte Road, Duarte, CA 91010, 626-256-HOPE (4673), [70] Ghent University, Faculty of Medicine and Health Sciences, Basic Medical Sciences, Proeftuinstraat 86, B-9000 Gent, Belgium, [71] Department of Surgery, Faculty of Medicine, University of Malaya, 50603 Kuala Lumpur, Malaysia, [72] Department of Urology, University of Washington, 1959 NE Pacific Street, Box 356510, Seattle, WA 98195, USA, [73] Institute of Human Genetics, University Medical Center Hamburg-Eppendorf, D-20246 Hamburg, Germany, [74] Molecular Medicine Center, Department of Medical Chemistry and Biochemistry, Medical University of Sofia, Sofia, 2 Zdrave Str., 1431 Sofia, Bulgaria, [75] Department of Oncology, Cross Cancer Institute, University of Alberta, 11560 University Avenue, Edmonton, Alberta, Canada T6G 1Z2, [76] Division of Radiation Oncology, Cross Cancer Institute, 11560 University Avenue, Edmonton, Alberta, Canada T6G 1Z2, [77] Molecular Endocrinology Laboratory, Department of Cellular and Molecular Medicine, KU Leuven, BE-3000, Belgium, [78] Division of Cancer Sciences, Manchester Cancer Research Centre, Faculty of Biology, Medicine and Health, Manchester Academic Health Science Centre, NIHR Manchester Biomedical Research Centre, Health Innovation Manchester, Univeristy of Manchester, M13 9WL, [79] The University of Surrey, Guildford, Surrey, GU2 7XH, UK, [80] Genomic Medicine Group, Galician Foundation of Genomic Medicine, Instituto de Investigacion Sanitaria de Santiago de Compostela (IDIS), Complejo Hospitalario Universitario de Santiago, Servicio Galego de Saúde, SERGAS, 15706, Santiago de Compostela, Spain, [81] University of California San Diego, Moores Cancer Center, Department of Family Medicine and Public Health, University of California San Diego, La Jolla, CA 92093-0012, USA, [82] Department of Urology, Erasmus University Medical Center, 3015 CE Rotterdam, The Netherlands, [83] "Exposome and Heredity", CESP (UMR 1018), Faculté de Médecine, Université Paris-Saclay, Inserm, Gustave Roussy, Villejuif, [84] Clinical Gerontology Unit, University of Cambridge, Cambridge, CB2 2QQ, UK, [85] Division of Epidemiology, Department of Internal Medicine, University of Utah School of Medicine, Salt Lake City, Utah 84132, USA, [86] George E. Wahlen Department of Veterans Affairs Medical Center, Salt Lake City, Utah 84148, USA, [87] Department of Laboratory Medicine and Pathology, Mayo Clinic, Rochester, MN 55905, USA, [88] Nuffield Department of Population Health, University of Oxford, United Kingdom, [89] Program in Genetic Epidemiology and Statistical Genetics, Department of Epidemiology, Harvard School of Public Health, Boston, MA, USA, [90] Division of Epidemiology, Department of Medicine, Vanderbilt University Medical Center, 2525 West End Avenue, Suite 800, Nashville, TN 37232 USA, [91] International Epidemiology Institute, Rockville, MD 20850, USA, [92] Department of Epidemiology and Biostatistics, School of Public Health, Imperial College London, SW7 2AZ, UK

Renal Cancer GWAS Consortium Members

Ghislaine Scelo[1], Mark P Purdue[2], Kevin M Brown[2], Mattias Johansson[1], Zhaoming Wang[3], Jeanette E Eckel-Passow[4], Yuanqing Ye[5], Jonathan N Hofmann[2], Jiyeon Choi[2], Matthieu Foll[1], Valerie Gaborieau[1], Mitchell J Machiela[2], Leandro M Colli[2], Peng Li[1], Joshua N Sampson[2], Behnoush Abedi-Ardekani[1], Celine Besse[6], Helene Blanche[7], Anne Boland[6], Laurie Burdette[2], Amelie Chabrier[1], Geoffroy Durand[1], Florence Le Calvez-Kelm[1], Egor Prokhortchouk[8,9], Nivonirina Robinot[1], Konstantin G Skryabin[8,9], Magdalena B Wozniak[1], Meredith Yeager[2], Gordana Basta-Jovanovic[10], Zoran Dzamic[11], Lenka Foretova[12], Ivana Holcatova[13], Vladimir Janout[14], Dana Mates[15], Anush Mukeriya[16], Stefan Rascu[17], David Zaridze[16], Vladimir Bencko[18], Cezary Cybulski[19], Eleonora Fabianova[20], Viorel Jinga[17], Jolanta Lissowska[21], Jan Lubinski[19], Marie Navratilova[12], Peter Rudnai[22], Neonila Szeszenia-Dabrowska[23], Simone Benhamou[24], Geraldine Cancel-Tassin[26,27], Olivier Cussenot[26,27], Laura Baglietto[28], Heiner Boeing[29], Kay-Tee Khaw[30], Elisabete Weiderpass[31,32,33,34], Borje Ljungberg[35], Raviprakash T Sitaram[35],Fiona Bruinsma[36], Susan J Jordan[37,38], Gianluca Severi[28,36,39,40], Ingrid Winship[41,42], Kristian Hveem[43], Lars J Vatten[44], Tony Fletcher[45], Kvetoslava Koppova[20], Susanna C Larsson[46], Alicja Wolk[46], Rosamonde E Banks[47], Peter J Selby[47], Douglas F Easton[30,48], Paul Pharoah[30,48], Gabriella Andreotti[2], Laura E Beane Freeman[2], Stella Koutros[2], Demetrius Albanes[2], Satu Männistö[49], Stephanie Weinstein[2], Peter E Clark[50], Todd L Edwards[51], Loren Lipworth[52], Susan M Gapstur[53], Victoria L Stevens[53], Hallie Carol[54], Matthew L Freedman[54], Mark M Pomerantz[54], Eunyoung Cho[55], Peter Kraft[56], Mark A Preston[57], Kathryn M Wilson[56], J Michael Gaziano[57], Howard D Sesso[56,57], Amanda Black[2], Neal D Freedman[2], Wen-Yi Huang[2], John G Anema[58], Richard J Kahnoski[58], Brian R Lane[58,59], Sabrina L Noyes[60], David Petillo[60], Bin Tean Teh[60], Ulrike Peters[61], Emily White[61], Garnet L Anderson[61], Lisa Johnson[61], Juhua Luo[62], Julie Buring[56,57], I-Min Lee[56,57], Wong-Ho Chow[5], Lee E Moore[2], Christopher Wood[63], Timothy Eisen[64], Marc Henrion[65], James Larkin[66], Poulami Barman[4], Bradley C Leibovich[67], Toni K Choueiri[54], G Mark Lathrop[68], Nathaniel Rothman[2], Jean-Francois Deleuze[6,7], James D McKay[1], Alexander S Parker[69], Xifeng Wu[5], Richard S Houlston[70,71], Paul Brennan[1], Stephen J Chanock[2]

Affiliations: [1]International Agency for Research on Cancer (IARC), 69008 Lyon, France, [2]Division of Cancer Epidemiology and Genetics, National Cancer Institute, National Institutes of Health, Department Health and Human Services, Bethesda, Maryland 20892, USA, [3]Department of Computational Biology, StJude Children's Research Hospital, Memphis, Tennessee 38105, USA, [4]Department of Health Sciences Research, Mayo Clinic, Rochester, Minnesota 55905, USA, [5]Department of Epidemiology, Division of Cancer Prevention and Population Sciences, The University of Texas MD Anderson Cancer Center, Houston, Texas 77230, USA, [6]Centre National de Genotypage, Institut de Genomique, Commissariat à l'Energie Atomique et aux Energies Alternatives, 91057 Evry, France, [7]Fondation Jean Dausset-Centre d'Etude du Polymorphisme Humain, 75010 Paris, France, [8]Center 'Bioengineering' of the Russian Academy of Sciences, Moscow 117312, Russian Federation, [9]Kurchatov Scientific Center, Moscow 123182, Russian Federation, 10Institute of Pathology, School of Medicine, University of Belgrade, 11000 Belgrade, Serbia, [11]Clinical Center of Serbia (KCS), Clinic of Urology, University of Belgrade - Faculty of Medicine, 11000 Belgrade, Serbia, [12]Department of Cancer Epidemiology and Genetics, Masaryk Memorial Cancer Institute, 656 53 Brno, Czech Republic, 132nd Faculty of Medicine, Institute of Public Health and Preventive Medicine, Charles University, 150 06 Prague 5, Czech Republic, [14]Department of Preventive Medicine, Faculty of Medicine, Palacky University, 775 15 Olomouc, Czech Republic, [15]National Institute of Public Health, 050463 Bucharest, Romania, [16]Russian N.NBlokhin Cancer Research Centre, Moscow 115478, Russian Federation, [17]Carol Davila University of Medicine and Pharmacy, ThBurghele Hospital, 050659 Bucharest, Romania, [18]First Faculty of Medicine, Institute of Hygiene and Epidemiology, Charles University, 128 00 Prague 2, Czech Republic, [19]International Hereditary Cancer

Center, Department of Genetics and Pathology, Pomeranian Medical University, 70-204 Szczecin, Poland, [20]Regional Authority of Public Health in Banska Bystrica, 975 56 Banska Bystrica, Slovakia, [21]The M Sklodowska-Curie Cancer Center and Institute of Oncology, 02-034 Warsaw, Poland, [22]National Public Health Center, National Directorate of Environmental Health, 1097 Budapest, Hungary, [23]Department of Epidemiology, Institute of Occupational Medicine, 91-348 Lodz, Poland, [24]Université Paris Diderot, INSERM, Unité Variabilité Génétique et Maladies Humaines, 75010 Paris, France, [26]CeRePP, Tenon Hospital, 75020 Paris, France , [27]UPMC Univ Paris 06 GRC n°5, 75013 Paris, France, [28]Centre de Recherche en Épidémiologie et Santé des Populations (CESP, Inserm U1018), Université Paris-Saclay, UPS, UVSQ, Gustave Roussy, 94805 Villejuif, France, [29]Department of Epidemiology, German Institute of Human Nutrition (DIfE) Potsdam-Rehbrücke, 14558 Nuthetal, Germany, [30]Department of Public Health and Primary Care, University of Cambridge, Cambridge CB2 0QQ, UK, [31]Department of Community Medicine, Faculty of Health Sciences, University of Tromsø, The Arctic University of Norway, 9037 Tromsø, Norway., [32]Department of Research, Cancer Registry of Norway, Institute of Population-Based Cancer Research, 0304 Oslo, Norway., [33]Department of Medical Epidemiology and Biostatistics, Karolinska Institutet, 171 77 Stockholm, Sweden, [34]Genetic Epidemiology Group, Folkhälsan Research Center, 00250 Helsinki, Finland, [35]Department of Surgical and Perioperative Sciences, Urology and Andrology, Umeå University, 901 85 Umeå, Sweden, [36]Cancer Epidemiology Centre, Cancer Council Victoria, Melbourne, Victoria, 3004, Australia, [37]QIMR Berghofer Medical Research Institute, Herston, Queensland, 4006, Australia, [38]School of Public Health, The University of Queensland, Brisbane, Queensland, 4072, Australia, [39]Centre for Epidemiology and Biostatistics, Melbourne School of Population and Global Health, The University of Melbourne, Carlton, Victoria, 3053, Australia, [40]Human Genetics Foundation (HuGeF), 10126 Torino, Italy, [41]Department of Medicine, The University of Melbourne, Melbourne, Victoria, 3010, Australia, [42]Genetic Medicine and Family Cancer Clinic, Royal Melbourne Hospital, Parkville, Victoria, 3050, Australia, [43]HUNT Research Centre, Department of Public Health and General Practice, Norwegian University of Science and Technology, Levanger, 7600, Norway, [44]Department of Public Health and General Practice, Faculty of Medicine, Norwegian University of Science and Technology, Trondheim, 7491, Norway, [45]London School of Hygiene and Tropical Medicine, University of London, London WC1H 9SH, UK, [46]Institute of Environmental Medicine, Karolinska Institutet, 171 77 Stockholm, Sweden, [47]Leeds Institute of Cancer and Pathology, University of Leeds, Cancer Research Building, St James's University Hospital, Leeds LS9 7TF, UK, [48]Department of Oncology, University of Cambridge, Cambridge CB1 8RN, UK, [49]Department of Health, National Institute for Health and Welfare, 00271 Helsinki, Finland, [50]Vanderbilt-Ingram Cancer Center, Department of Urology, Vanderbilt University Medical Center, Nashville, Tennessee 37232, USA, [51]Vanderbilt-Ingram Cancer Center, Division of Epidemiology, Department of Medicine, Institute for Medicine and Public Health, Vanderbilt Genetics Institute, Vanderbilt University Medical Center, Nashville, Tennessee 37209, USA, [52]Vanderbilt-Ingram Cancer Center, Division of Epidemiology, Department of Medicine, Institute for Medicine and Public Health, Vanderbilt University Medical Center, Nashville, Tennessee 37203, USA, [53]American Cancer Society, Atlanta, Georgia 30303, USA, 54Dana-Farber Cancer Institute, Boston, Massachusetts 02215, USA, [55]Warren Alpert Medical School of Brown University, Providence, Rhode Island 02903, USA, [56]Harvard T.HChan School of Public Health, Boston, Massachusetts 02115, USA, [57]Brigham and Women's Hospital and VA Boston, Boston, Massachusetts 02115, USA, [58]Division of Urology, Spectrum Health, Grand Rapids, Michigan 49503, USA, [59]College of Human Medicine, Michigan State University, Grand Rapids, Michigan 49503, USA, [60]Van Andel Research Institute, Center for Cancer Genomics and Quantitative Biology, Grand Rapids, Michigan 49503, USA, [61]Cancer Prevention Program, Fred Hutchinson Cancer Research Center, Seattle, Washington 98109, USA, [62]Department of Epidemiology and Biostatistics, School of Public Health Indiana University Bloomington, Bloomington, Indiana 47405, USA, [63]Department of Urology, The University of Texas M. D. Anderson Cancer Center,

Houston, TX 77030, USA, [64]Department of Oncology, Cambridge University Hospitals NHS Foundation Trust, Cambridge CB2 0QQ, UK, [65]Department of Genetics and Genomic Sciences, Icahn School of Medicine at Mount Sinai, New York, New York 10029, USA, [66]Medical Oncology, Royal Marsden NHS Foundation Trust, London SW3 6JJ, UK, [67]Department of Urology, Mayo Medical School and Mayo Clinic, Rochester, Minnesota 55902, USA, [68]McGill University and Genome Quebec Innovation Centre, Montreal, Quebec H3A 0G1, Canada, [69]Department of Health Sciences Research, Mayo Clinic, Jacksonville, Florida 32224, USA, [70]Division of Genetics and Epidemiology, The Institute of Cancer Research, London SW7 3RP, UK, [71]Division of Molecular Pathology, The Institute of Cancer Research, London SW7 3RP, UK
